# Supplementary material for: Asymmetric total synthesis of yuzurimine-type Daphniphyllum alkaloid (+)-caldaphnidine J
Source: Nat Commun. 2020 Jul 15;11:3538. doi: 10.1038/s41467-020-17350-x (PMC7363893; doi:10.1038/s41467-020-17350-x)
Supplement: Supplementary file 1 — Supplementary Information [file 41467_2020_17350_MOESM1_ESM.pdf]

# Supplementary Information

## Asymmetric Total Synthesis of Yuzurimine-type *Daphniphyllum* Alkaloid (+)-Caldaphnidine J

Guo *et al.*

# Supplementary Note 1

## General Information

The following abbreviations were used: **PE**: petroleum ether; **EtOAc**: ethyl acetate; **THF**: Tetrahydrofuran; **DCM**: dichloromethane; **TEA**: triethylamine; **KHMDS**: potassium bis(trimethylsilyl)amide; ***p*-TsOH**: *p*-toluenesulfonic acid monohydrate; **$PhNTf_2$**: *N*-phenyl-bis(trifluoromethanesulfonimide); **TFA**: trifluoroacetic acid **DMSO**: dimethyl sulfoxide, **Im**: 1*H*-imidazole; **Py**: pyridine **LDA**: lithium diisopropylamide; **dppp:** 1,3-bis(diphenylphosphino) propane **DCE**: 1,2-dichloroethane **$Ac_2O$**: acetic anhydride **DMAP**: 4-dimethylaminopyridine; **DDQ**: 2,3-dichloro-5,6-dicyano-1,4-benzoquinone; **DIBAL-H**: diisobutylaluminium hydride; **TFAA**: trifluoroacetic anhydride; **DBU**: 1,8-diazabicyclo[5.4.0]undec-7-ene; **Na-Naph**: sodium-naphthalenide; **DME**: 1,2-dimethoxyethane; **AIBN**: 2,2'-azobis(2-methylpropionitrile).

# Supplementary Note 2

## Procedure to prepare the precursors of carbocyclization cascade and Nazarov cyclization.

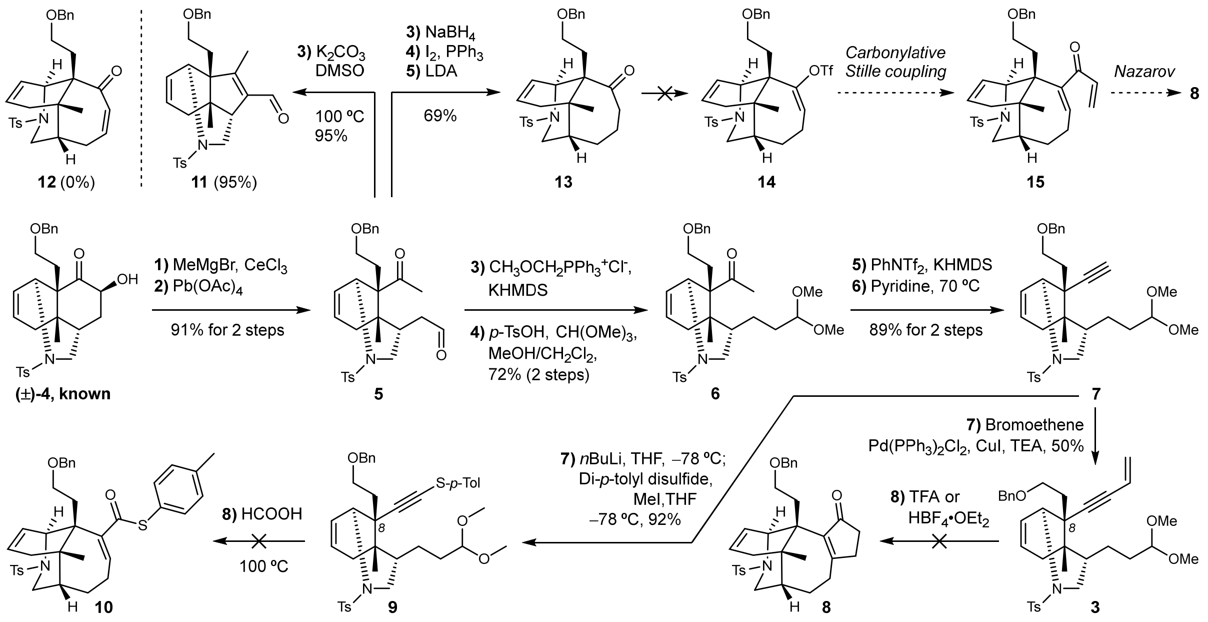


**Supplementary Figure 1.** Synthetic route of precursors

### Synthesis of compound 5

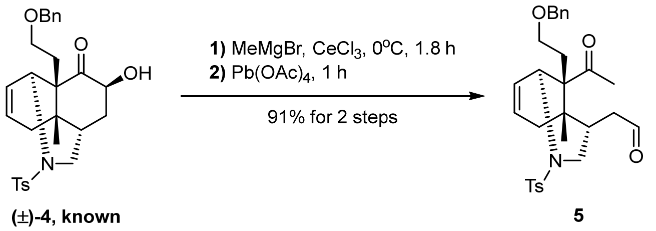


In a glovebox, a flask was charged with dry $CeCl_3$ (7.39 g, 30.0 mmol, 6.0 equiv) and then flushed with argon, and dry THF (75 mL) was added. After being stirred for 2 h at room temperature, the reaction mixture was cooled to 0 °C. Methyl magnesium bromide (30 mL, 30.0 mmol, 1.0 M in THF, 6.0 equiv) was added dropwise to this stirred suspension at 0 °C. The resulting mixture was stirred at the same temperature for 1.5 h providing a yellow-orange cloudy solution of the organocerium reagent, then a solution of ketone **4**[1,2] (2.48 g, 5.00 mmol, 1.0 equiv) in THF (10 mL) was added over 20 min. After being stirred for 1.5 h at 0 °C, 2 N HCl (40 mL) was added. THF was removed under reduced pressure, and then the aqueous phase was extracted with EtOAc (3 × 100 mL). The combined organic phases were washed with brine, dried over anhydrous $Na_2SO_4$, filtered, and concentrated under reduced pressure. The crude product (2.62 g) was used directly for the next step without further purification.

To a solution of the aforementioned crude product (2.62 g) in DCM (100 mL) was added $Pb(OAc)_4$ (3.33 g, 7.50 mmol, 1.5 equiv). After being stirred for 40 min at room temperature, saturated aqueous $NaHCO_3$ (150 mL) was added. The resulting mixture was stirred for 1 h at room temperature. The reaction mixture was filtered through Celite (eluent: EtOAc). The organic phase was separated and the aqueous phase was extracted with EtOAc (3 × 100 mL). The combined organic phases were washed with brine, dried over anhydrous $Na_2SO_4$, filtered, and concentrated under reduced pressure. The residue was purified by flash chromatography on silica gel (eluent: EtOAc/PE = 1/4 to 1/3) to give compound **5** (2.32 g, yield: 91%, over 2 steps) as a yellow foam.

**Rf** = 0.60 (silica, EtOAc/PE = 1:2);

**$^1$H NMR** (400 MHz, $CDCl_3$) δ 9.68 (s, 1H), 7.59 (d, *J* = 7.6 Hz, 2H), 7.42 – 7.29 (m, 5H), 7.27 – 7.25 (d, *J* = 7.6 Hz, 2H), 5.83 – 5.75 (m, 1H), 5.09 (d, *J* = 6.1 Hz, 1H), 5.05 – 4.98 (m, 1H), 4.45 (d, *J* =12.7 Hz, 1H), 4.42 (d, *J* =12.67 Hz, 1H ), 3.45 – 3.36 (m, 2H), 3.15 (d, *J* = 12.2 Hz, 1H), 2.98 (d, *J* = 11.6 Hz, 1H), 2.64 (s, 3H), 2.59 (d, *J* = 19.3 Hz, 1H), 2.50 – 2.43 (m, 1H), 2.42 (s, 3H), 2.13 (d, *J* = 19.9 Hz, 1H), 2.07 – 1.97 (m, 2H), 1.82 (d, *J* = 19.8 Hz, 1H), 1.75 – 1.64 (m, 1H), 1.18 (s, 3H) ppm;

**$^{13}$C NMR** (101 MHz, $CDCl_3$) δ 214.71, 200.83, 143.67, 137.73, 135.67, 133.31, 129.57 (2C), 128.39 (2C), 127.77 (2C), 127.65 (3C), 118.49, 73.20, 66.43, 54.35, 53.00, 45.31, 43.16, 42.63, 39.89, 38.07, 33.93, 31.38, 23.44, 21.50 ppm;

**HRMS** (ESI): calculated for $[C_{29}H_{35}NO_5S+H]^+$ 510.2309, found 510.2312.

### Synthesis of compound 6

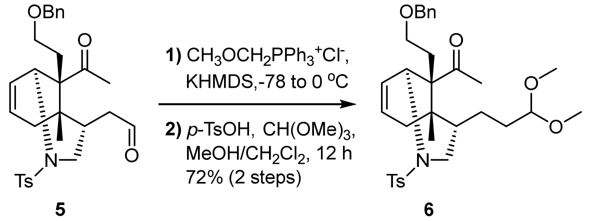


A solution of methoxymethyl triphenylphosphonium chloride (5.65 g, 16.5 mmol, 4.0 equiv) in dry THF (60 mL) was cooled to −78 °C and KHMDS (31.4 mL, 0.5 M in toluene, 3.8 equiv) was added dropwise. The resulting red suspension was allowed to warm up to 0 °C and stirred for 1 h. A solution of aldehyde **5** (2.10 g, 4.12 mmol, 1.0 equiv) in THF (20 mL) was added via cannula over 10 min at −78 °C. After being stirred for 1.5 h at that temperature, the reaction was warmed up to 0 °C and stirred

for 1 h. The reaction mixture was then quenched with saturated aqueous $NaHCO_3$ (50 mL) and extracted with EtOAc (3 × 80 mL). The combined organics were washed with brine (40 mL), dried over $MgSO_4$, and concentrated in vacuo. The resulting residue was used directly for the next step without further purification.

To a solution of aforementioned product and $CH(OMe)_3$ (20 mL) in DCM/MeOH (1/1, 100 mL), was added *p*-TsOH (158 mg, 0.82 mmol, 0.2 equiv). The reaction was stirred for 12 h and quenched with TEA (5 mL). The mixture was concentrated and the resulting residue was purified by flash column on silica gel (eluent: EtOAc/PE = 1/8 to 1/4) to give compound **6** (1.69 g, yield: 72%, over two steps) as a white foam.

**Rf** = 0.42 (silica, EtOAc/PE = 1:4);

**$^1$H NMR** (400 MHz, $CDCl_3$) δ 7.65 (d, *J* = 8.2 Hz, 2H), 7.38 (m, 4H), 7.31 (m, 1H), 7.29 – 7.24 (m, 2H), 5.79 (m, 1H), 5.12 – 5.01 (m, 2H), 4.44 (d, *J* = 12.2 Hz, 1H), 4.42 (d, *J* = 12.2, 1H) 4.24 (t, *J* = 5.7 Hz, 1H), 3.39 (t, *J* = 6.4 Hz, 2H), 3.33 (d, *J* = 12.1 Hz, 1H), 3.28 (s, 3H), 3.26 (s, 3H), 2.96 – 2.92 (m, 1H), 2.58 (s, 3H), 2.42 (s, 3H), 2.16 – 2.03 (m, 2H), 1.78 – 1.66 (m, 2H), 1.65 – 1.56 (m, 1H), 1.45 – 1.27 (m, 2H), 1.21 (s, 3H), 1.14 – 0.99 (m, 2H) ppm;

**$^{13}$C NMR** (101 MHz, $CDCl_3$) δ 213.83, 143.46, 137.85, 136.07, 133.11, 129.50 (2C), 128.37 (2C), 127.77 (2C), 127.73 (2C), 127.59, 118.99, 104.07, 73.17, 66.47, 54.07, 53.06, 52.49, 51.90, 48.53, 42.95, 41.23, 39.12, 34.02, 32.23, 31.31, 24.29, 23.43, 21.50 ppm;

**HRMS** (ESI): calculated for $[C_{32}H_{43}NO_5S+H]^+$ 570.2884, found 570.2885.

**Synthesis of compound S1**

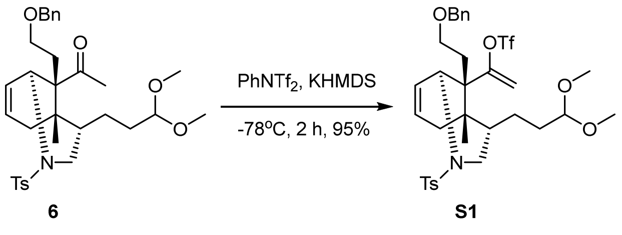


To a solution of compound **6** (1.71 g, 3.00 mmol, 1.0 equiv) and $PhNTf_2$ (1.52 g, 4.50 mmol, 1.5 equiv) in THF (20 ml) was added a solution of 0.5 M KHMDS (12.0 mL, 6.00 mmol, 2.0 equiv) in toluene at −78 °C. After being stirred for 2 h at that temperature, the reaction mixture was quenched with saturated aqueous $NH_4Cl$ (30 mL). The organic phase was separated and the aqueous phase was

extracted with EtOAc (3 × 30 mL). The combined organic phases were washed with brine, dried over anhydrous $Na_2SO_4$, filtered, and concentrated. The residue was purified by flash chromatography on silica gel (eluent: EtOAc/PE = 1/20 to 1/6) to give compound **S1** (2.00 g, yield: 95%) as a white foam.

**Rf** = 0.55 (silica, EtOAc/PE = 1:4);

**$^1$H NMR** (400 MHz, $CDCl_3$) δ 7.55 (d, *J* = 8.2 Hz, 2H), 7.42 – 7.30 (m, 5H), 7.22 (d, *J* = 8.1 Hz, 2H), 6.13 (d, *J* = 5.6 Hz, 1H), 5.77 (dt, *J* = 9.8, 3.4 Hz, 1H), 5.48 (d, *J* = 5.5 Hz, 1H), 4.91 – 4.83 (m, 1H), 4.74 (d, *J* = 6.4 Hz, 1H), 4.47 (d, *J* = 11.4 Hz, 1H), 4.41 (d, *J* = 11.4 Hz, 1H), 4.28 (dd, *J* = 6.5, 3.8 Hz, 1H), 3.64 – 3.49 (m, 2H), 3.39 (d, *J* = 11.9 Hz, 1H), 3.31 (s, 3H), 3.29 (s, 3H), 2.96 (dd, *J* = 11.7, 3.7 Hz, 1H), 2.41 (s, 3H), 2.16 – 2.05 (m, 1H), 1.91 – 1.75 (m, 2H), 1.70 – 1.58 (m, 4H), 1.42 – 1.30 (m, 1H), 1.14 – 1.10 (m, 1H), 1.10 (s, 3H) ppm;

**$^{13}$C NMR** (126 MHz, $CDCl_3$) δ 158.29, 143.57, 138.15, 136.13, 132.32, 129.62 (2C), 128.36 (2C), 127.81 (2C), 127.57, 127.40 (2C), 118.91, 118.17 (q, *J* = 319.4 Hz),106.76, 104.33, 73.36, 65.85, 53.21, 52.81, 52.23, 48.64, 47.67, 43.18, 41.71, 39.34, 32.84, 29.25, 24.38, 23.71, 21.52 ppm;

**$^{19}$F NMR** (376 MHz, $CDCl_3$) δ -75.33 ppm;

**HRMS** (ESI): calculated for $[C_{33}H_{42}F_3NO_8S+H]^+$ 702.2377 found 702.2376.

**Synthesis of compound 7**

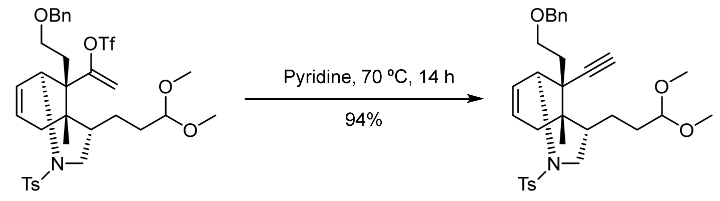


**S1** **7**

A solution of compound **S1** (701.8 mg, 1.00 mmol, 1.0 equiv) in pyridine (25 mL) was stirred for 14 h at 70 °C. The solvent was removed under reduced pressure and the residue was purified by flash chromatography on silica gel (eluent: EtOAc/PE = 1/5) to give the compound **7** (518.6 mg, yield: 94%) as a white foam.

**Rf** = 0.50 (silica, EtOAc/PE = 1:4);

**$^1$H NMR** (400 MHz,$CD_2Cl_2$) δ 7.64 (d, *J* = 8.2 Hz, 2H), 7.36 (d, *J* = 4.4 Hz, 4H), 7.33 – 7.26 (m, 1H), 7.25 (d, *J* = 8.0 Hz, 2H), 5.90 (dt, *J* = 9.8, 3.5 Hz, 1H), 5.05 – 4.98 (m, 1H), 4.54 (d, *J* = 6.1 Hz, 1H), 4.48 (s, 2H), 4.22 (t, *J* = 5.6 Hz, 1H), 3.86 – 3.70 (m, 2H), 3.34 (d, *J* = 12.5 Hz, 1H), 3.27 (s, 3H), 3.25 (s, 3H), 3.11 – 3.01 (m, 1H), 2.40 (s, 3H), 2.31 (s, 1H), 2.09 (dt, *J* = 19.7, 2.9 Hz, 1H), 1.91 – 1.78 (m,

3H), 1.77 – 1.72 (m, 1H), 1.68 – 1.60 (m, 1H), 1.60 – 1.50 (m, 1H), 1.35 – 1.23 (m, 1H), 1.15 – 1.07 (m, 1H), 1.06 (s, 3H) ppm;

**$^{13}$C NMR** (101 MHz, $CD_2Cl_2$) δ 143.54, 139.14, 137.71, 133.69, 129.79 (2C), 128.63 (2C), 127.91 (2C), 127.75, 127.63 (2C), 119.25, 104.86, 87.25, 74.61, 73.33, 67.69, 54.88, 52.96, 52.50, 49.33, 42.15, 41.66, 41.53, 37.28, 33.31, 33.22, 25.45, 23.40, 21.61 ppm;

**HRMS** (ESI): calculated for $[C_{32}H_{41}NO_5S+H]^+$ 552.2778, found 552.2779.

**Synthesis of compound 3**

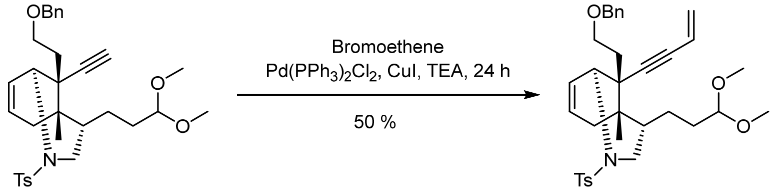


To a solution of $Pd(PPh_3)_2Cl_2$ (35.0 mg, 0.050 mmol, 0.10 equiv ), CuI (104.7 mg, 0.55 mmol, 1.1 equiv) and vinyl bromide (12.5 mL, 25 equiv, 1 M in THF) in THF (10 mL) was added TEA (0.70 mL, 5.0 mmol, 10 equiv). The color of mixture changed to black from yellow. A solution of compound **7** (276 mg, 0.50 mmol, 1.0 equiv) in THF (6 mL) was added dropwise over 0.5 h and the reaction mixture was stirred for 24 h at the room temperature. The reaction mixture was poured into saturated aqueous $NH_4Cl$ (100 mL) and ethyl acetate (100 mL), and extracted with EtOAc (3 × 100 mL). The extract was washed with brine, dried over anhydrous $Na_2SO_4$ and concentrated under reduced pressure. The residue was purified by flash chromatography (eluent: EtOAc/PE = 1/7) to give the compound **3** (144.4 mg, yield: 50%) as a colorless foam.

**Rf** = 0.55 (silica, EtOAc/PE = 1:4);

**$^1$H NMR** (400 MHz, $CD_2Cl_2$) δ 7.70 – 7.64 (m, 2H), 7.39 – 7.26 (m, 5H), 7.26 – 7.21 (m, 2H), 5.97 (dt, *J* = 9.8, 3.5 Hz, 1H), 5.79 (dd, *J* = 17.6, 11.0 Hz, 1H), 5.58 (dd, *J* = 17.6, 2.4 Hz, 1H), 5.46 (dd, *J* = 11.0, 2.4 Hz, 1H), 5.19 – 5.13 (m, 1H), 4.54 (d, *J* = 6.1 Hz, 1H), 4.49 (s, 2H), 4.11 (t, *J* = 5.8 Hz, 1H), 3.82 – 3.70 (m, 2H), 3.29 – 3.24 (m, 1H), 3.24 (s, 3H), 3.22 (s, 3H), 3.12 (ddd, *J* = 12.7, 4.1, 1.9 Hz, 1H), 2.40 (s, 3H), 2.12 (dt, *J* = 19.7, 2.9 Hz, 1H), 1.92 – 1.80 (m, 2H), 1.79 – 1.73 (m, 1H), 1.73 – 1.65 (m, 1H), 1.65 – 1.56 (m, 1H), 1.51 – 1.40 (m, 1H), 1.31 – 1.20 (m, 1H), 1.13 – 1.06 (m, 1H), 1.04 (s, 3H) ppm;

$^{13}$**C NMR** (101 MHz, $CD_2Cl_2$) δ 143.50, 139.18, 137.80, 133.66, 129.85 (2C), 128.65 (2C), 127.94 (2C), 127.79 (2C), 127.76, 126.09, 119.66, 117.92, 104.99, 94.11, 84.98, 73.35, 67.83, 54.86, 53.02, 52.39, 49.32, 42.56, 41.65, 41.45, 37.73, 33.28, 33.24, 25.59, 23.57, 21.63 ppm;

**HRMS** (ESI): calculated for $[C_{34}H_{43}NO_5S+H]^+$ 578.2935, found 578.2934.

**Synthesis of compound 9**

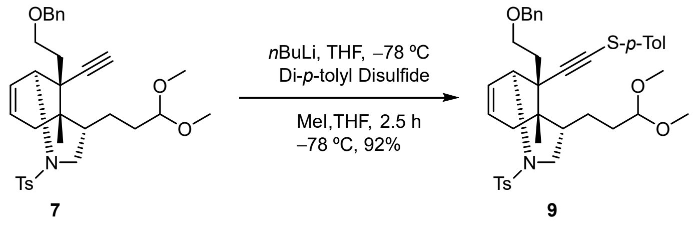


To a solution of compound **7** (276 mg, 0.50 mmol, 1.0 equiv) in THF (5 mL) at −78 °C was added *n*-BuLi (0.38 mL, 1.6 M in Hexane, 1.1 equiv) dropwise, and the mixture was stirred for 1 h at that temperature. To a solution of *p*-tolyl disulfide (184.8 mg, 0.75 mmol, 1.5 equiv) at room temperature, iodomethane (0.047 mL, 0.75 mmol, 1.5 equiv) was added, and the mixture was stirred for 1 h. The latter mixture was added dropwise to the former mixture at −78 °C, and the reaction mixture was stirred for 2.5 h at that temperature. The reaction was quenched with saturated aqueous $NH_4Cl$ (10 mL), and the organic phase was separated and the aqueous phase was extracted with EtOAc (3 × 10 mL). The combined organic phases were washed with brine, dried over anhydrous $Na_2SO_4$, filtered, and concentrated under reduced pressure. The residue was purified by flash chromatography (eluent: EtOAc/PE = 1/5) to give the compound **9** (310 mg, yield: 92%) as a yellow foam.

**Rf** = 0.53 (silica, EtOAc/PE = 1:2);

$^{1}$**H NMR** (400 MHz, $CD_2Cl_2$) δ 7.70 – 7.60 (m, 2H), 7.45 (d, $J$ = 8.3 Hz, 2H), 7.39 – 7.27 (m, 5H), 7.25 (d, $J$ = 8.1 Hz, 2H), 7.19 (d, $J$ = 8.0 Hz, 2H), 5.94 (dt, $J$ = 9.7, 3.4 Hz, 1H), 5.12 – 5.04 (m, 1H), 4.67 (d, $J$ = 6.1 Hz, 1H), 4.50 (d, $J$ = 11.8 Hz, 1H), 4.47 (d, $J$ = 11.8 Hz, 1H), 4.00 (dd, $J$ = 6.4, 5.1 Hz, 1H), 3.89 – 3.73 (m, 2H), 3.37 (d, $J$ = 12.5 Hz, 1H), 3.19 (s, 3H), 3.17 (s, 3H), 3.09 (ddd, $J$ = 12.5, 4.2, 1.9 Hz, 1H), 2.40 (s, 3H), 2.34 (s, 3H), 2.17 – 2.08 (m, 1H), 1.93 – 1.70 (m, 4H), 1.67 – 1.55 (m, 1H), 1.54 – 1.43 (m, 1H), 1.33 – 1.20 (m, 1H), 1.18 – 1.13 (m, 1H), 1.12 (s, 3H) ppm;

$^{13}$**C NMR** (101 MHz, $CD_2Cl_2$) δ 143.63, 139.09, 137.52, 136.71, 133.72, 130.37 (2C), 129.91 (2C), 129.68 (2C), 128.65 (2C), 127.90 (2C), 127.77, 127.71 (2C), 126.78, 119.27, 105.08, 102.00, 73.42,

71.30, 67.86, 55.04, 53.32, 52.58, 49.23, 43.91, 41.67, 41.54, 37.87, 33.39, 33.37, 25.62, 23.66, 21.63, 21.07 ppm;

**HRMS** (ESI): calculated for $[C_{39}H_{47}NO_5S_2+H]^+$ 674.2968, found 674.2970.

**Synthesis of compound 11**

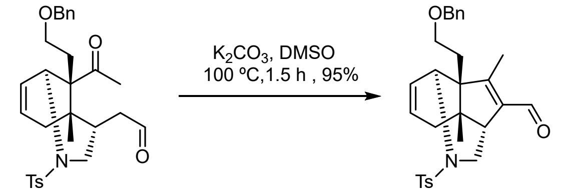


**5** **11**

A mixture of compound **5** (51.0 mg, 0.10 mmol, 1.0 equiv), DMSO (3 mL) and $K_2CO_3$ (55.3 mg, 0.40 mmol, 4.0 equiv) was stirred for 1.5 h at 100 °C. The reaction was quenched with saturated aqueous $NH_4Cl$ (5 mL), and the mixture was extracted with EtOAc (3 × 10 mL). The combined organic phases were washed with brine, dried over anhydrous $Na_2SO_4$, filtered, and concentrated under reduced pressure. The residue was purified by flash chromatography (eluent: EtOAc/PE = 1/4) to give the compound **11** (46.7 mg, yield: 95%) as a white foam.

**Rf** = 0.60 (silica, EtOAc/PE = 1:2);

**$^1$H NMR** (400 MHz, $CDCl_3$) δ 9.70 (s, 1H), 7.48 (d, $J$ = 8.3 Hz, 2H), 7.39 – 7.29 (m, 5H), 7.18 (d, $J$ = 8.1 Hz, 2H), 5.91 (dt, $J$ = 9.6, 3.5 Hz, 1H), 5.79 – 5.71 (m, 1H), 4.50 (s, 2H), 4.18 (d, $J$ = 6.4 Hz, 1H), 3.66 – 3.56 (m, 1H), 3.54 – 3.45 (m, 1H), 3.18 (dd, $J$ = 12.5, 3.6 Hz, 1H), 2.93 (dd, $J$ = 12.5, 1.2 Hz, 1H), 2.51 (d, $J$ = 3.4 Hz, 1H), 2.38 (s, 3H), 2.26 – 2.17 (m, 1H), 2.07 – 1.98 (m, 1H), 1.96 (s, 3H), 1.88 – 1.80 (m, 1H), 1.67 – 1.59 (m, 1H), 0.84 (s, 3H) ppm;

**$^{13}$C NMR** (101 MHz, $CDCl_3$) δ 186.62, 164.71, 142.78, 141.01, 138.21, 137.92, 130.29, 129.38 (2C), 128.45 (2C), 127.76 (2C), 127.73, 126.72 (2C), 122.51, 73.35, 66.76, 54.01, 49.34, 47.91, 44.52, 38.77, 33.54, 28.30, 24.52, 21.42, 12.27 ppm;

**HRMS** (ESI): calculated for $[C_{29}H_{33}NO_4S+H]^+$ 492.2203, found 492.2201.

**Synthesis of compound S2**

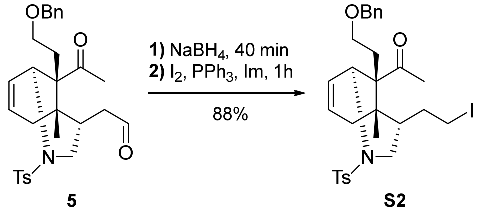

Compound **5** (1.02 g, 2.00 mmol, 1.0 equiv) was dissolved in a mixture of THF (25 mL) and MeOH (25 mL). $NaBH_4$ (76.0 mg, 2.00 mmol, 1.0 equiv) was divided into two parts and added over 15 min, the resulting mixture was stirred at 0 °C for 40 min, then quenched with 2 N HCl (10 mL) and brine (20 mL). The organic phase was separated and the aqueous phase was extracted with EtOAc (4 × 80 mL). The combined organic phases were washed with brine, dried over anhydrous $Na_2SO_4$, filtered, and concentrated. The resulting residue was used directly for the next step without further purification.

To a solution of the aforementioned crude product, $PPh_3$ (681 mg, 2.60 mmol, 1.3 equiv) and imidazole (177 mg, 2.60 mmol, 1.3 equiv) in THF (20 mL), $I_2$ (660 mg, 2.60 mmol, 1.3 equiv) was added in one portion at 0 °C. The ice bath was removed and the brown reaction mixture was stirred at room temperature for 1 h. The reaction was quenched with saturated aqueous $NaHCO_3$ (50 mL) and saturated aqueous $Na_2S_2O_3$ (20 mL) and the layers were separated. The aqueous phase was extracted with EtOAc (3 × 60 mL). The combined organic phases were washed with brine, dried over anhydrous $Na_2SO_4$, filtered, and concentrated under reduced pressure. The residue was purified by flash chromatography on silica gel (eluent: EtOAc/PE, 1/9) to give the corresponding iodoalkane **S2** (1.09 g, yield: 88%, over two steps) as a colorless gum.

**Rf** = 0.80 (silica, EtOAc/PE = 1:2);

**$^1$H NMR** (400 MHz, $CDCl_3$) δ 7.66 – 7.61 (m, 2H), 7.41 – 7.30 (m, 5H), 7.27 (d, *J* = 8.5 Hz, 2H), 5.80 (dt, *J* = 9.2, 3.4 Hz, 1H), 5.07 (d, *J* = 6.3 Hz, 1H), 5.05 – 4.99 (m, 1H), 4.45 (d, *J* = 11.7 Hz, 1H), 4.41 (d, *J* = 11.7 Hz, 1H), 3.45 – 3.34 (m, 2H), 3.31 – 3.23 (m, 2H), 3.01 – 2.91 (m, 2H), 2.57 (s, 3H), 2.43 (s, 3H), 2.14 (dt, *J* = 19.9, 2.8 Hz, 1H), 2.08 – 1.99 (m, 1H), 1.91 – 1.77 (m, 2H), 1.74 – 1.65 (m, 1H), 1.55 – 1.45 (m, 1H), 1.37 – 1.31 (m, 1H), 1.23 (s, 3H) ppm;

**$^{13}$C NMR** (101 MHz, $CDCl_3$) δ 214.09, 143.69, 137.78, 135.83, 133.22, 129.61 (2C), 128.38 (2C), 127.78 (2C), 127.66 (2C), 127.63, 118.82, 73.20, 66.46, 54.39, 52.70, 48.41, 42.87, 40.75, 38.77, 33.97, 32.35, 31.37, 23.14, 21.54, 7.26 ppm;

**HRMS** (ESI): calculated for $[C_{29}H_{36}INO_4S+H]^+$ 622.1482, found 622.1485.

## Synthesis of compound 13

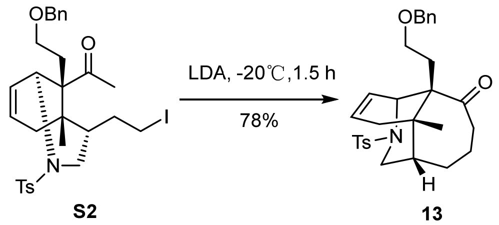


To a cooled (−20 °C) solution of compound **S2** (1.24 g, 2.00 mmol, 1.0 equiv) in THF (55 mL) was added LDA (3.5 mL, 2 M in THF, 3.5 equiv) dropwise over 1 h. The mixture was stirred for another 0.5 h at that temperature and quenched with saturated aqueous $NH_4Cl$ (25 mL), and the mixture was extracted with EtOAc (3 × 30 mL). The combined organic phases were washed with brine, dried over anhydrous $Na_2SO_4$, filtered, and concentrated under reduced pressure. The residue was purified by flash chromatography (eluent: MeOH/DCM = 1/50) to give the compound **13** (770 mg, yield: 78%) as a white foam.

**Rf** = 0.55 (silica, EtOAc/PE = 1:4);

**$^1$H NMR** (400 MHz, $CDCl_3$) δ 7.67 – 7.61 (m, 2H), 7.42 – 7.29 (m, 5H), 7.28 (d, *J* = 7.2 Hz, 2H), 5.89 (dt, *J* = 9.7, 3.5 Hz, 1H), 5.13 – 5.03 (m, 1H), 4.73 (d, *J* = 6.1 Hz, 1H), 4.45 (s, 2H), 3.79 – 3.68 (m, 1H), 3.58 – 3.52 (m, 1H), 3.49 (d, *J* = 12.5 Hz, 1H), 3.24 – 3.17 (m, 1H), 2.79 (td, *J* = 12.9, 3.7 Hz, 1H), 2.70 – 2.62 (m, 1H), 2.43 (s, 3H), 2.24 (dt, *J* = 19.9, 2.8 Hz, 1H), 2.16 – 2.03 (m, 1H), 2.02 – 1.88 (m, 2H), 1.82 – 1.61 (m, 5H), 1.28 (s, 3H) ppm;

**$^{13}$C NMR** (101 MHz, $CDCl_3$) δ 214.13, 143.32, 138.32, 136.24, 133.42, 129.47 (2C), 128.30 (2C), 127.70 (2C), 127.51 (2C), 127.48, 118.61, 73.04, 67.74, 56.16, 56.05, 45.33, 45.30, 43.95, 43.74, 35.93, 32.55, 32.37, 24.46, 21.53, 20.36 ppm;

**HRMS** (ESI): calculated for $[C_{29}H_{35}NO_4S+H]^+$ 494.2360, found 494.2360.

# Supplementary Note 2

## Asymmetric Total Synthesis of (+)–Caldaphnidine J

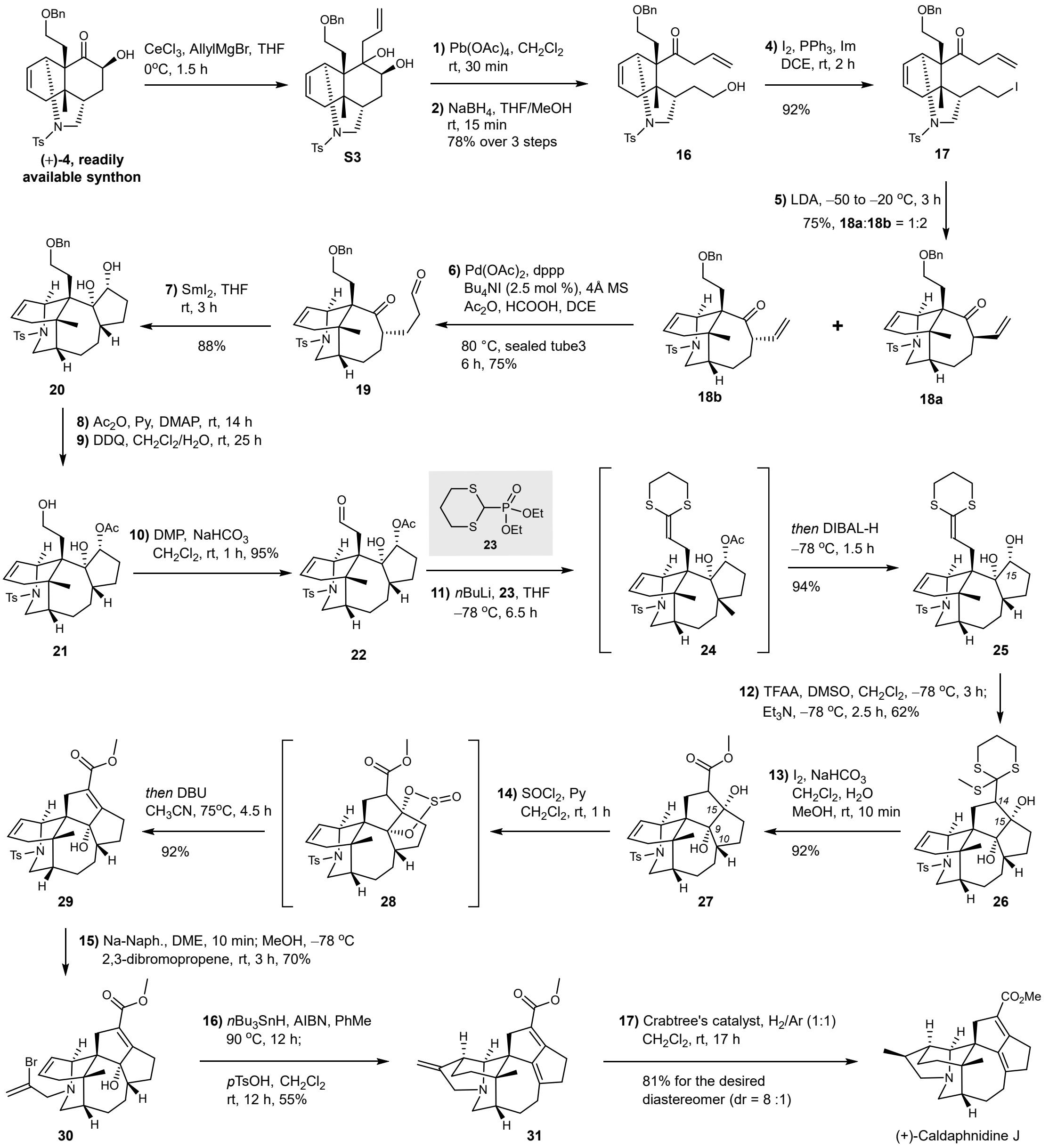


**Supplementary Figure 2.** Synthetic route of (+)–Caldaphnidine J

## Synthesis of compound S3

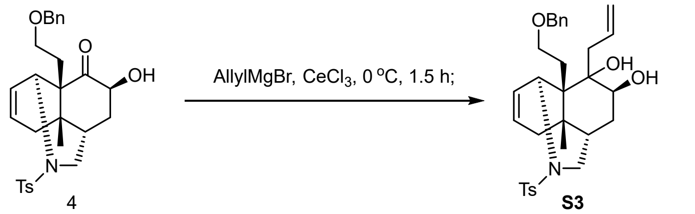


In a glovebox, a flask was charged with dry $CeCl_3$ (7.39 g, 30.0 mmol, 6.0 equiv) and then flushed with

argon, and dry THF (75 mL) was added. After being stirred for 2 h at room temperature, the reaction mixture was cooled to 0 °C. Allyl magnesium bromide (30.0 mL, 30.0 mmol, 1.0 M in THF, 6.0 equiv) was added dropwise to this stirred suspension at 0 °C. The resulting mixture was stirred at the same temperature for 1.5 h providing a yellow-orange cloudy solution of organocerium reagent, then a solution of ketone **4**[1,2] (2.48 g, 5.00 mmol, 1.0 equiv) in THF (10 mL) was added over 20 min. After being stirred for 1.5 h at 0 °C, 2 N HCl (40 mL) was added. THF was removed under reduced pressure, and then the aqueous phase was extracted with EtOAc (3 × 100 mL). The combined organic phases were washed with brine, dried over anhydrous $Na_2SO_4$, filtered, and concentrated under reduced pressure. The crude product (2.56 g) was used directly for the next step without further purification.

A small amount of material was purified by flash chromatography on silica gel (eluent: EtOAc/PE, 3/10) to give compound **S3** (single isomer) as a colorless foam.

**Rf** = 0.65 (silica, EtOAc/PE = 1:2); **$[\alpha]_D^{20}$** = +883.2 (*c* 0.4, $CHCl_3$);

**$^1$H NMR** (400 MHz, $CDCl_3$) δ 7.61 (d, *J* = 8.3 Hz, 2H), 7.37 – 7.30 (m, 4H), 7.30 – 7.24 (m, 3H), 6.28 – 6.13 (m, 1H), 5.69 (dt, *J* = 9.7, 3.4 Hz, 1H), 5.34 (dt, *J* = 17.2, 2.0 Hz, 1H), 5.17 (ddd, *J* = 10.0, 2.3, 1.2 Hz, 1H), 4.98 – 4.88 (m, 1H), 4.79 – 4.69 (m, 1H), 4.50 (d, *J* = 11.9 Hz, 1H), 4.46 (d, *J* = 11.9 Hz, 1H), 4.34 (d, *J* = 6.4 Hz, 1H), 3.78 – 3.71 (m, 1H), 3.61 – 3.55 (m, 1H), 3.55 (s, 1H), 3.50 (dd, *J* = 11.6, 1.9 Hz, 1H), 3.00 (ddd, *J* = 11.7, 3.4, 2.0 Hz, 1H), 2.84 (dd, *J* = 13.1, 9.5 Hz, 1H), 2.55 – 2.44 (m, 2H), 2.41 (s, 3H), 2.32 (d, *J* = 4.8 Hz, 1H), 2.11 (dt, *J* = 19.9, 2.9 Hz, 1H), 1.94 – 1.77 (m, 2H), 1.71 (ddd, *J* = 20.0, 3.7, 2.0 Hz, 1H), 1.54 – 1.44 (m, 2H), 1.20 (s, 3H).

**$^{13}$C NMR** (101 MHz, $CDCl_3$) δ 143.47, 138.44, 136.69, 136.46, 132.17, 129.64(2C), 128.38(2C), 127.66(2C), 127.54, 127.42(2C), 119.52, 118.51, 77.54, 72.92, 71.72, 68.86, 52.85, 48.36, 44.74, 42.82, 42.75, 41.67, 35.60, 33.47, 29.60, 25.74, 21.58.

**HRMS** (ESI): calculated for $[C_{31}H_{39}NO_5S+Na]^+$ 560.2441, found 560.2442.

## Synthesis of compound 16

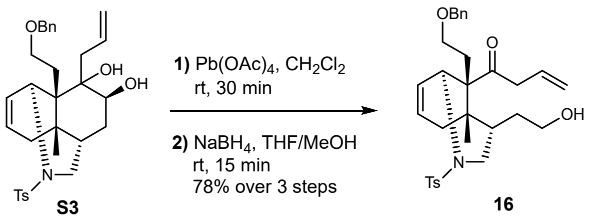

To a solution of the aforementioned compound (crude, 2.56 g) in DCM (100 mL) was added $Pb(OAc)_4$ (3.33 g, 7.50 mmol, 1.5 equiv). After being stirred for 30 min at room temperature, saturated aqueous $NaHCO_3$ (150 mL) was added. The resulting mixture was stirred for 1 h at room temperature. The reaction mixture was filtered through Celite (eluent: EtOAc). The organic phase was separated and the aqueous phase was extracted with EtOAc (3 × 100 mL). The combined organic phases were washed with brine, dried over anhydrous $Na_2SO_4$, filtered, and concentrated under reduced pressure. The residue was used directly for the next step without further purification.

To a solution of the aforementioned crude mixture in THF (50 mL) and MeOH (50 mL) was added $NaBH_4$ (380 mg, 10.0 mmol, 2.0 equiv) in one portion, the resulting mixture was stirred at room temperature for 15 min, and then quenched with 2 N HCl (20 mL) and stirred for 0.5 h. Volatiles were removed under reduced pressure, and the aqueous phase was extracted with EtOAc (4 × 80 mL). The combined organic phases were washed with brine, dried over anhydrous $Na_2SO_4$, filtered, and concentrated under reduced pressure. The resulting residue was purified by flash column chromatography (eluent: EtOAc/PE = 1/4 to 1/2) to give the compound **16** (2.09 g, yield: 78%, over 3 steps) as a white foam.

**Rf** = 0.20 (silica, EtOAc/PE = 1/4);

**$^1$H NMR** (400 MHz, CDCl3) δ 7.63 (d, J = 7.3 Hz, 2H), 7.42 – 7.31 (m, 5H), 7.26 (d, J = 7.0 Hz, 2H), 5.91 (dt, J = 16.9, 8.5 Hz, 1H), 5.78 (d, J = 9.3 Hz, 1H), 5.16 – 4.93 (m, 4H), 4.44 (d, J = 12.0 Hz, 1H), 4.40 (d, J = 11.6 Hz, 1H), 4.04 (dd, J = 19.0, 6.8 Hz, 1H), 3.73 (dd, J = 19.0, 6.4 Hz, 1H), 3.66 – 3.30 (m, 5H), 2.96 (d, J = 10.9 Hz, 1H), 2.42 (s, 3H), 2.05 (d, J = 12.4 Hz, 3H), 1.83 – 1.65 (m, 2H), 1.65 – 1.53 (m, 1H), 1.44 – 1.33 (m, 1H), 1.21 (s, 3H) ppm;

**$^{13}$C NMR** (101 MHz, CDCl3) δ 214.13, 143.51, 137.72, 135.92, 133.15, 132.06, 129.51 (2C), 128.34 (2C), 127.80 (2C), 127.62 (2C), 127.58, 118.67, 117.67, 73.16, 66.39, 61.60, 60.31, 54.33, 52.08, 46.52, 44.50, 42.82, 41.92, 38.93, 34.14, 32.45, 23.29, 21.45, 20.92, 20.57, 14.06 ppm;

**HRMS** (ESI): calculated for $[C_{31}H_{39}NO_5S+H]^+$ 538.2622, found 538.2623.

**Synthesis of compound 17**

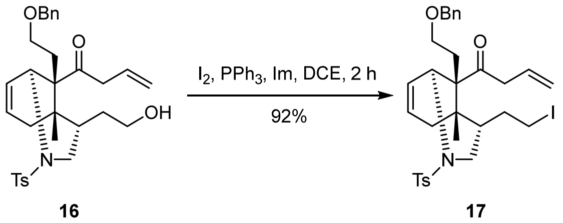

To a solution of compound **16** (1.08 g, 2.00 mmol, 1.0 equiv), $PPh_3$ (682 mg, 2.60 mmol, 1.3 equiv) and imidazole (272 mg, 4.00 mmol, 2.0 equiv) in DCE (20 mL) was added $I_2$ (660 mg, 2.60 mmol, 1.3 equiv) in one portion at room temperature. The mixture was stirred for 2 h and quenched with saturated aqueous $NaHCO_3$ (20 mL). The mixture was extracted with DCM (3 × 40 mL). The combined organic phases were washed with brine, dried over anhydrous $Na_2SO_4$, filtered, and concentrated under reduced pressure. The residue was purified by flash column chromatography (eluent: EtOAc/PE = 0/1 to 1/10) to give compound **17** (1.19 g, yield: 92%) as a yellow oil.

**Rf** = 0.75 (silica, EtOAc/PE = 1/2);

**$^1$H NMR** (400 MHz, $CDCl_3$) δ 7.64 (d, *J* = 8.3 Hz, 2H), 7.43 – 7.28 (m, 5H), 7.28 (d, *J* = 8.0 Hz, 2H), 5.91 (ddt, *J* = 17.0, 10.3, 6.7 Hz, 1H), 5.80 (dt, *J* = 9.6, 3.4 Hz, 1H), 5.18 – 4.98 (m, 4H), 4.44 (d, *J* = 11.7 Hz, 1H), 4.40 (d, *J* = 11.7 Hz, 1H), 4.01 (ddt, *J* = 19.0, 6.7, 1.3 Hz, 1H), 3.67 (ddt, *J* = 19.0, 6.7, 1.4 Hz, 1H), 3.43 – 3.30 (m, 2H), 3.32 – 3.20 (m, 2H), 3.02 – 2.89 (m, 2H), 2.43 (s, 3H), 2.14 (dt, *J* = 19.9, 2.8 Hz, 1H), 2.09 – 1.98 (m, 1H), 1.88 – 1.76 (m, 2H), 1.77 – 1.65 (m, 1H), 1.52 – 1.39 (m, 1H), 1.38 – 1.29 (m, 1H), 1.22 (s, 3H) ppm;

**$^{13}$C NMR** (101 MHz, $CDCl_3$) δ 213.87, 143.76, 137.77, 135.86, 133.14, 131.92, 129.64 (2C), 128.43 (2C), 127.87 (2C), 127.69 (3C), 118.87, 117.93, 73.28, 66.46, 54.55, 52.19, 48.46, 46.49, 42.91, 40.81, 38.97, 34.16, 32.48, 23.15, 21.55, 7.07 ppm;

**HRMS** (ESI): calculated for $[C_{31}H_{38}INO_4S+H]^+$ 648.1639, found 648.1636.

**Syntheses of compound 18a and 18b**

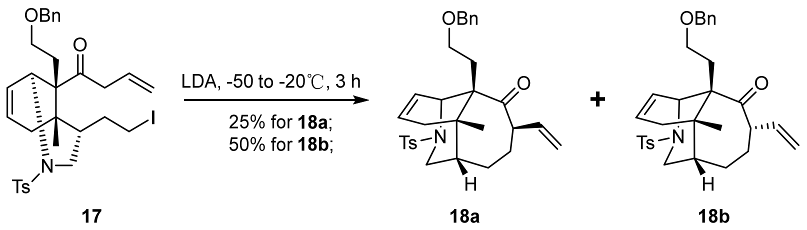

To a solution of compound **17** (2.59 g, 4.00 mmol, 1.0 equiv) in THF (130 mL) at −50 °C was added LDA (3.4 mL, 2 M in THF, 1.7 equiv) dropwise over 1 h. The mixture was warmed up to −20 °C stirred for 2 h and quenched with saturated aqueous $NH_4Cl$ (30 mL). Volatiles were removed under reduced pressure, and the mixture was extracted with DCM (3 × 50 mL). The combined organic phases were washed with brine, dried over anhydrous $Na_2SO_4$, filtered, and concentrated under reduced pressure. The residue was purified by flash column chromatography on silica gel (eluent: EtOAc/PE, 0/1 to 1/10 to 1/4) to give compound **18a** (520 mg, yield: 25%) and **18b** (1.04 g, yield: 50%), both as a white foam.

**Data of 18a**

**Rf** = 0.50 (silica, EtOAc/PE = 1:4);

$[\alpha]_D^{20}$ = +98.4 (*c* 0.3, $CHCl_3$);

**$^1$H NMR** (400 MHz, $CDCl_3$) δ 7.64 (d, *J* = 8.2 Hz, 2H), 7.42 – 7.28 (m, 5H), 7.26 (d, *J* = 7.9 Hz, 2H), 6.16 – 6.03 (m, 1H), 5.65 (dt, *J* = 9.8, 3.4 Hz, 1H), 5.09 – 4.99 (m, 1H), 4.97 (d, *J* = 3.2 Hz, 1H), 4.94 (s, 1H), 4.82 (d, *J* = 6.2 Hz, 1H), 4.42 (d, *J* = 11.9 Hz, 1H), 4.37 (d, *J* = 11.9 Hz, 1H), 4.32 – 4.23 (m, 1H), 3.71 (d, *J* = 11.8 Hz, 1H), 3.28 (dd, *J* = 6.9, 5.8 Hz, 2H), 3.12 (ddd, *J* = 11.8, 4.0, 1.9 Hz, 1H), 2.62 (dt, *J* = 14.2, 7.3 Hz, 1H), 2.55 – 2.44 (m, 1H), 2.43 (s, 3H), 2.02 (dt, *J* = 19.7, 3.0 Hz, 1H), 1.90 (ddd, *J* = 19.7, 3.7, 1.9 Hz, 1H), 1.84 – 1.64 (m, 2H), 1.55 – 1.36 (m, 3H), 0.89 (s, 3H) ppm;

**$^{13}$C NMR** (101 MHz, $CDCl_3$) δ 211.86, 143.46, 139.22, 138.20, 136.69, 130.90, 129.61 (2C), 128.35 (2C), 128.03 (2C), 127.48, 127.44 (2C), 120.32, 114.17, 73.26, 66.80, 55.57, 52.57, 51.28, 44.44, 41.87, 40.04, 34.48, 30.70, 27.23, 27.01, 23.00, 21.56 ppm;

**HRMS** (ESI): calculated for $[C_{31}H_{37}NO_5S+H]^+$ 520.2516, found 520.2517.

**Data of 18b**

**Rf** = 0.40 (silica, EtOAc/PE = 1:4);

$[\alpha]_D^{20}$ = +85.0 (*c* 0.2, $CHCl_3$);

**$^1$H NMR** (400 MHz, $CDCl_3$) δ 7.63 (d, *J* = 8.3 Hz, 2H), 7.35 – 7.26 (m, 5H), 7.25 (d, *J* = 7.8 Hz, 2H), 6.05 – 5.96 (m, 1H), 5.93 (dt, *J* = 9.8, 3.1 Hz, 1H), 5.27 – 5.19 (m, 1H), 5.02 (dd, *J* = 10.3, 1.6 Hz, 1H), 4.84 (d, *J* = 17.3 Hz, 1H), 4.79 (d, *J* = 6.1 Hz, 1H), 4.42 (s, 2H), 3.66 (td, *J* = 8.9, 6.1 Hz, 1H), 3.50 (td, *J* = 9.2, 6.3 Hz, 1H), 3.43 (td, *J* = 8.7, 7.9, 4.4 Hz, 1H), 3.36 (d, *J* = 12.4 Hz, 1H), 3.25 (dd, *J* = 11.8, 4.5 Hz, 1H), 2.41 (s, 3H), 2.25 (dt, *J* = 19.8, 2.9 Hz, 1H), 2.22 – 2.13 (m, 1H), 1.94 (ddd, *J* = 19.9, 4.0, 1.9 Hz, 1H), 1.78 – 1.63 (m, 5H), 1.52 – 1.44 (m, 1H), 1.34 (s, 3H) ppm;

$^{13}$**C NMR** (101 MHz, $CDCl_3$) δ 212.83, 143.30, 138.34, 138.32, 136.45, 133.23, 129.41 (2C), 128.31 (2C), 127.73 (2C), 127.62 (2C), 127.50, 119.03, 114.00, 73.12, 67.56, 56.63, 56.54, 56.34, 45.08, 44.28, 43.89, 36.19, 32.57, 32.29, 27.80, 24.84, 21.53 ppm;

**HRMS** (ESI): calculated for $[C_{31}H_{37}NO_5S+H]^+$ 520.2516, found 520.2517.

**Synthesis of compound 17**

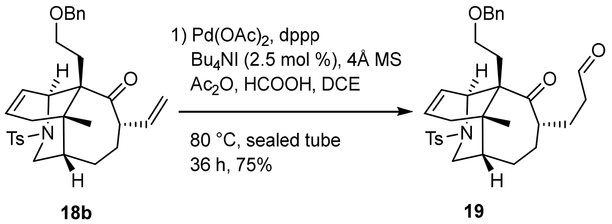


To a mixture of $Pd(OAc)_2$ (5.6 mg, 0.025 mmol, 0.10 equiv), dppp (20.6 mg, 0.050 mmol, 0.20 equiv), $Bu_4NI$ (2.3 mg, $6.3\times10^{-3}$ mmol, 0.025 equiv), 4 Å molecular sieves (20 mg), and 1,2-dichloroethane (DCE) (0.50 mL) in a vial (4.0 mL) were added **18b** (130 mg, 0.25 mmol, 1.0 equiv) dissolved in DCE (0.50 mL), $Ac_2O$ (118 µL, 1.25 mmol, 5.0 equiv), and HCOOH (61.3 µL, 1.63 mmol, 6.5 equiv ) successively via syringe. The vial was purged with argon and tightly sealed with a septum cap. The reaction mixture was stirred at 80 °C for 36 h and cooled to room temperature, then purified by flash chromatography (eluent:EtOAc/PE = 1/8 to 1/2) to give compound **19** (103 mg, 75% yield) as a colorless oil.

**Rf** = 0.67 (silica, EtOAc/PE = 1/1);

$[\alpha]_D^{20}$ = +126.5 (*c* 2.0, $CHCl_3$);

$^1$**H NMR** (400 MHz, $CDCl_3$) δ 9.68 (s, 1H), 7.61 (d, *J* = 8.4 Hz, 2H), 7.34 – 7.24 (m, 7H), 5.89 (dt, *J* = 9.8, 3.5 Hz, 1H), 5.16 – 5.06 (m, 1H), 4.80 (d, *J* = 6.1 Hz, 1H), 4.45 (s, 2H), 3.63 (td, *J* = 9.4, 5.3 Hz, 1H), 3.48 (td, *J* = 9.4, 5.7 Hz, 1H), 3.38 (d, *J* = 12.4 Hz, 1H), 3.24 – 3.14 (m, 1H), 2.92 – 2.82 (m, 1H), 2.41 (s, 3H), 2.37 – 2.12 (m, 3H), 2.05 – 1.96 (m, 1H), 1.96 – 1.88 (m, 1H), 1.78 – 1.60 (m, 5H), 1.57 – 1.41 (m, 3H), 1.31 (s, 3H) ppm;

$^{13}$**C NMR** (101 MHz, $CDCl_3$) δ 213.27, 202.63, 143.32, 138.28, 136.39, 133.39, 129.46 (2C), 128.37 (2C), 127.71 (2C), 127.58, 127.53 (2C), 118.67, 73.06, 67.25, 56.41, 56.11, 50.74, 45.02, 44.24, 43.98, 41.79, 36.22, 32.48, 32.33, 27.89, 24.90, 24.43, 21.54 ppm;

**HRMS (ESI)**: calculated for $[C_{32}H_{39}NO_5S+H]^+$ 550.2622, found 550.2624.

## Synthesis of compound 20

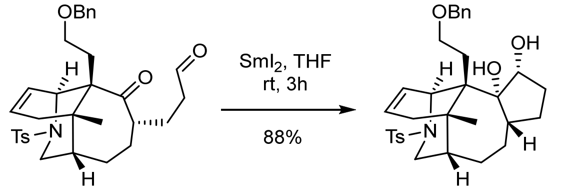


19 20

A solution of compound **19** (1.65 g, 3.00 mmol, 1.0 equiv) in THF (20 mL) was added dropwise to the solution of $SmI_2$ in THF (180 mL, 0.1 M in THF, 6.0 equiv) over 2.5 h at room temperature. The reaction was stirred for 0.5 h and quenched slowly with saturated aqueous $NH_4Cl$ (120 mL). Volatiles were removed under reduced pressure, and the aqueous phase was extracted with EtOAc (4 × 80 mL). The combined organic phases were washed with brine, dried over anhydrous $Na_2SO_4$, filtered, and concentrated under reduced pressure. The residue was purified by flash chromatography (eluent: EtOAc/PE = 0/1 to 1/8) to give compound **20** (1.45 g, yield: 88%) as a yellow oil.

**Rf** = 0.66 (silica, EtOAc/PE = 2:3); **$[\alpha]_D^{20}$** = +81.6 (*c* 2.0, $CHCl_3$);

**$^1$H NMR** (400 MHz, $CDCl_3$) δ 7.65 (d, *J* = 8.3 Hz, 2H), 7.34 – 7.23 (m, 7H), 5.73 (dt, *J* = 9.2, 3.3 Hz, 1H), 4.53 (q, *J* = 6.1 Hz, 2H), 4.49 (d, *J* = 11.6 Hz, 1H), 4.41 (d, *J* = 11.6 Hz, 1H), 4.20 – 4.07 (m, 2H), 3.61 (d, *J* = 1.8 Hz, 1H), 3.57 (d, *J* = 11.8 Hz, 1H), 3.44 (td, *J* = 8.6, 6.9 Hz, 1H), 3.30 (ddd, *J* = 11.9, 5.2, 2.1 Hz, 1H), 2.66 (d, *J* = 9.9 Hz, 1H), 2.43 (s, 3H), 2.31 – 2.08 (m, 5H), 1.88 – 1.69 (m, 5H), 1.60 (q, *J* = 4.2 Hz, 1H), 1.53 – 1.37 (m, 2H), 1.11 (s, 3H) ppm;

**$^{13}$C NMR** (101 MHz, $CDCl_3$) δ 143.77, 138.52, 135.69, 132.69, 129.75 (2C), 128.22 (2C), 127.71 (2C), 127.36 (3C), 119.53, 85.91, 80.64, 72.99, 69.45, 52.68, 49.94, 48.67, 47.32, 45.56, 45.35, 40.43, 35.45, 33.07, 31.72, 28.57, 24.19, 22.88, 21.57 ppm;

**HRMS** (ESI): calculated for $[C_{32}H_{41}NO_5S+H]^+$ 552.2778, found 552.2780.

## Synthesis of compound S4

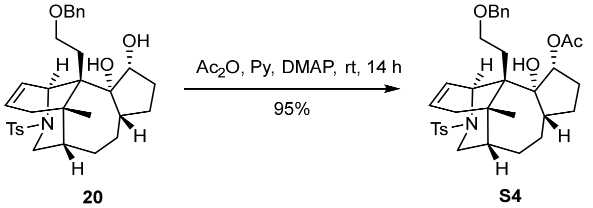


20 S4

To a solution of compound **20** (850 mg, 1.50 mmol, 1.0 equiv) and DMAP (25 mg, 0.15 mmol, 0.10 equiv) in pyridine (50 mL), was added acetic anhydride (1.40 mL, 15.0 mmol, 10 equiv) dropwise and

stirred for 14 h at room temperature. MeOH (20 mL) was added and stirred for another 0.5 h. Volatiles were removed under reduced pressure, and the saturated aqueous $NaHCO_3$ (100 mL) was added. The aqueous phase was extracted with DCM (3 × 50 mL). The combined organic phases were washed with brine, dried over anhydrous $Na_2SO_4$, filtered, and concentrated under reduced pressure. The residue was purified by flash chromatography (eluent: EtOAc/PE = 0/1 to 1/8) to give compound **S4** (846 mg, yield: 95%) as a yellow foam.

**Rf** = 0.60 (silica, EtOAc/PE = 1:4); **$[\alpha]_D^{20}$** = +87.3 (*c* 1.0, $CHCl_3$);

**$^1$H NMR** (400 MHz, $CDCl_3$) δ 7.63 (d, *J* = 8.3 Hz, 2H), 7.31 – 7.21 (m, 7H), 5.72 (dt, *J* = 9.7, 3.3 Hz, 1H), 5.04 (ddd, *J* = 9.0, 6.5, 2.1 Hz, 1H), 4.55 – 4.46 (m, 2H), 4.42 – 4.34 (m, 2H), 4.31 – 4.21 (m, 1H), 3.66 (d, *J* = 2.1 Hz, 1H), 3.60 (d, *J* = 11.8 Hz, 1H), 3.36 – 3.26 (m, 2H), 2.44 (s, 3H), 2.41 – 2.31 (m, 1H), 2.31 – 2.16 (m, 3H), 2.04 (s, 3H), 1.96 – 1.82 (m, 2H), 1.80 – 1.71 (m, 2H), 1.65 – 1.59 (m, 3H), 1.56 – 1.44 (m, 2H), 1.33 (dt, *J* = 13.1, 3.7 Hz, 1H), 1.12 (s, 3H) ppm;

**$^{13}$C NMR** (101 MHz, $CDCl_3$) δ 170.89, 143.86, 138.59, 135.66, 132.85, 129.83 (2C), 128.21 (2C), 127.30 (2C), 127.21, 127.03 (2C), 119.24, 86.39, 81.72, 72.55, 69.22, 52.58, 50.03, 48.68, 47.19, 45.48, 45.43, 40.44, 35.54, 32.69, 28.14, 28.09, 23.84, 22.84, 21.60, 21.48 ppm;

**HRMS** (ESI): calculated for $[C_{34}H_{43}NO_6S+Na]^+$ 616.2703, found 616.2702.

**Synthesis of compound 21**

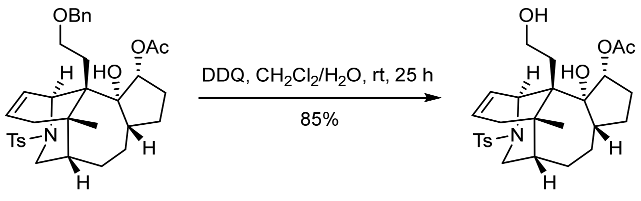


S4 21

DDQ (477 mg, 2.10 mmol, 5.0 equiv) was added to a solution of the substrate **S4** (250 mg, 0.420 mmol, 1.0 equiv) in DCM (50 mL) and $H_2O$ (2.5 mL) at room temperature and stirred for 25 h. The reaction mixture was diluted with DCM, quenched with a saturated aqueous $NaHCO_3$ solution, and extracted with DCM (3 × 30 mL). The combined organic phase was sequentially washed with a saturated aqueous $NaHCO_3$ solution and brine, dried over $Na_2SO_4$, concentrated and the residue was purified by column chromatography on silica gel (eluent: EtOAc/PE = 1/2 to 2/3) to give compound **21** (180 mg, yield: 85%) as a white foam.

**Rf** = 0.40 (silica, EtOAc/PE = 1:1);

$[\alpha]_D^{20}$ = +105.2 (*c* 1.0, $CHCl_3$);

**$^1$H NMR** (400 MHz, $CDCl_3$) δ 7.63 (d, *J* = 8.3 Hz, 2H), 7.29 (d, *J* = 8.1 Hz, 2H), 5.75 (dt, *J* = 9.4, 3.3 Hz, 1H), 5.04 (ddd, *J* = 9.0, 6.4, 2.1 Hz, 1H), 4.52 (d, *J* = 6.3 Hz, 1H), 4.49 – 4.43 (m, 1H), 4.32 – 4.22 (m, 1H), 3.66 (d, *J* = 2.0 Hz, 1H), 3.60 (d, *J* = 11.8 Hz, 1H), 3.51 (q, *J* = 8.9 Hz, 1H), 3.30 (ddd, *J* = 11.8, 5.1, 2.1 Hz, 1H), 2.43 (s, 3H), 2.39 – 2.29 (m, 1H), 2.29 – 2.17 (m, 3H), 2.15 (s, 3H), 1.94 – 1.82 (m, 2H), 1.81 – 1.72 (m, 2H), 1.60 (q, *J* = 4.2 Hz, 1H), 1.56 – 1.49 (m, 3H), 1.32 (dt, *J* = 13.0, 3.7 Hz, 1H), 1.16 (s, 1H), 1.11 (s, 3H) ppm;

**$^{13}$C NMR** (101 MHz, $CDCl_3$) δ 171.07, 143.95, 135.62, 133.14, 129.85 (2C), 127.31 (2C), 119.23, 86.43, 81.70, 61.14, 52.44, 50.11, 48.59, 47.26, 45.44, 45.34, 40.36, 35.65, 35.42, 28.20, 28.06, 23.80, 22.85, 21.76, 21.58 ppm;

**HRMS** (ESI): calculated for $[C_{27}H_{37}NO_6S+H]^+$ 504.2412, found 504.2412.

**Synthesis of compound 22**

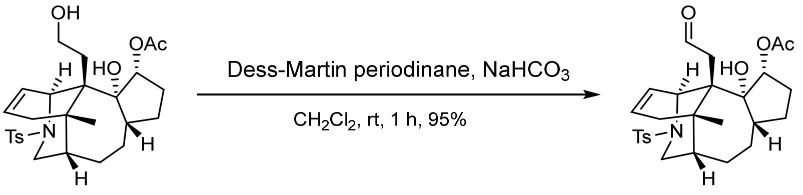


**21** **22**

To a suspension of compound **21** (250 mg, 0.490 mmol, 1.0 equiv) and $NaHCO_3$ (212 mg, 2.00 mmol, 4.0 equiv) in DCM (80 mL) was added Dess-Martin periodinane (3.39 g, 8.00 mmol, 2.0 equiv) in one portion. After being stirred for 1 h, the mixture was cooled to 0 °C and quenched with saturated aqueous $NaHCO_3$ (40 mL) and saturated aqueous $Na_2S_2O_3$ (40 mL). The aqueous phase was extracted with DCM (3 × 50 mL). The combined organic phases were washed with brine, dried over anhydrous $Na_2SO_4$, filtered, and concentrated under reduced pressure. The residue was purified by flash chromatography (eluent: EtOAc/PE = 1/8 to 1/4 to 1/2) to give compound **22** (236 mg, yield: 95%) as a yellow foam.

**Rf** = 0.55 (silica, EtOAc/PE = 1:2);

$[\alpha]_D^{20}$ = +95.0 (*c* 1.0, $CHCl_3$);

**mp**: 228–230 °C;

**$^1$H NMR** (400 MHz, $CDCl_3$) δ 10.05 (s, 1H), 7.65 (d, *J* = 8.3 Hz, 2H), 7.30 (d, *J* = 8.2 Hz, 2H), 5.81 (dt, *J* = 9.7, 3.3 Hz, 1H), 5.25 (d, *J* = 6.4 Hz, 1H), 4.90 (ddd, *J* = 9.1, 6.6, 2.1 Hz, 1H), 4.43 (ddt, *J* = 8.7, 6.5, 2.1 Hz, 1H), 3.91 (d, *J* = 2.1 Hz, 1H), 3.67 (d, *J* = 12.0 Hz, 1H), 3.30 (ddd, *J* = 12.0, 5.0, 2.1

Hz, 1H), 2.44 – 2.39 (m, 4H), 2.37 – 2.31 (m, 1H), 2.28 – 2.20 (m, 2H), 2.15 – 2.07 (m, 1H), 2.06 – 2.00 (m, 1H), 1.96 (s, 3H), 1.93 – 1.90 (m, 1H), 1.90 – 1.84 (m, 1H), 1.84 – 1.76 (m, 1H), 1.67 – 1.60 (m, 1H), 1.54 (dddd, *J* = 13.7, 11.4, 6.6, 2.7 Hz, 2H), 1.40 (dt, *J* = 12.9, 3.8 Hz, 1H), 1.11 (s, 3H) ppm;

**$^{13}$C NMR** (101 MHz, $CDCl_3$) δ 202.46, 170.43, 144.19, 135.39, 135.07, 129.94 (2C), 127.34 (2C), 118.25, 85.41, 81.86, 52.37, 48.87, 47.90, 46.60, 45.32, 44.87, 44.77, 39.89, 35.02, 28.11, 27.75, 23.74, 22.91, 21.60, 21.43 ppm;

**HRMS** (ESI): calculated for $[C_{27}H_{35}NO_6S+H]^+$ 502.2258, found 502.2257.

**Synthesis of compound 24**

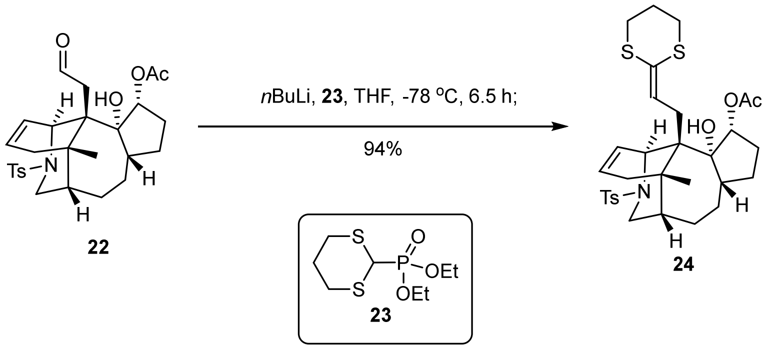


To a cooled (–78 °C) solution of diethyl (1,3-dithian-2-yl) phosphonate**(23)** (512 mg, 4.00 mmol, 4.0 equiv) in THF (15 mL), was added *n*-BuLi (0.83 mL, 2.4 M in hexane, 4.0 equiv) dropwise and stirred for 1 h at that temperature. A solution of compound **22** (250 mg, 0.498 mmol, 1.0 equiv) in THF (10 mL) was added dropwise over 1 h. After being stirred for 3.5 h at –78 °C, the mixture was warmed to 0 °C and stirred for 2.5 h. The reaction was quenched with saturated aqueous $NH_4Cl$ (20 mL). Volatiles were removed under reduced pressure, and the aqueous phase was extracted with DCM (3 × 40 mL). The combined organic phases were washed with brine, dried over anhydrous $Na_2SO_4$, filtered, and concentrated under reduced pressure. The residue was purified by flash chromatography (eluent: EtOAc/PE/DCM = 1/8/1 to 1/3/1) to give compound **24** (285 mg, yield: 94%) as a white solid.

**Rf** = 0.45 (silica, EtOAc/PE = 1:4);

**$[\alpha]_D^{20}$** = +137.1 (*c* 0.5, $CHCl_3$);

**mp**: 218–220 °C;

**$^1$H NMR** (400 MHz, $CDCl_3$) δ 7.64 (d, *J* = 8.3 Hz, 2H), 7.28 (d, *J* = 8.1 Hz, 2H), 6.64 (dd, *J* = 10.1, 2.5 Hz, 1H), 5.73 (dt, *J* = 9.7, 3.3 Hz, 1H), 5.00 (ddd, *J* = 9.1, 6.5, 2.1 Hz, 1H), 4.72 (d, *J* = 6.4 Hz, 1H), 4.41 – 4.30 (m, 1H), 3.75 (d, *J* = 2.1 Hz, 1H), 3.59 (d, *J* = 11.8 Hz, 1H), 3.26 (ddd, *J* = 11.9, 5.1, 2.0

Hz, 1H), 2.76 (ddd, $J$ = 6.8, 4.9, 3.5 Hz, 2H), 2.67 (t, $J$ = 5.9 Hz, 2H), 2.50 – 2.41 (m, 4H), 2.32 – 2.18 (m, 4H), 2.05 (s, 5H), 1.96 (dd, $J$ = 16.9, 2.6 Hz, 1H), 1.91 – 1.82 (m, 2H), 1.82 – 1.71 (m, 2H), 1.63 – 1.57 (m, 1H), 1.57 – 1.43 (m, 2H), 1.38 – 1.30 (m, 1H), 1.13 (s, 3H) ppm;

**$^{13}$C NMR** (101 MHz, $CDCl_3$) δ 170.71, 143.80, 135.61, 134.93, 132.85, 129.76 (2C), 127.43 (2C), 125.07, 118.87, 86.15, 81.55, 52.92, 51.38, 48.16, 47.44, 45.55, 45.19, 40.74, 35.07, 33.27, 30.68, 29.91, 28.26, 27.77, 25.44, 23.81, 22.99, 21.61, 21.34 ppm;

**HRMS** (ESI): calculated for $[C_{31}H_{41}NO_5S_3+H]^+$ 604.2220, found 604.2219.

### Synthesis of compound 25

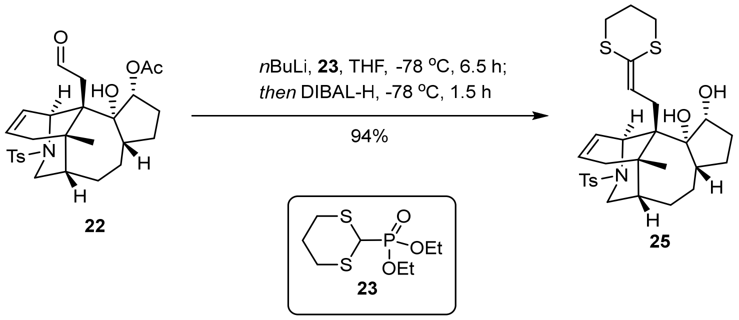


To a cooled (–78 °C) solution of diethyl(1,3-dithian-2-yl)phosphonate **(23)** (513 mg, 2.00 mmol, 4.0 equiv) in THF (20 mL), was added *n*-BuLi (0.83 mL, 2.4 M in hexane, 4.0 equiv) dropwise and stirred for 1 h at that temperature. A solution of compound **22** (251 mg, 0.500 mmol, 1.0 equiv) in THF (10 mL) was added dropwise over 1 h. After being stirred for 3.5 h at –78 °C, the mixture was warmed to 0 °C and stirred for 1 h. The reaction was cooled to –78 °C and DIBAL-H (2.5 mL, 1M in hexane, 5.0 equiv) was added. After being stirred for 1.5 h, the reaction was quenched with saturated aqueous potassium sodium tartrate (50 mL) and stirred for 1 h at room temperature. The aqueous phase was extracted with DCM (3 × 30 mL). The combined organic phases were washed with brine, dried over anhydrous $Na_2SO_4$, filtered, and concentrated under reduced pressure. The residue was purified by flash chromatography (eluent: EtOAc/PE = 1/3) to give compound **25** (264 mg, yield: 94 %) as a white foam.

**Rf** = 0.25 (silica, EtOAc/PE = 1:4);

**$[\alpha]_D^{20}$** = +65.0 ($c$ 1.0, $CHCl_3$);

**$^1$H NMR** (400 MHz, $CDCl_3$) δ 7.64 (d, $J$ = 8.3 Hz, 2H), 7.28 (d, $J$ = 8.1 Hz, 2H), 6.54 (dd, $J$ = 9.7, 3.4 Hz, 1H), 5.74 (dt, $J$ = 9.7, 3.3 Hz, 1H), 4.66 (d, $J$ = 6.4 Hz, 1H), 4.35 (ddt, $J$ = 8.8, 6.4, 2.1 Hz, 1H), 4.10 – 4.00 (m, 1H), 3.79 (d, $J$ = 1.8 Hz, 1H), 3.58 (d, $J$ = 11.8 Hz, 1H), 3.27 (ddd, $J$ = 11.8, 5.2, 2.0

Hz, 1H), 2.86 (ddd, $J$ = 12.9, 8.7, 3.9 Hz, 1H), 2.78 – 2.56 (m, 4H), 2.46 – 2.34 (m, 5H), 2.28 (dt, $J$ = 19.9, 2.9 Hz, 1H), 2.25 – 2.02 (m, 5H), 1.93 – 1.69 (m, 4H), 1.60 (q, $J$ = 4.1 Hz, 1H), 1.48 – 1.36 (m, 2H), 1.31 (dt, $J$ = 13.0, 3.6 Hz, 1H), 1.13 (s, 3H) ppm;

**$^{13}$C NMR** (101 MHz, $CDCl_3$) δ 143.80, 137.17, 135.61, 132.82, 129.78 (2C), 127.34 (2C), 124.04, 119.06, 85.52, 80.81, 53.18, 51.64, 48.42, 47.42, 45.59, 45.28, 40.84, 35.22, 33.41, 32.03, 31.01, 30.12, 28.71, 25.71, 24.29, 23.02, 21.59 ppm;

**HRMS** (ESI): calculated for $[C_{29}H_{39}NO_4S_3+H]^+$ 562.2114, found 562.2114.

**Synthesis of compound 26**

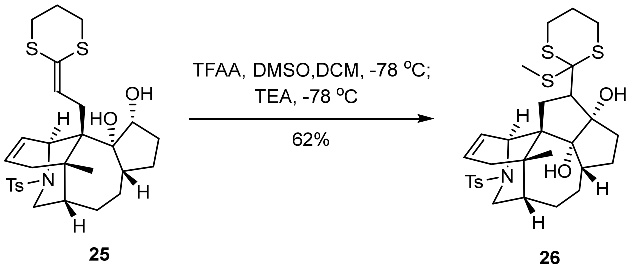


To a solution of DMSO (2.13 mL, 30.0 mmol, 60 equiv) in DCM (25 mL), was added TFAA (2.12 mL, 15.0 mmol, 30 equiv) dropwise at –78 °C and stirred for 40 min. A solution of compound **25** (280 mg, 0.500 mmol, 1.0 equiv) in DCM (30 mL) was added dropwise over 15 min at that temperature. After being stirred for another 3 h at –78 °C, TEA (6.25 mL, 45 mmol, 90 equiv) was added dropwise over 30 min and stirred for another 2 h at that temperature. The reaction was quenched with water (30 mL) and extracted with DCM (4 × 20 mL). The combined organic phases were washed with brine, dried over anhydrous $Na_2SO_4$, filtered, and concentrated under reduced pressure. The residue was purified by flash chromatography on silica gel (eluent: EtOAc/PE = 1/20 to 1/10 to EtOAc/PE/DCM = 1/6/1) to give in order of elution, compound **26** (189 mg, yield: 62 %) as a yellow foam.

**Rf** = 0.38 (silica, EtOAc/PE = 1:4);

**$[\alpha]_D^{20}$** = +68.5 ($c$ 1.0, $CHCl_3$);

**$^1$H NMR** (400 MHz, $CDCl_3$) δ 7.71 (d, $J$ = 8.1 Hz, 2H), 7.27 (d, $J$ = 8.2 Hz, 2H), 5.78 (dt, $J$ = 9.6, 3.4 Hz, 1H), 5.08 (dd, $J$ = 9.5, 6.7 Hz, 1H), 4.54 (d, $J$ = 7.8 Hz, 2H), 4.17 (s, 1H), 3.45 – 3.20 (m, 3H), 3.10 (dd, $J$ = 12.1, 5.4 Hz, 1H), 2.66 – 2.49 (m, 2H), 2.42 (s, 3H), 2.36 (dd, $J$ = 13.7, 4.6 Hz, 1H), 2.32 – 2.16 (m, 3H), 2.16 – 2.08 (m, 4H), 2.08 – 1.77 (m, 8H), 1.66 – 1.54 (m, 2H), 1.52 – 1.40 (m, 2H), 1.34 (s, 3H) ppm;

**$^{13}$C NMR** (101 MHz, $CDCl_3$) δ 143.65, 135.49, 133.60, 129.46 (2C), 128.16 (2C), 119.40, 91.80, 90.11, 64.05, 56.38, 56.08, 47.80, 46.98, 46.42, 46.37, 44.47, 40.61, 39.58, 38.74, 32.82, 32.71, 27.72, 27.20, 27.00, 24.67, 23.78, 21.58, 17.50 ppm;

**HRMS** (ESI): calculated for $[C_{30}H_{41}NO_4S_4+H]^+$ 608.1991, found 608.1988.

**Synthesis of compound 27**

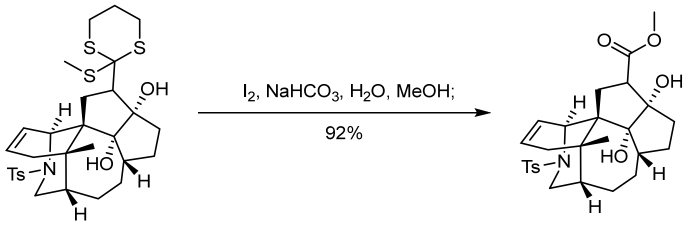


**26** **27**

To a solution of compound **26** (152 mg, 0.250 mmol, 1.00 equiv) in MeOH (20 mL), DCM (10 mL) and water (0.5 mL) were sequentially added $NaHCO_3$ (286 mg, 3.40 mmol, 13.6 equiv) and $I_2$ (431 mg, 1.70 mmol, 6.80 equiv). The mixture was stirred for 10 min and cooled to 0 °C. After being quenched with saturated aqueous $NaHCO_3$ (20 mL) and saturated aqueous $Na_2S_2O_3$ (20 mL), the resulting mixture was filtered and concentrated under reduced pressure. The residue was extracted with DCM (3 × 20 mL) and the combined organic phase was sequentially washed with a saturated aqueous brine, dried over $Na_2SO_4$, concentrated. The residue was purified by column chromatography on silica gel (eluent: EtOAc/PE = 1/8 to 1/4) to give compound **27** (116 mg, yield: 92 %) as a white foam.

**Rf** = 0.25 (silica, EtOAc/PE = 1:4);

**$[\alpha]_D^{20}$** = +93.0 (*c* 1.0, $CHCl_3$);

**$^{1}$H NMR** (400 MHz, $CDCl_3$) δ 7.66 (d, *J* = 8.3 Hz, 2H), 7.30 (d, *J* = 8.1 Hz, 2H), 5.74 (dt, *J* = 9.7, 3.4 Hz, 1H), 5.00 – 4.93 (m, 1H), 4.69 (s, 1H), 4.50 (d, *J* = 6.4 Hz, 1H), 4.22 (s, 1H), 3.70 (s, 3H), 3.44 – 3.27 (m, 2H), 3.10 (dd, *J* = 11.9, 5.0 Hz, 1H), 2.69 (dd, *J* = 13.5, 5.6 Hz, 1H), 2.47 – 2.37 (m, 4H), 2.21 – 1.92 (m, 5H), 1.84 – 1.77 (m, 2H), 1.69 – 1.49 (m, 4H), 1.31 – 1.26 (m, 4H) ppm;

**$^{13}$C NMR** (101 MHz, $CDCl_3$) δ 172.38, 144.15, 134.83, 133.94, 129.68 (2C), 128.08 (2C), 118.80, 91.16, 90.01, 56.32, 52.10, 51.63, 48.77, 46.49, 46.24, 46.18, 44.56, 39.34, 38.36, 38.12, 32.45, 32.23, 26.83, 23.92, 21.58 ppm;

**HRMS** (ESI): calculated for $[C_{27}H_{35}NO_6S+H]^+$ 502.2258, found 502.2255.

**Synthesis of compound 29**

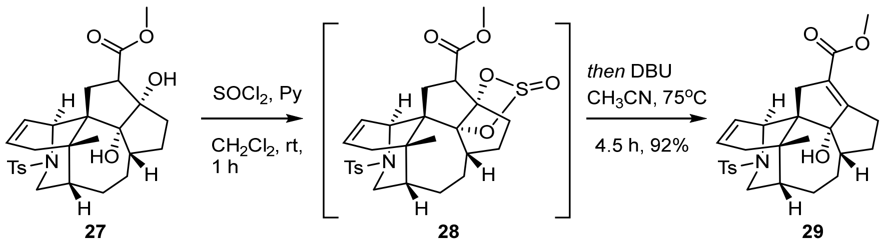


To a solution of compound **27** (251 mg, 0.50 mmol, 1.0 equiv) in pyridine (25 mL) was added $SOCl_2$ (0.60 mL, 0.60 mmol, 1.0 M in DCM, 1.2 equiv) dropwise and stirred for 1 h at room temperature. The mixture was cooled to 0 °C and quenched with saturated aqueous MeOH (5 mL). The mixture was concentrated under reduced pressure and the residue was dissolved in MeCN (50 mL). DBU (1.49 mL, 10.0 mmol, 20 equiv) was added to the resulting solution at room temperature. The mixture was stirred for 4.5 h at 75 °C. Volatiles were removed under reduced pressure after the reaction was cooled to room temperature. The residue was purified by flash chromatography (eluent: EtOAc/PE = 0/1 to 1/20 to EtOAc/PE/DCM = 1/6/1) to give compound **29** (222 mg, yield: 92%) as a white foam.

**Rf** = 0.40 (silica, EtOAc/PE = 1:4);

**$[\alpha]_D^{20}$** = +114.9 (*c* 0.5, $CHCl_3$);

**$^1$H NMR** (400 MHz, $CDCl_3$) δ 7.65 (d, *J* = 8.3 Hz, 2H), 7.29 (d, *J* = 8.0 Hz, 2H), 5.77 (dt, *J* = 9.4, 3.4 Hz, 1H), 4.71 (d, *J* = 6.3 Hz, 1H), 4.66 (ddd, *J* = 9.5, 5.3, 3.1 Hz, 1H), 3.70 (s, 3H), 3.67 (s, 1H), 3.63 (d, *J* = 11.9 Hz, 1H), 3.20 (ddd, *J* = 12.0, 5.0, 1.5 Hz, 1H), 2.71 – 2.61 (m, 2H), 2.61 – 2.51 (m, 2H), 2.43 (s, 3H), 2.40 – 2.29 (m, 1H), 2.28 – 2.11 (m, 2H), 2.06 – 1.90 (m, 2H), 1.85 – 1.69 (m, 3H), 1.71 – 1.65 (m, 1H), 1.62 – 1.54 (m, 1H), 1.08 (s, 3H) ppm;

**$^{13}$C NMR** (101 MHz, $CDCl_3$) δ 165.95, 165.50, 143.83, 135.63, 134.85, 129.75 (2C), 127.56 (2C), 121.63, 118.84, 94.43, 54.93, 51.24, 49.82, 47.39, 45.96, 45.31, 44.70, 44.03, 38.40, 35.00, 31.30, 25.01, 23.87, 23.16, 21.59 ppm;

**HRMS** (ESI): calculated for $[C_{27}H_{33}NO_5S+Na]^+$ 506.1972, found 506.1969.

**Synthesis of compound 30**

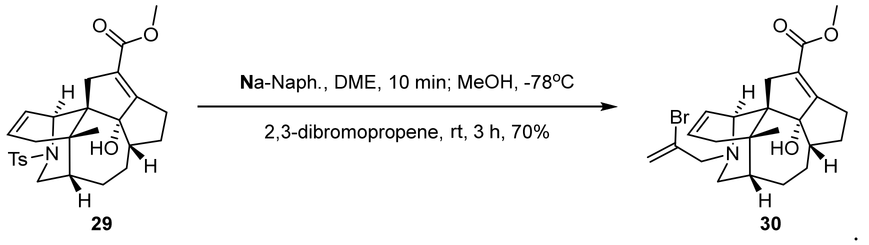

To a solution of naphthalene (1.28 g, 10.0 mmol) in DME (20 mL) was added metal sodium (230 mg, 10.0 mmol) at room temperature. The mixture was stirred at room temperature for 1 h to yield a deep-blue sodium-naphthalenide solution (0.5 M). This fresh-prepared solution was added slowly to a solution of compound **29** (121 mg, 0.25 mmol, 1.0 equiv) in THF (10 ml) at −78 °C (about 2.0 mL, 4.0 equiv was used). After being stirred at −78 °C for 10 min, MeOH (10 mL) was added. The resulting solution was warmed up to room temperature, then 2,3-dibromopropene (0.470 mL, 5.00 mmol, 20 equiv) was added. After being stirred at room temperature for 3 h, the reaction mixture was concentrated under reduced pressure. The residue was purified by flash chromatography on silica gel (eluent: EtOAc/PE = 0/1 to EtOAc/PE = 1/10) to give compound **30** (78 mg, yield: 70%) as a pale-yellow oil.

**Rf** = 0.65 (silica, EtOAc/PE = 1:4);

$[\alpha]_D^{20}$ = +3.7 (*c* 1.0, $CHCl_3$);

$^1$**H NMR** (400 MHz, $CDCl_3$) δ 6.13 (dt, *J* = 9.8, 3.5 Hz, 1H), 6.05 (s, 1H), 5.73 (s, 1H), 5.57 (s, 1H), 5.49 – 5.41 (m, 1H), 3.70 (s, 3H), 3.60 (d, *J* = 5.8 Hz, 1H), 3.36 (d, *J* = 13.1 Hz, 1H), 2.89 (d, *J* = 13.2 Hz, 1H), 2.78 (d, *J* = 17.1 Hz, 1H), 2.65 – 2.48 (m, 5H), 2.40 – 2.28 (m, 1H), 2.24 – 1.91 (m, 4H), 1.88 – 1.72 (m, 2H), 1.71 – 1.67 (m, 1H), 1.62 – 1.54 (m, 1H), 1.54 – 1.45 (m, 1H), 1.10 (s, 3H) ppm;

$^{13}$**C NMR** (101 MHz, $CDCl_3$) δ 166.25, 166.01, 135.03, 131.05, 121.67, 120.20, 118.44, 94.87, 64.77, 61.27, 51.15, 49.63, 48.50, 46.99, 46.22, 45.40, 44.15, 38.20, 34.32, 31.47, 24.67, 24.05, 23.73 ppm;

**HRMS** (ESI): calculated for $[C_{23}H_{30}BrNO_3+H]^+$ 448.1482, found 448.1484

**Synthesis of compound 31**

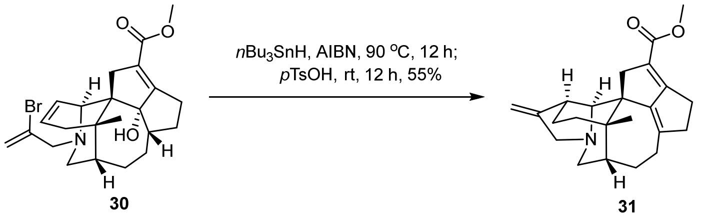

A solution of $^{n}Bu_3SnH$ (0.055 mL, 0.20 mmol, 2.0 equiv) and AIBN (6.6 mg, 0.040 mmol, 0.40 equiv) in PhMe (2 mL) was added dropwise over 0.5 h to a solution of compound **30** (44.8 mg, 0.10 mmol, 1.0 equiv) in PhMe (5 mL) at 90 °C. After being stirred for another 12 h at 90 ºC, the solution was cooled to room temperature. PhMe was removed under reduced pressure and the residue was dissolved in DCM (5 mL), *p*-toluenesulfonic acid monohydrate (190 mg, 1.00 mmol, 10 equiv) was to the resulting solution at room temperature. After being stirred for 12 h, the reaction mixture was quenched with saturated aqueous $NaHCO_3$ (15 mL) and 15% aqueous NaOH (1.5 mL). After being stirred for 30 min, the organic phase was separated and the aqueous phase was extracted with DCM (4 × 8 mL). The combined organic phases were washed with brine, dried over anhydrous $Na_2SO_4$, filtered, and concentrated under reduced pressure. The residue was purified by flash chromatography on silica gel (eluent: EtOAc/PE, 1/10, then TEA/MeOH/DCM, 1/10/100) to give compound **31** (19.3 mg, yield: 55%) as a colorless oil.

**Rf** = 0.35 (silica, MeOH/DCM = 1:20);

**$[\alpha]_D^{20}$** = +57.4 (*c* 1.0, $CHCl_3$);

**$^1$H NMR** (400 MHz, $CD_3OD$) δ 5.08 (s, 1H), 4.98 (d, J = 1.7 Hz, 1H), 3.70 (s, 3H), 3.50 – 3.41 (m, 1H), 3.39 – 3.32 (m, 1H), 3.14 (dd, J = 12.6, 7.1 Hz, 1H), 3.07 (d, J = 15.4 Hz, 1H), 2.99 (d, J = 12.7 Hz, 1H), 2.93 – 2.85 (m, 3H), 2.85 – 2.72 (m, 2H), 2.72 – 2.51 (m, 3H), 2.21 – 2.03 (m, 3H), 1.93 (t, J = 6.7 Hz, 1H), 1.75 – 1.46 (m, 4H), 0.91 (s, 3H) ppm;

**$^{13}$C NMR** (101 MHz, $CD_3OD$) δ 171.62, 168.76, 158.01, 154.39, 151.64, 117.14, 106.87, 68.85, 63.35, 55.55, 51.51, 47.33, 44.84, 44.17, 43.07, 39.68, 37.83, 34.42, 31.27, 30.68, 27.29, 26.48, 24.87 ppm;

**HRMS** (ESI): calculated for $[C_{23}H_{29}NO_2+H]^+$ 352.2271, found 352.2271.

**Synthesis of (+)-Caldaphnidine J**

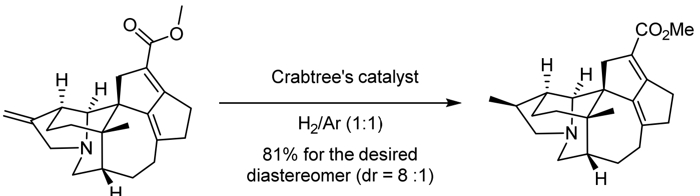


Under Ar/$H_2$ (1/1) atmosphere, a solution of compound **31** (8.9 mg, 0.025 mmol, 1.0 equiv) and Crabtree's catalyst (24.2 mg, 0.030 mmol, 1.2 equiv) in DCM (5 mL) was stirred for 17 h at room temperature. The mixture was concentrated under reduced pressure and subjected to HPLC (Gilson GX-281, S ACE 5 super-C18 column,150*10 mm, 296 nm) for purification using B/A = 72/28 (7 mL/min, B: MeCN/HCOOH = 100/1, A: water/ammonium hydroxide/HCOOH = 100/2/1) as eluent to give **(+)-Caldaphnidine J** (7.2 mg, yield for desired diastereomer: 81%, $t_R$ = 20.8 min) as a colorless oil.

**Rf** = 0.55 (silica, MeOH/DCM = 1:10);

**$[\alpha]_D^{20}$** = +60.0 (*c* 0.1, MeOH);

**$^1$H NMR** (500 MHz, $CD_3OD$) δ 3.70 (s, 3H), 3.47 – 3.40 (m, 1H), 3.40 – 3.35 (m, 1H), 3.10 (brd, J = 16.4 Hz, 1H), 2.96 – 2.91 (m, 2H), 2.91 – 2.87 (m, 2H), 2.87 – 2.79 (m, 2H), 2.77 – 2.74 (m, 1H), 2.74 – 2.68 (m, 1H), 2.68 – 2.61 (m, 1H), 2.60 – 2.55 (m, 1H), 2.52 – 2.45 (m, 1H), 2.45 – 2.42 (m, 1H), 2.44 – 2.36 (m, 2H), 2.21 – 2.12 (m, 1H), 2.10 – 2.05 (m, 2H), 2.05 – 2.00 (m, 2H), 1.84 – 1.74 (m, 1H), 1.74 – 1.67 (m, 1H), 1.66 – 1.63 (m, 2H), 1.62 – 1.59 (m, 1H), 1.57 – 1.51 (m, 2H), 1.12 (d, J = 6.3 Hz, 3H), 0.93 (s, 3H) ppm;

**$^{13}$C NMR** (126 MHz, $CD_3OD$) δ171.69, 169.06, 153.76, 151.89, 117.77, 69.56, 66.00, 59.56, 51.95, 47.93, 44.94, 44.76, 43.52, 39.67, 38.70, 38.52, 35.63, 30.53, 26.93, 26.73, 25.07, 24.05, 15.62 ppm;

**HRMS** (ESI): calculated for $[C_{23}H_{31}NO_2+H]^+$ 354.2428, found 354.2425.

**Supplementary Table 1.** **Comparison of Caldaphnidine J $^{1}$H NMR Spectral Data with reported values**

| Yue's data[3] (400 MHz, $CD_3OD$) | Our synthetic data (500 MHz, $CD_3OD$) | Δ/ppm |
|---|---|---|
| 3.68 (s, 3H) | 3.70 (s, 3H) | 0.02 |
| 3.49 (m, 1H) | 3.44 (m, 1H) | 0.05 |
| 3.41(m, 1H) | 3.38 (m, 1H) | 0.03 |
| 3.34 (m, 1H) | 3.33 (m, 1H) | 0.01 |
| 3.09 (brd, 15.6, 1H) | 3.10 (brd, 16.4, 1H) | 0.01 |
| 2.96 (m, 1H) | 2.94 (m, 1H) | 0.02 |
| 2.89 (m, 1H) | 2.89 (m, 1H) | 0.00 |
| 2.83 (m, 1H) | 2.83 (m, 1H) | 0.00 |
| 2.72 (m, 1H) | 2.75 (m, 1H) | 0.03 |
| 2.69 (m, 1H) | 2.71 (m, 1H) | 0.02 |
| 2.69 (m, 1H) | 2.65 (m, 1H) | 0.04 |
| 2.60 (m, 1H) | 2.57 (m, 1H) | 0.03 |
| 2.52 (m, 1H) | 2.49 (m, 1H) | 0.03 |
| 2.44 (m, 1H) | 2.44 (m, 1H) | 0.00 |
| 2.44 (m, 1H) | 2.40 (m, 1H) | 0.04 |
| 2.16 (m, 1H) | 2.16 (m, 1H) | 0.00 |
| 2.07 (m, 1H) | 2.07 (m, 1H) | 0.00 |
| 2.05 (m, 1H) | 2.03 (m, 1H) | 0.02 |
| 1.79 (m, 1H) | 1.79 (m, 1H) | 0.00 |
| 1.69 (m, 1H) | 1.71 (m, 1H) | 0.02 |
| 1.65 (m, 1H) | 1.65 (m, 1H) | 0.00 |
| 1.63 (m, 1H) | 1.61 (m, 1H) | 0.02 |
| 1.54 (m, 1H) | 1.54 (m, 1H) | 0.00 |
| 1.11 (d, 6.1, 3H) | 1.12 (d, 6.3, 3H) | 0.01 |
| 0.92 (s, 3H) | 0.93 (s, 3H) | 0.01 |

**Supplementary Table 2.** **Comparison of Caldaphnidine J $^{13}C$ NMR Spectral Data with reported values**

| Yue's data[3] (101 MHz, $CD_3OD$, ppm) | Our synthetic data (126 MHz, $CD_3OD$, ppm) | Δ/ppm |
|---|---|---|
| 171.2 | 171.7 | 0.5 |
| 169.2 | 169.1 | 0.1 |
| 153.8 | 153.8 | 0.0 |
| 152.1 | 151.9 | 0.2 |
| 117.8 | 117.8 | 0.0 |
| 69.6 | 69.6 | 0.0 |
| 66.1 | 66.0 | 0.1 |
| 59.6 | 59.6 | 0.0 |
| 52.1 | 52.0 | 0.1 |
| 48.0 | 47.9 | 0.1 |
| 45.0 | 44.9 | 0.1 |
| 44.8 | 44.8 | 0.0 |
| 43.6 | 43.5 | 0.1 |
| 39.7 | 39.7 | 0.0 |
| 38.8 | 38.7 | 0.1 |
| 38.5 | 38.5 | 0.0 |
| 35.7 | 35.6 | 0.1 |
| 30.6 | 30.5 | 0.1 |
| 27.0 | 26.9 | 0.1 |
| 26.8 | 26.7 | 0.1 |
| 25.2 | 25.1 | 0.1 |
| 24.1 | 24.1 | 0.0 |
| 15.7 | 15.6 | 0.1 |

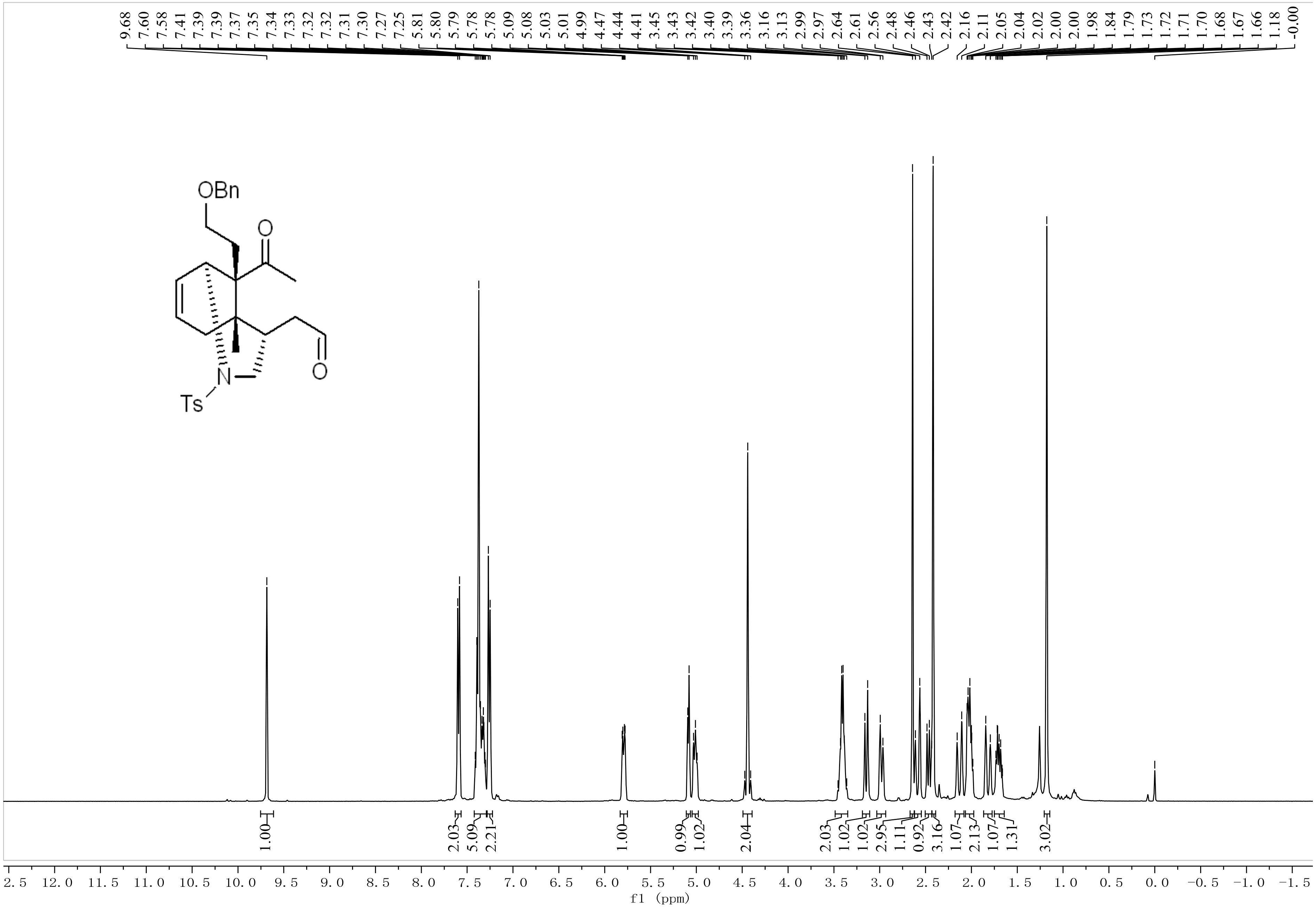

Supplementary Figure 3. $^{1}$H-NMR of compound 5

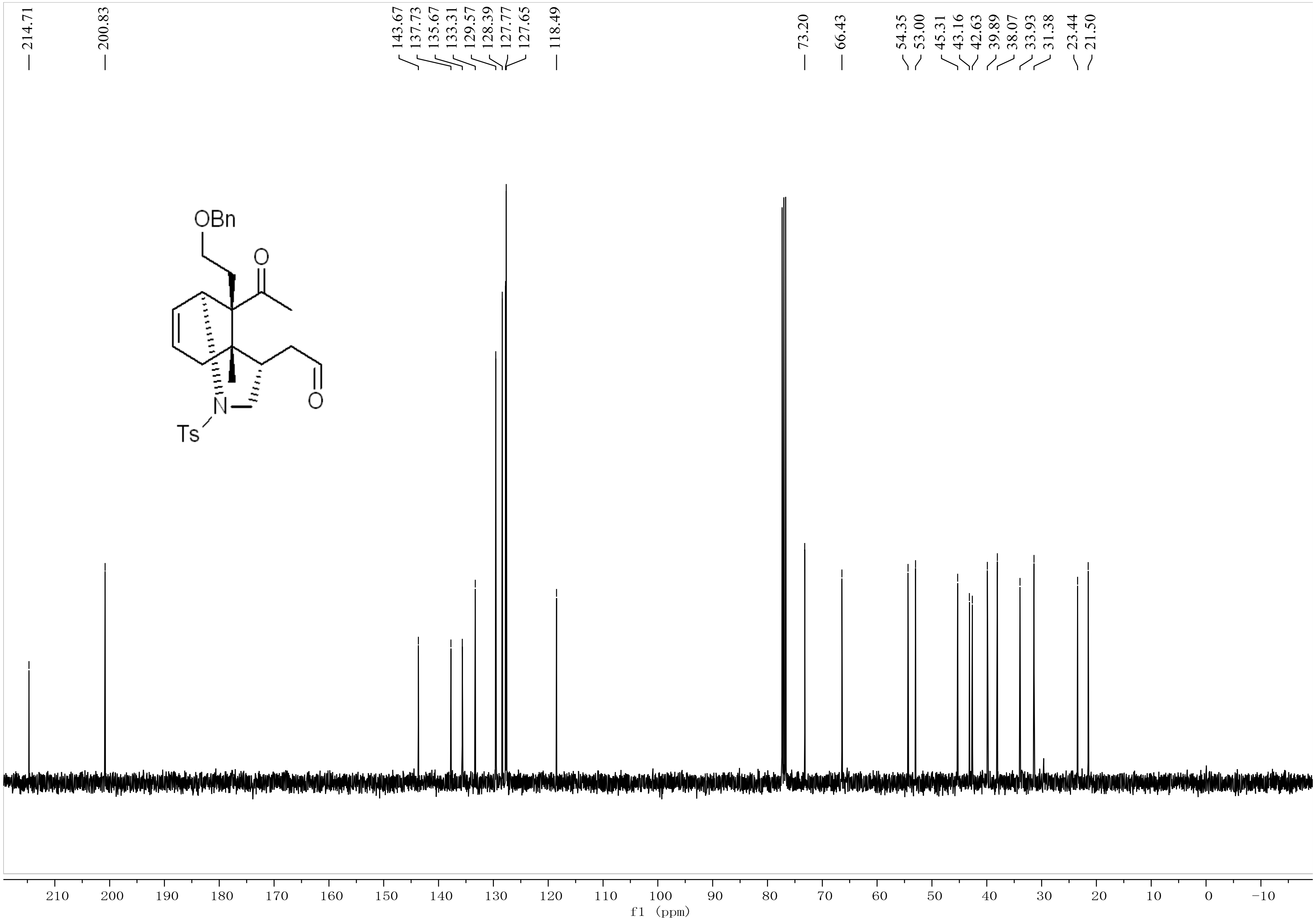


**Supplementary Figure 4.** **$^{13}$C-NMR of compound 5**

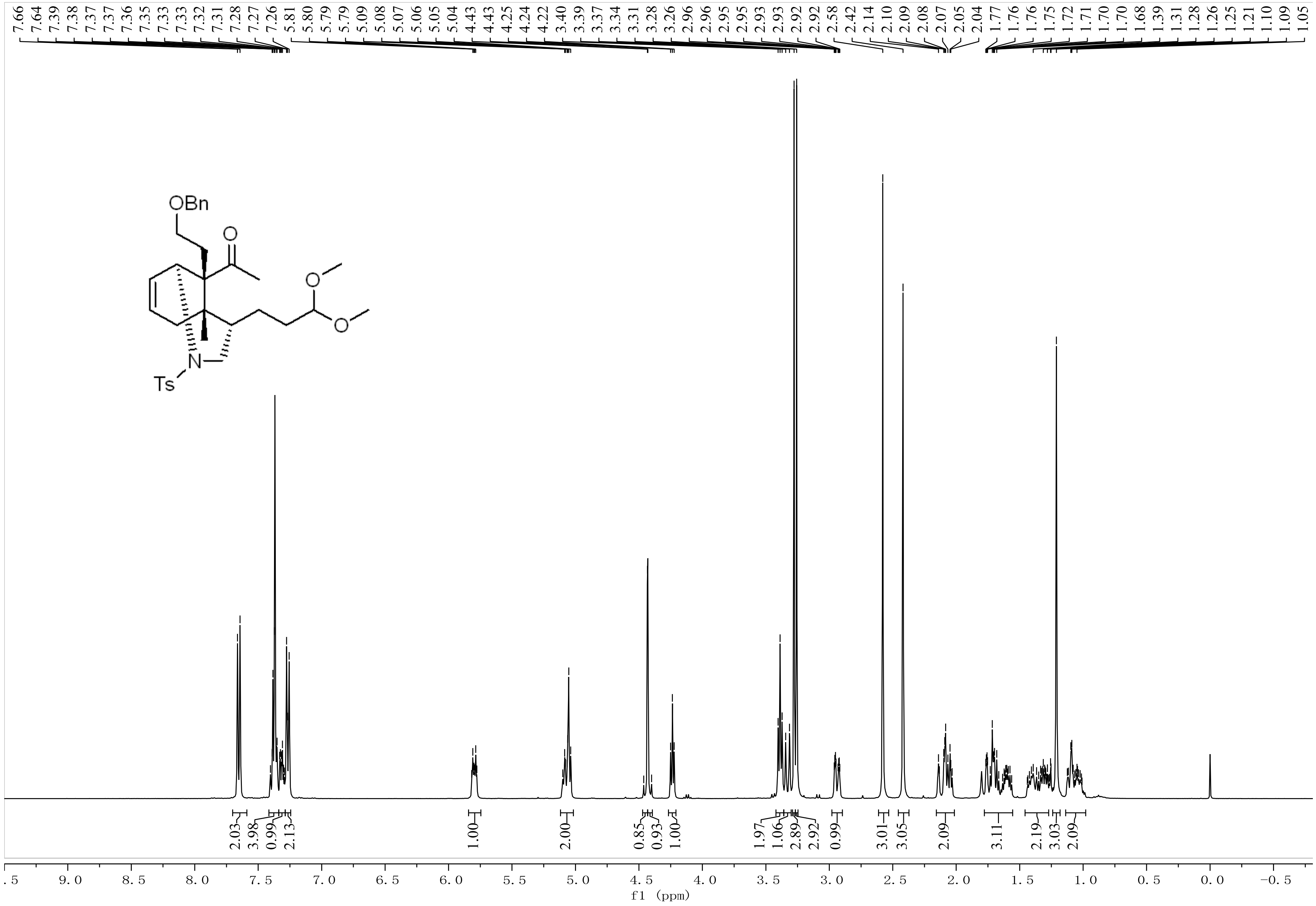

**Supplementary Figure 5.** $^{1}$H-NMR of compound 6

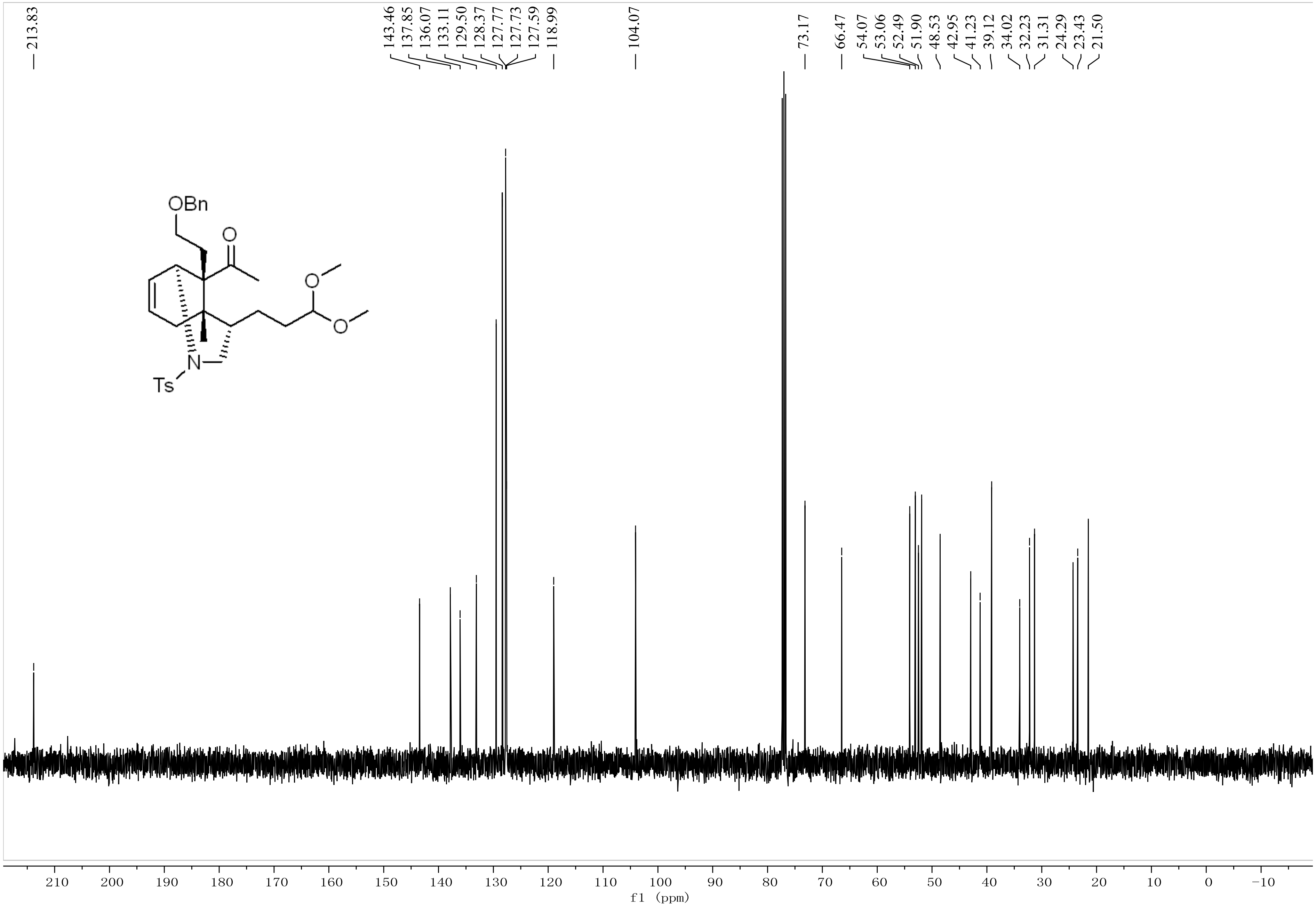


**Supplementary Figure 6.** $^{13}$C-NMR of compound 6

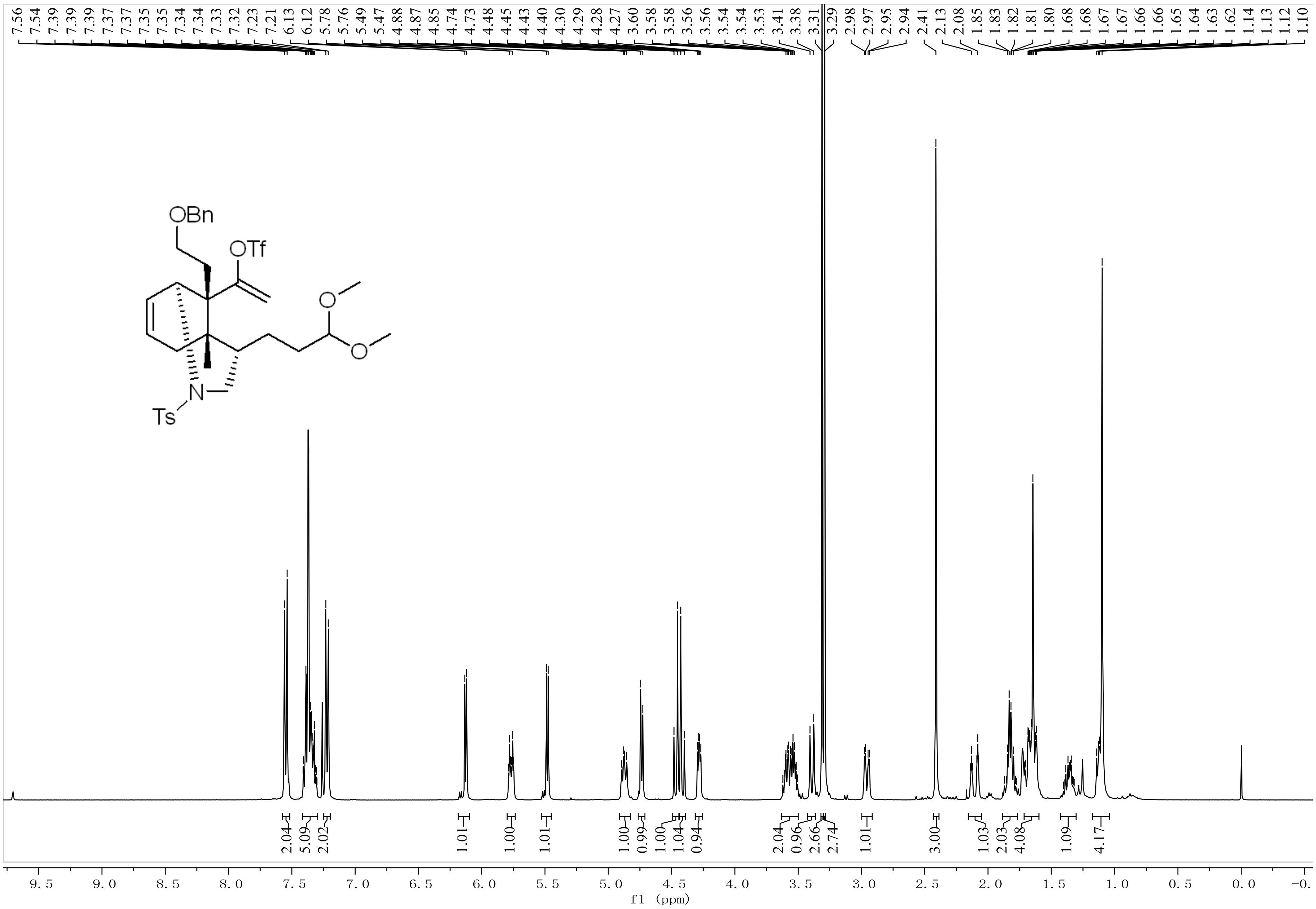

**Supplementary Figure 7.** $^{1}$H-NMR of compound S1

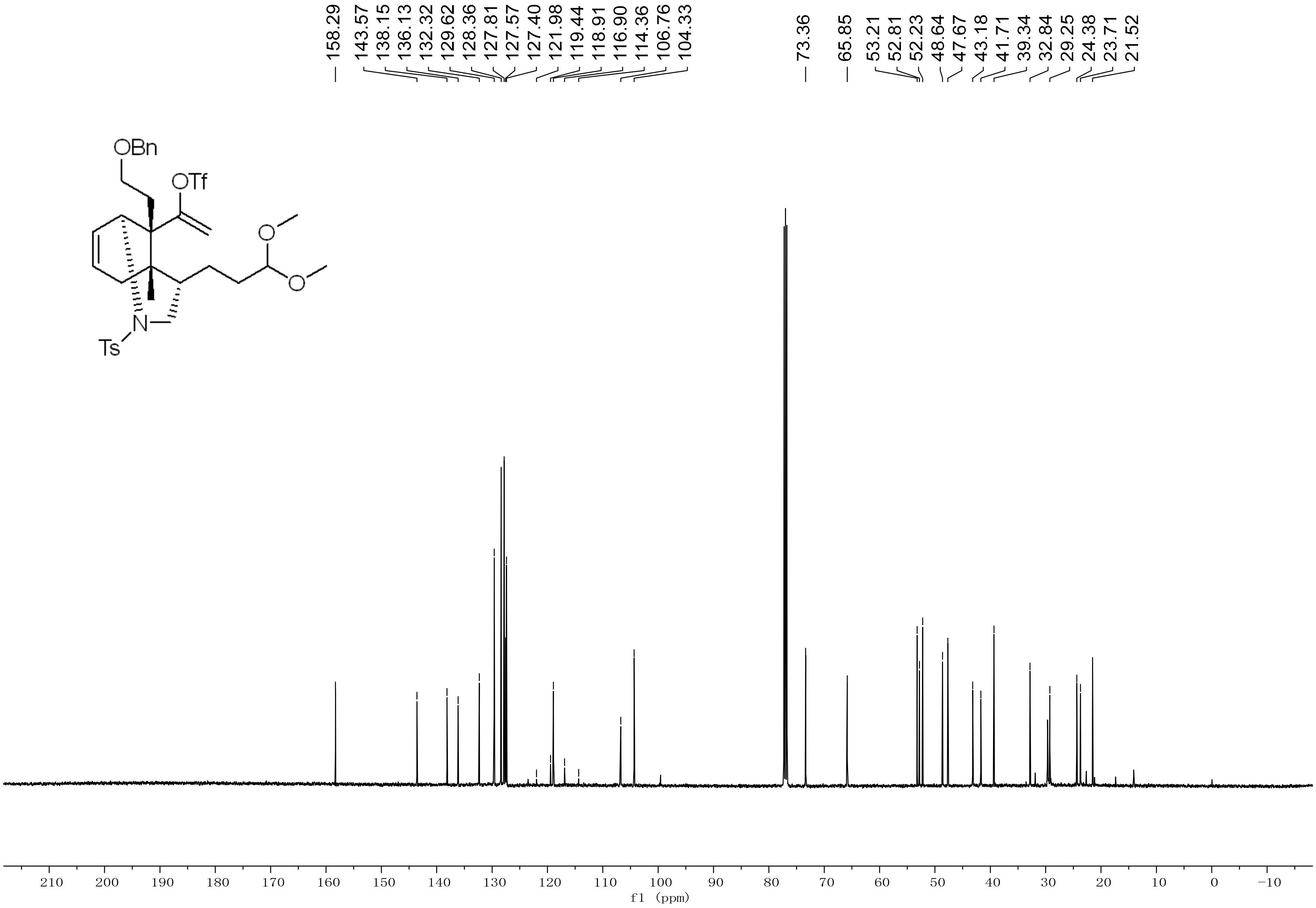


**Supplementary Figure 8.** **$^{13}$C-NMR of compound S1**

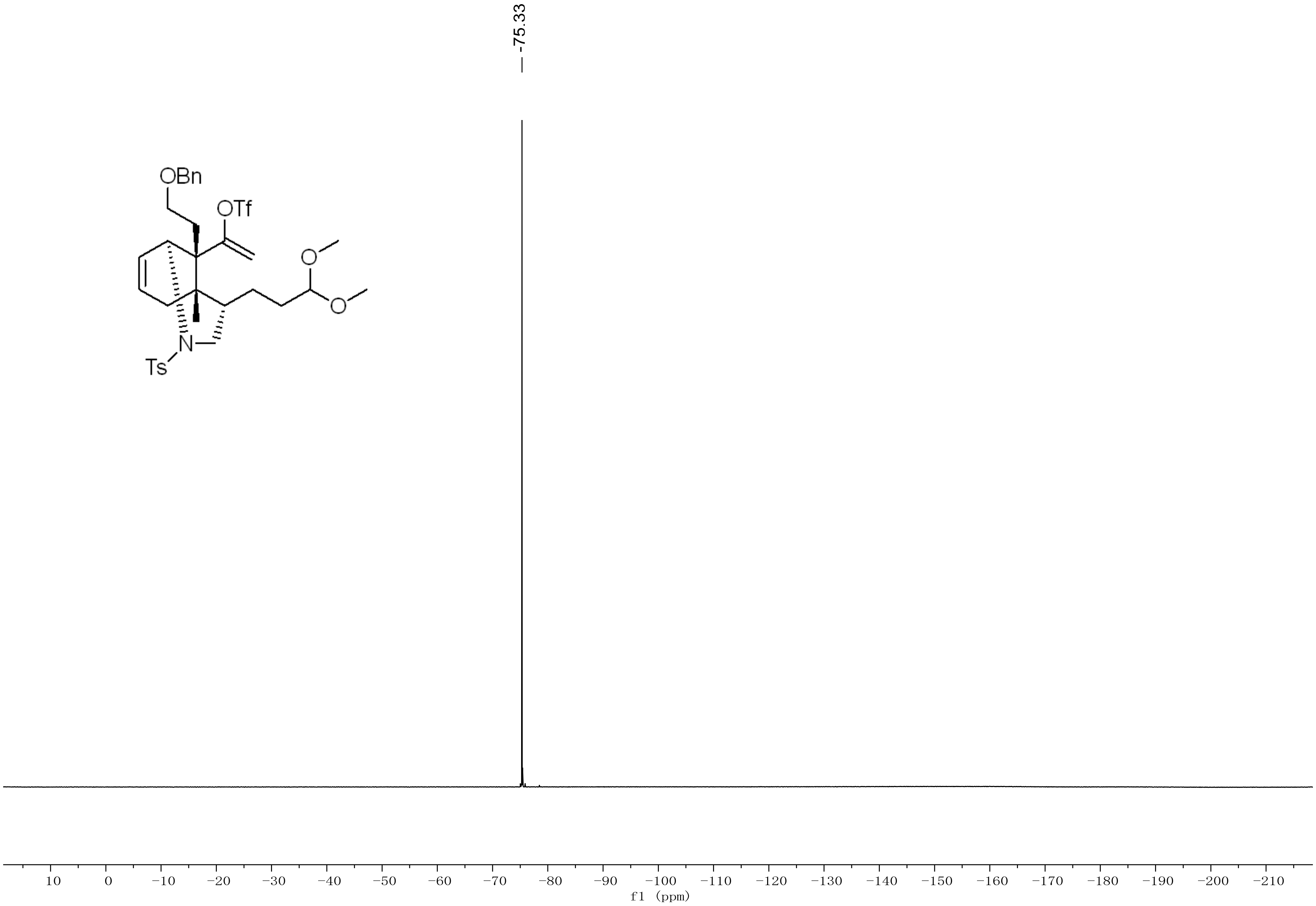


**Supplementary Figure 9.** $^{19}$**F-NMR of compound S1**

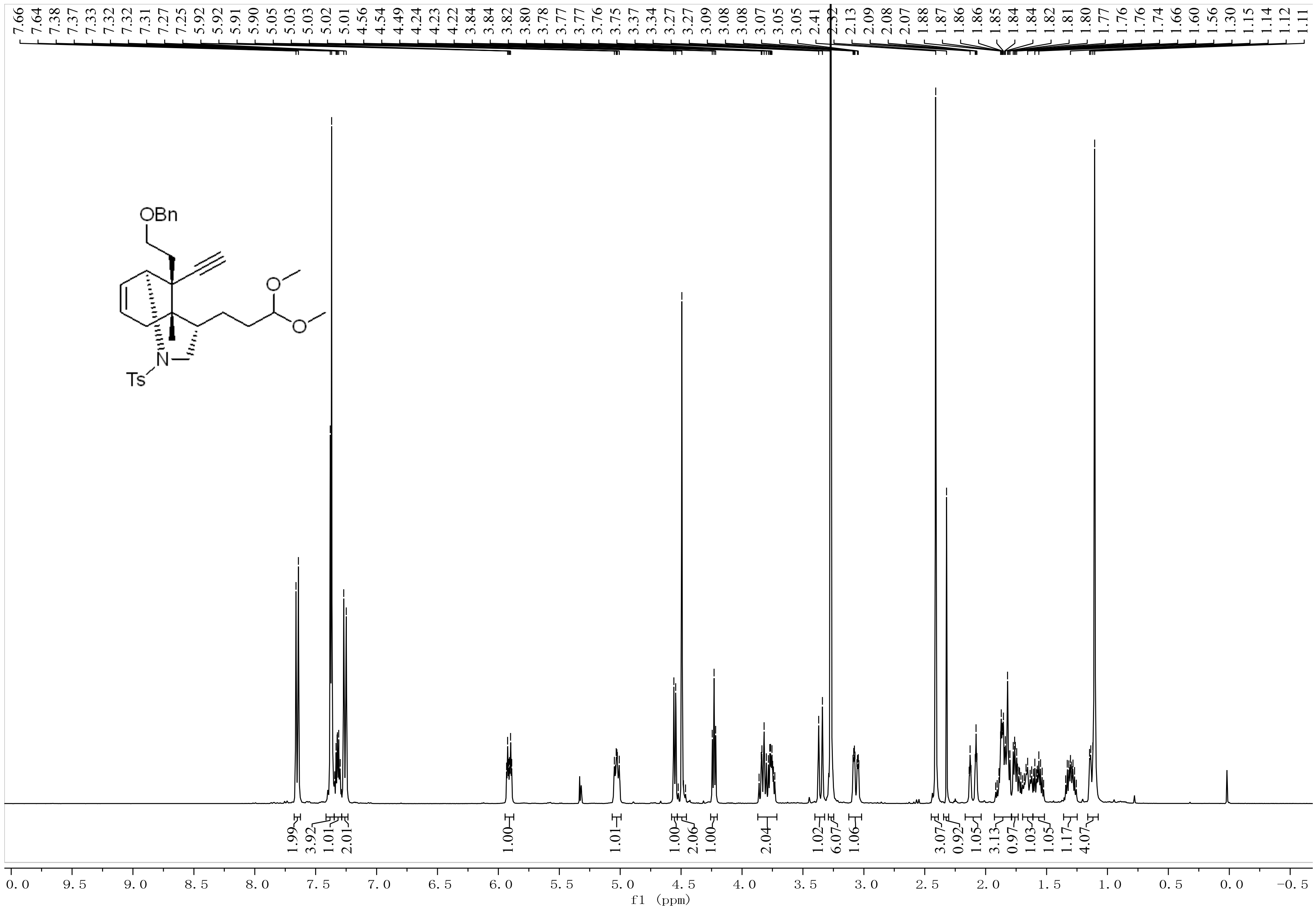


Supplementary Figure 10. $^{1}$H-NMR of compound 7

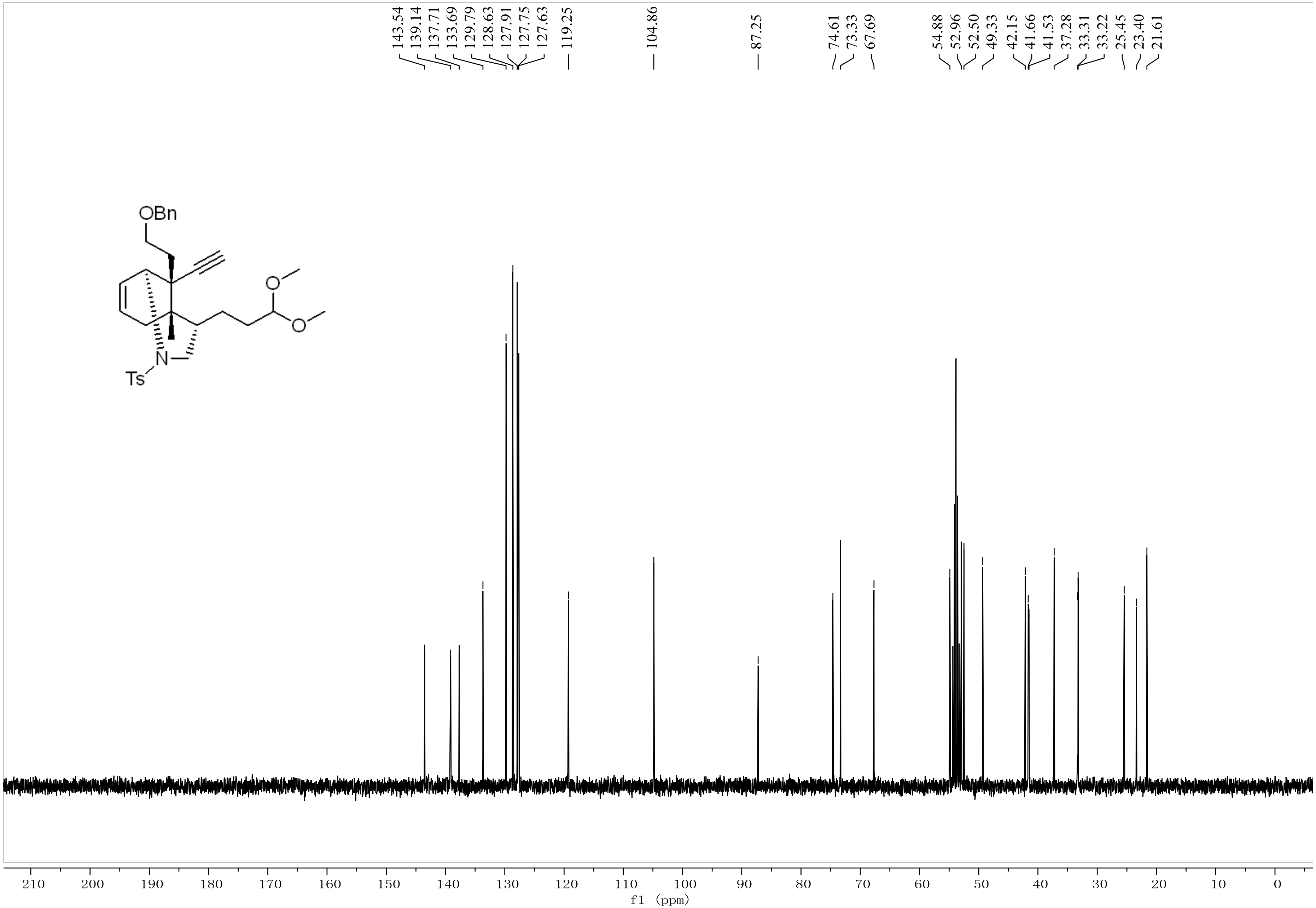


**Supplementary Figure 11.** **$^{13}$C-NMR of compound 7**

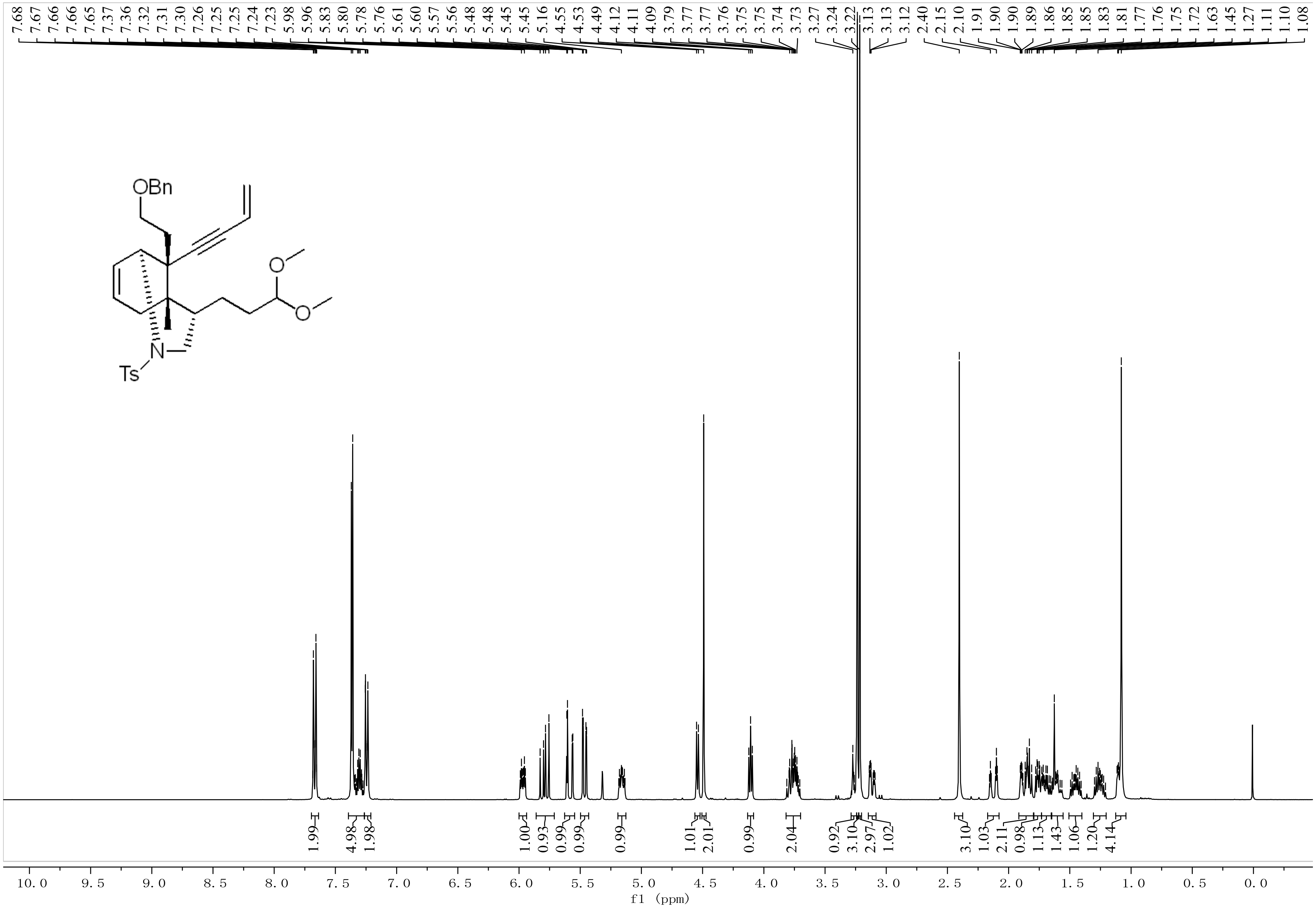

**Supplementary Figure 12.** [1]**H-NMR of compound 3**

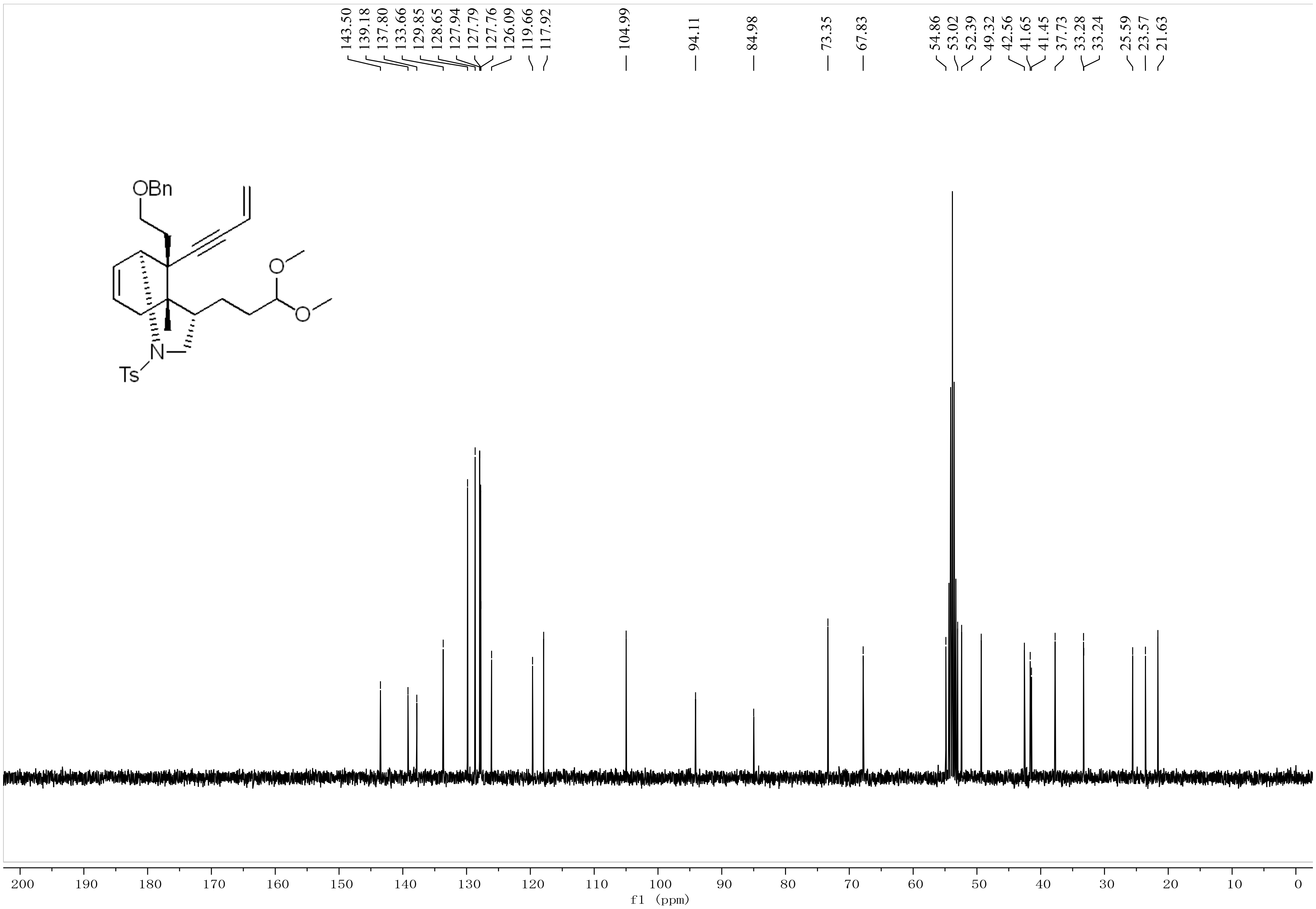


**Supplementary Figure 13.** $^{13}$**C-NMR of compound 3**

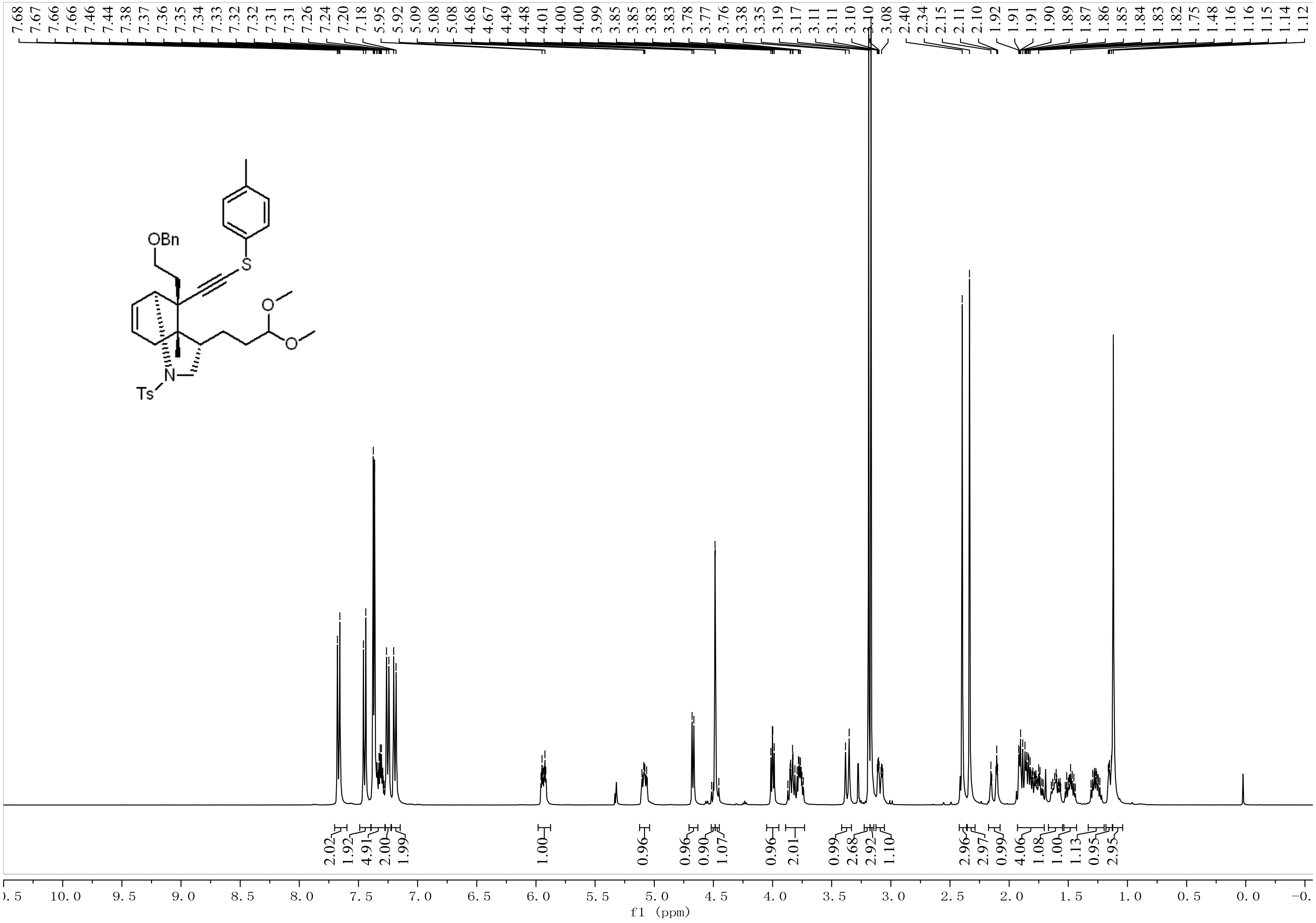

**Supplementary Figure 14.** $^1$H-NMR of compound 9

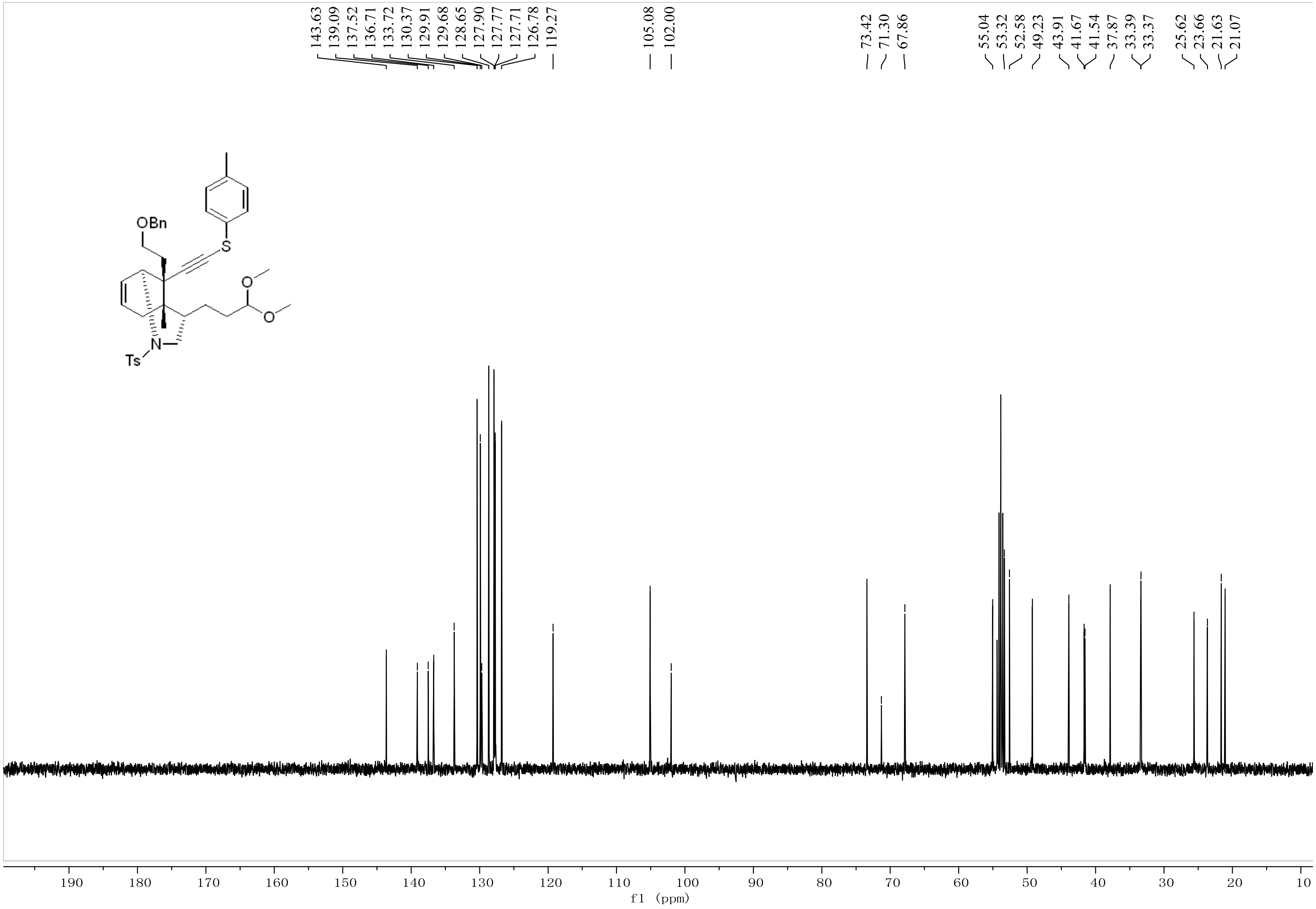


**Supplementary Figure 15.** **$^{13}$C-NMR of compound 9**

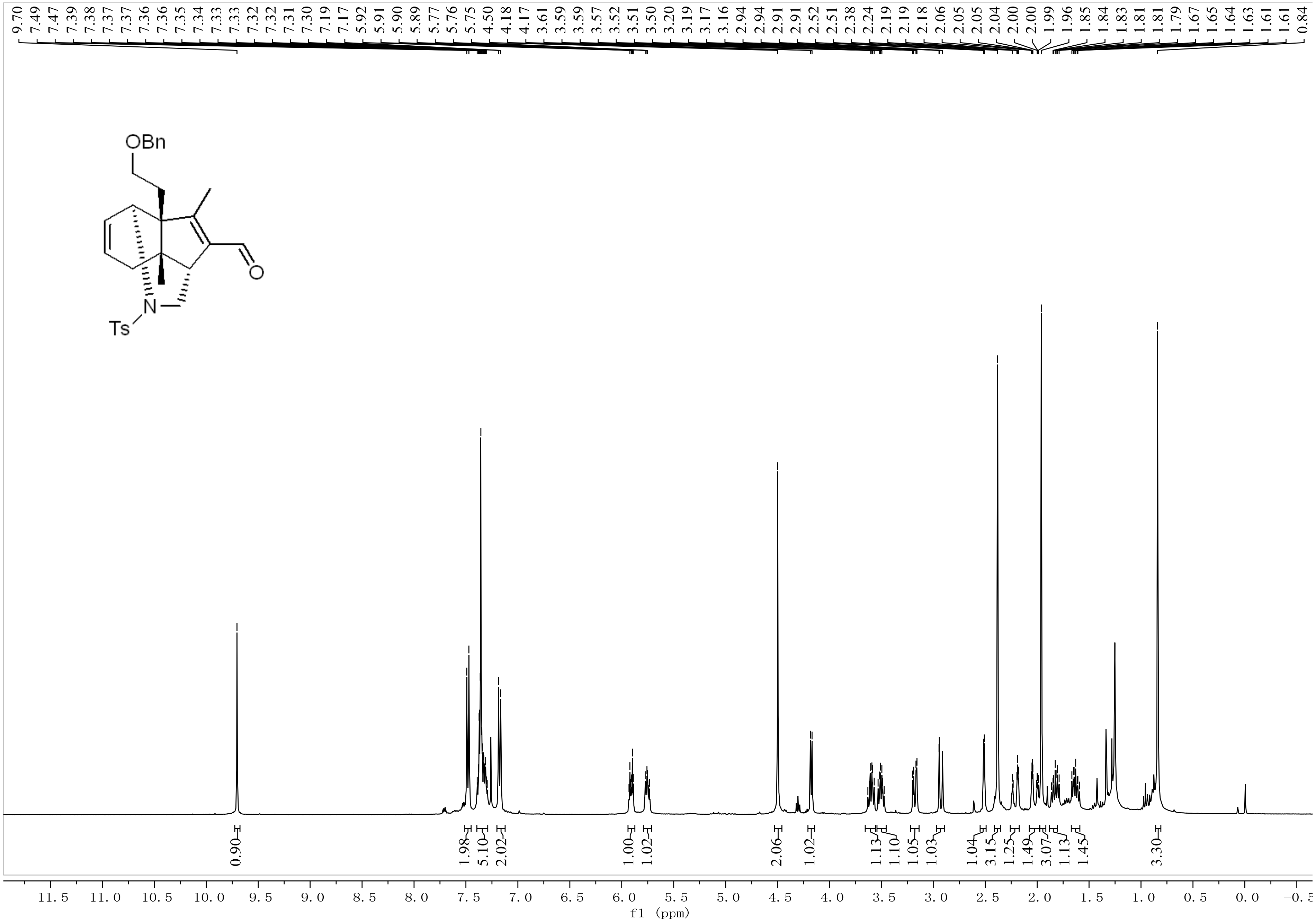

**Supplementary Figure 16.** **$^{1}$H-NMR of compound 11**

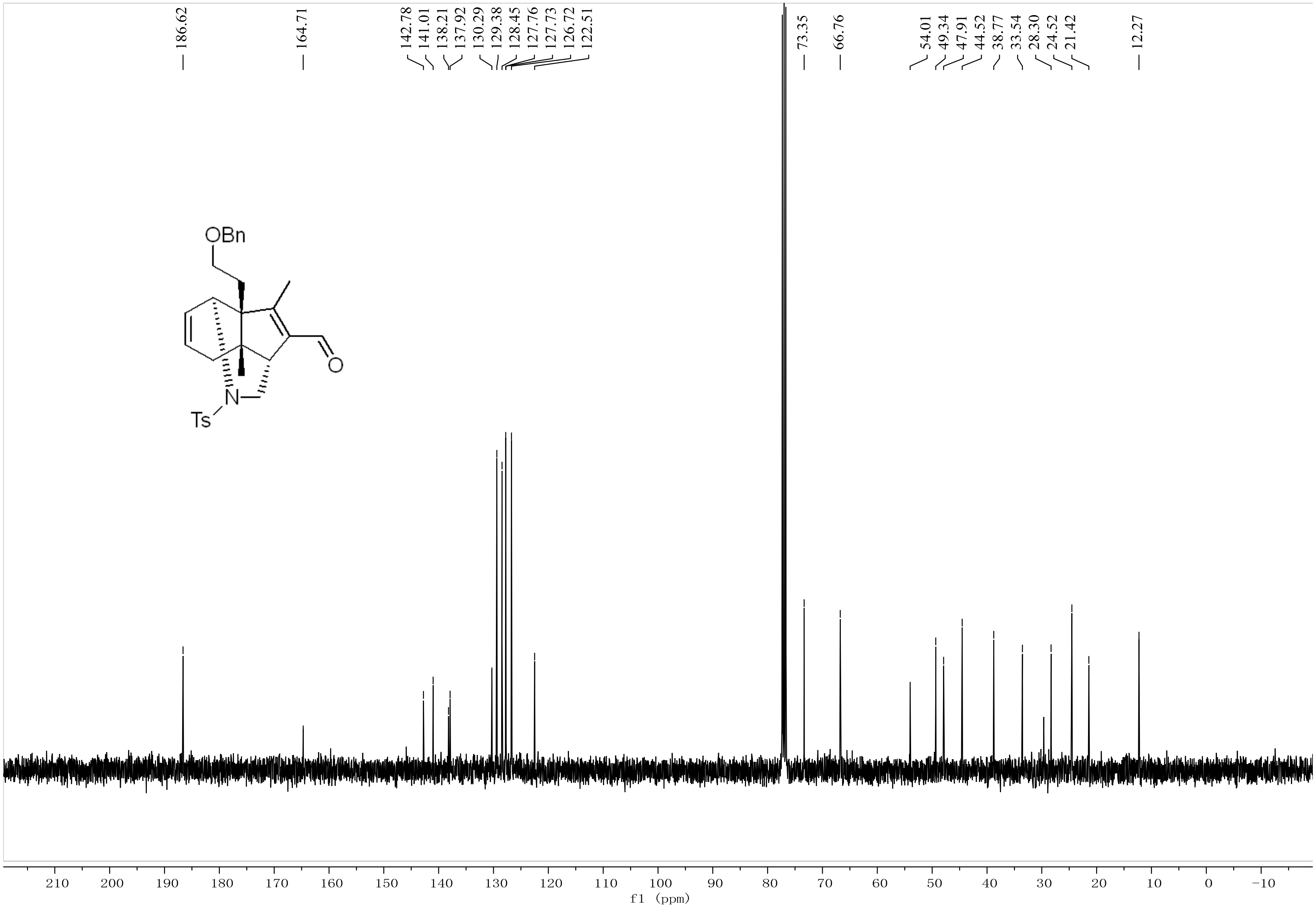


**Supplementary Figure 17.** $^{13}$**C-NMR of compound 11**

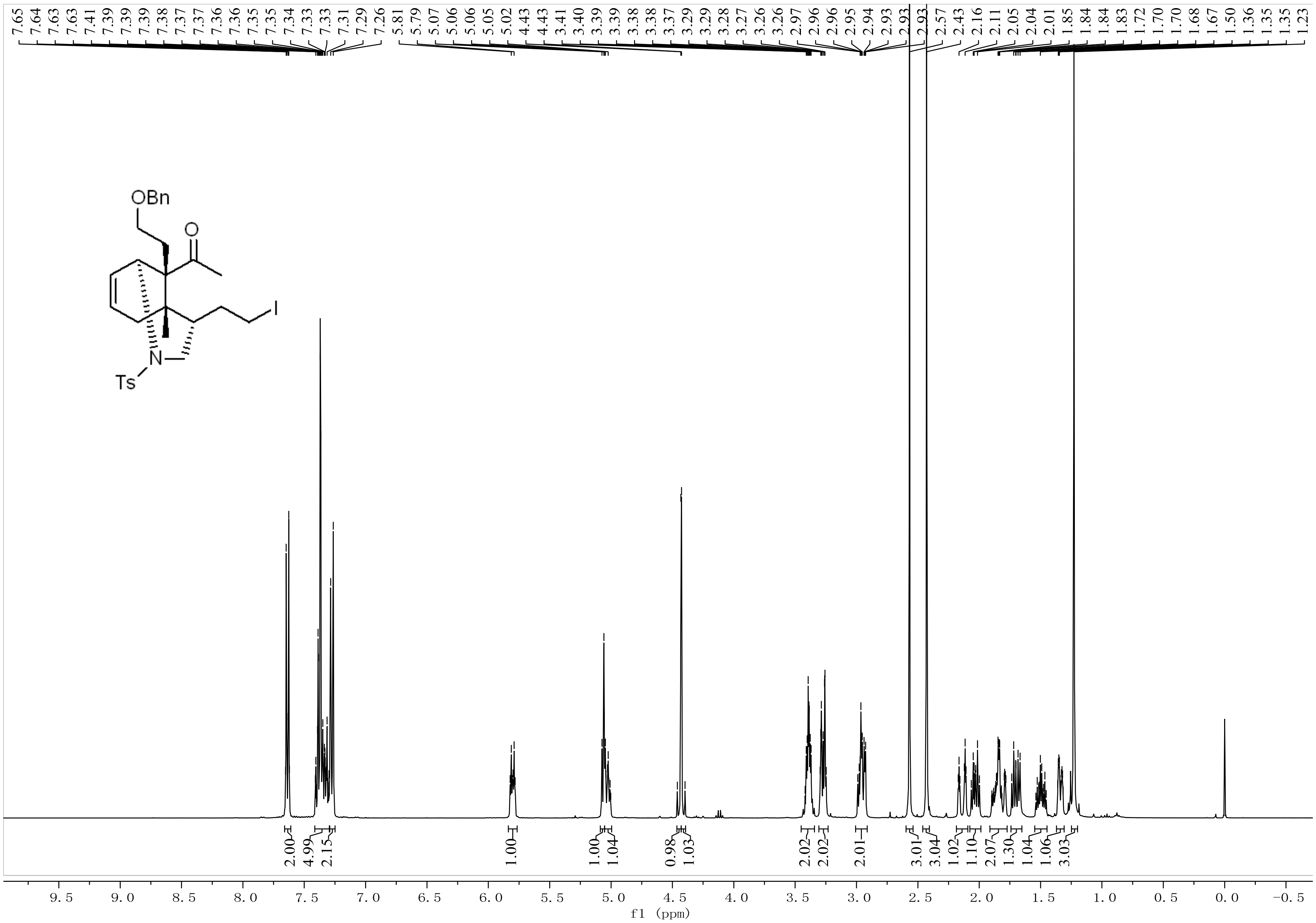

**Supplementary Figure 18.** [1]**H-NMR of compound S2**

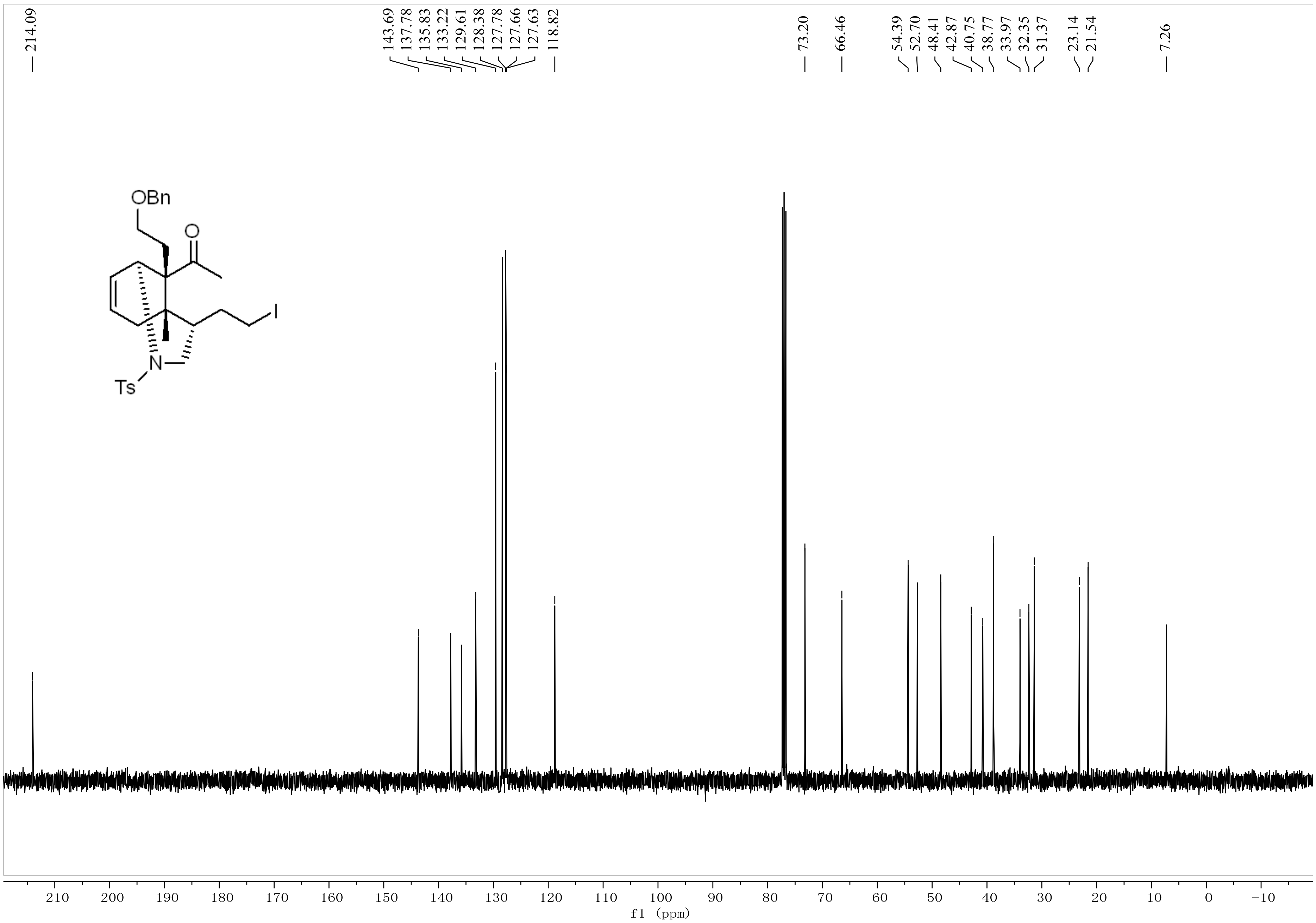


**Supplementary Figure 19.** **$^{13}$C-NMR of compound S2**

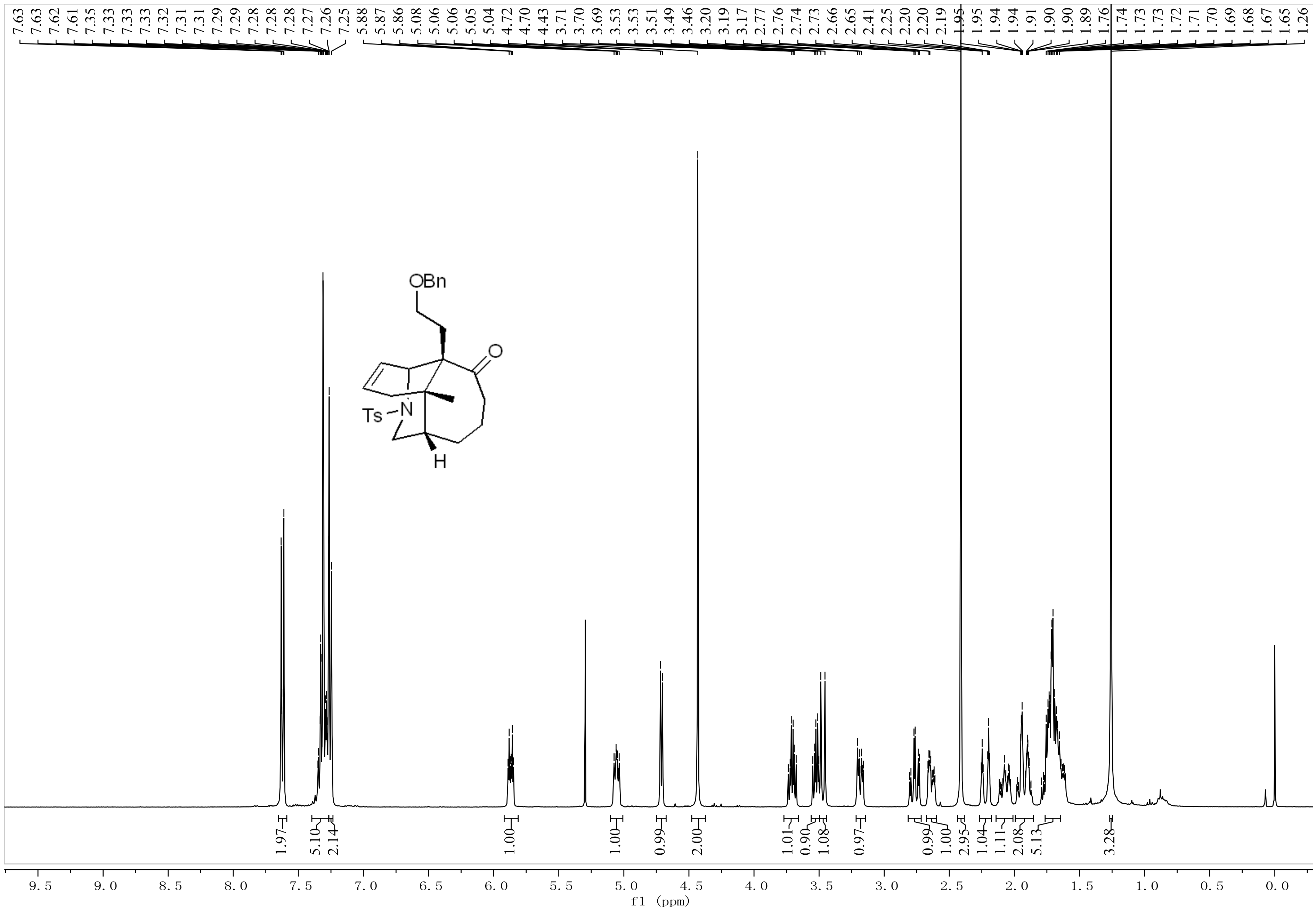

**Supplementary Figure 20.** $^{1}$H-NMR of compound 13

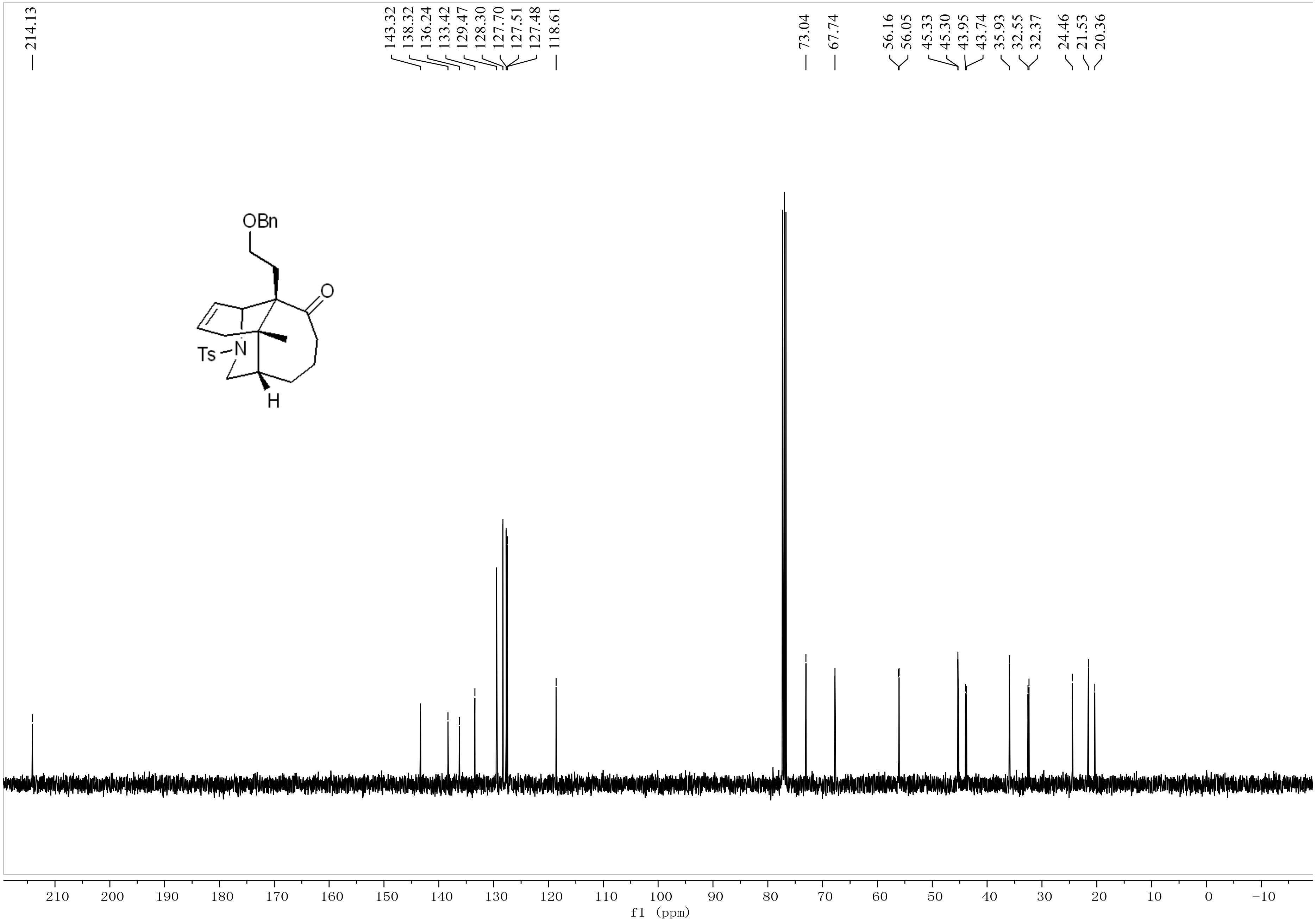


**Supplementary Figure 21.** **$^{13}$C-NMR of compound 13**

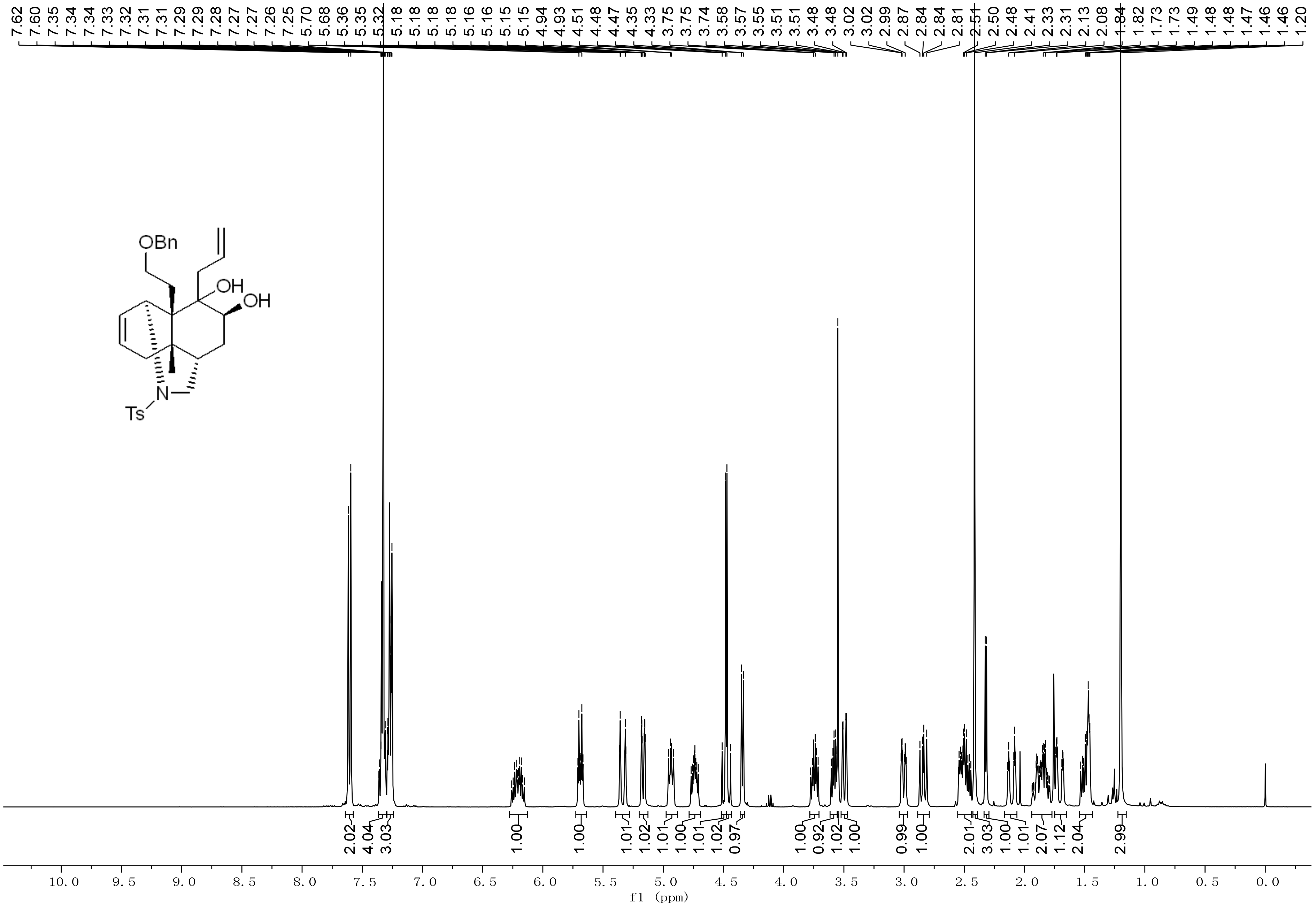

**Supplementary Figure 22.** **$^{1}$H-NMR of compound S3**

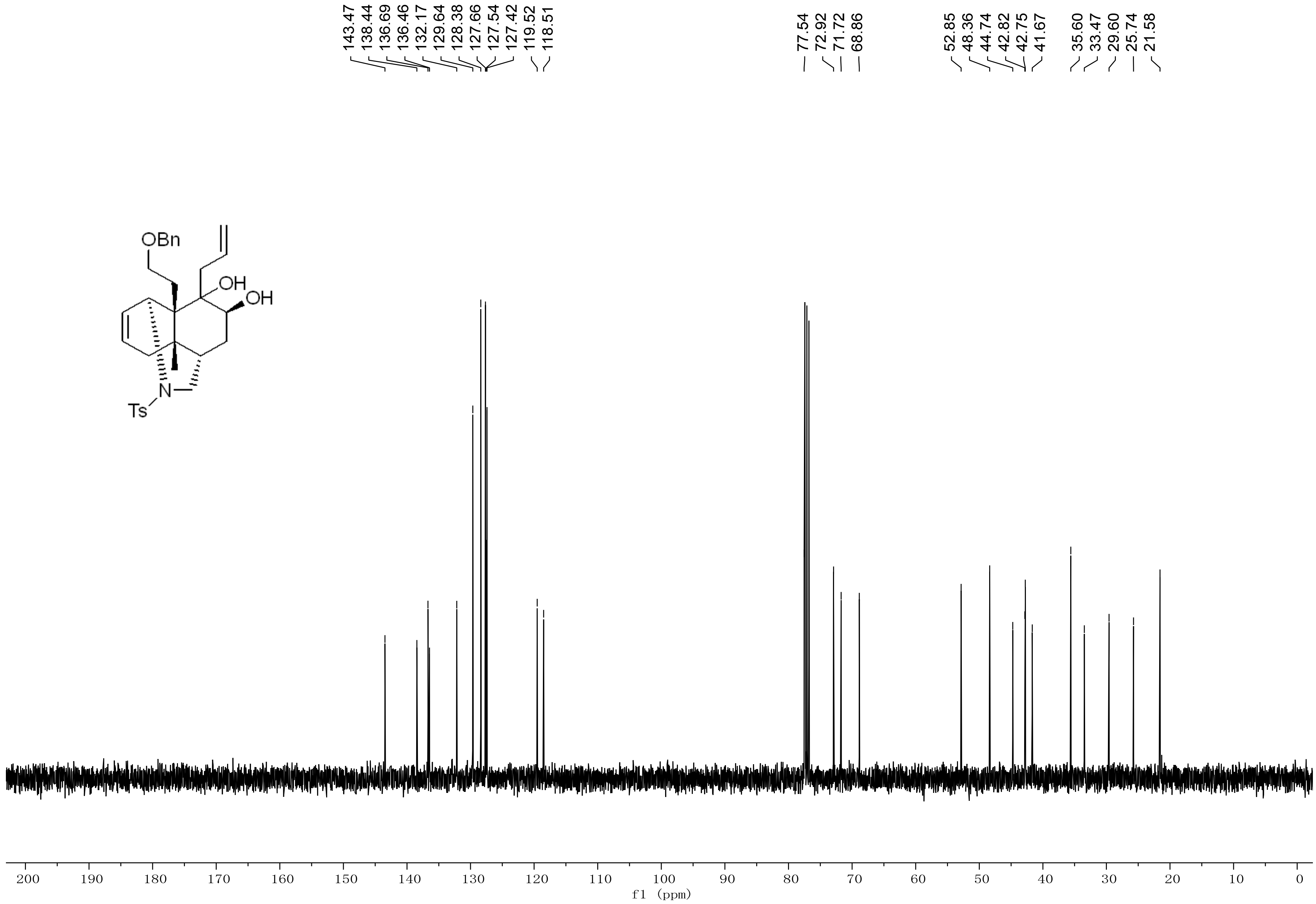


**Supplementary Figure 23.** **$^{13}$C-NMR of compound S3**

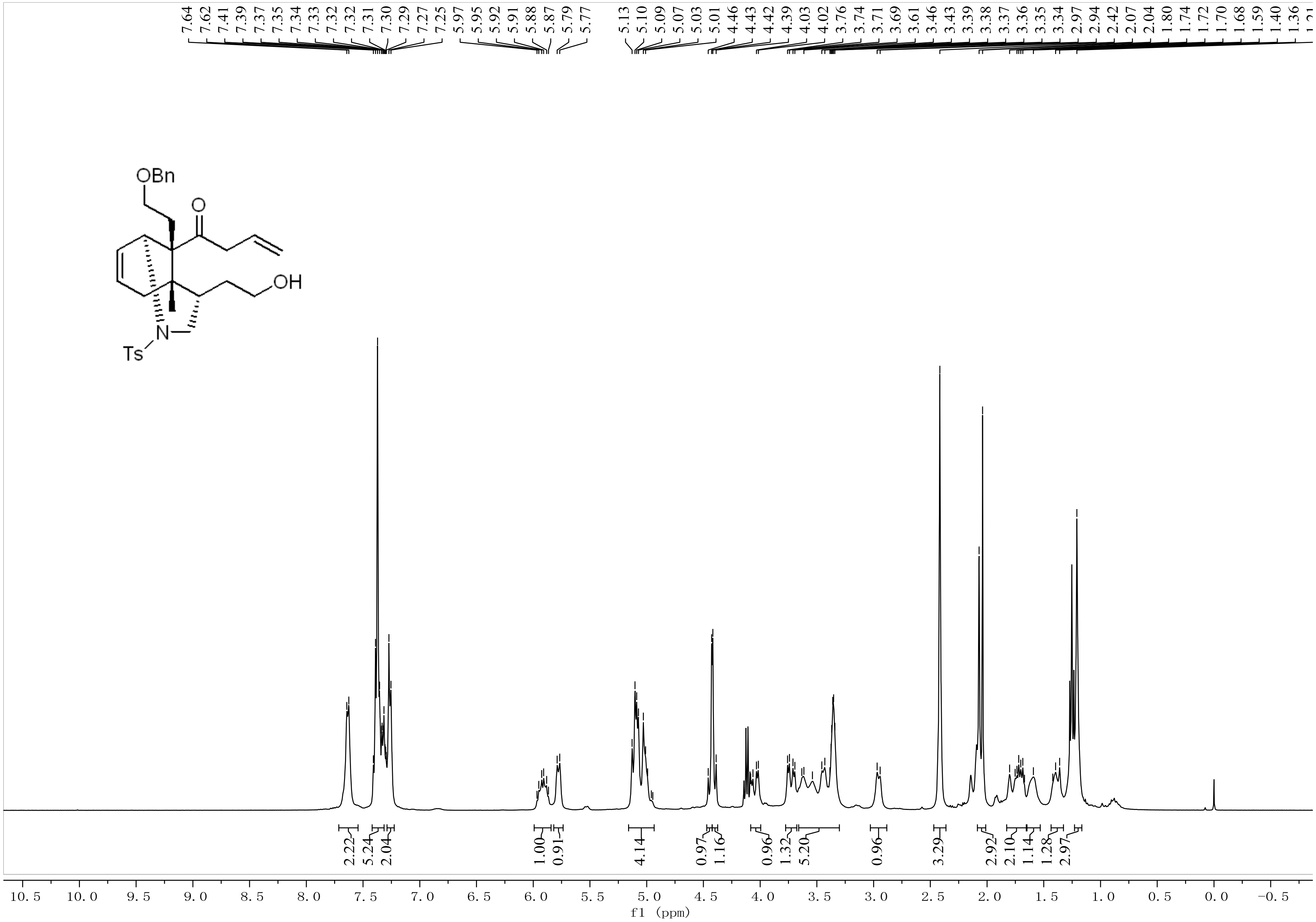

**Supplementary Figure 24. $^{1}$H-NMR of compound 16**

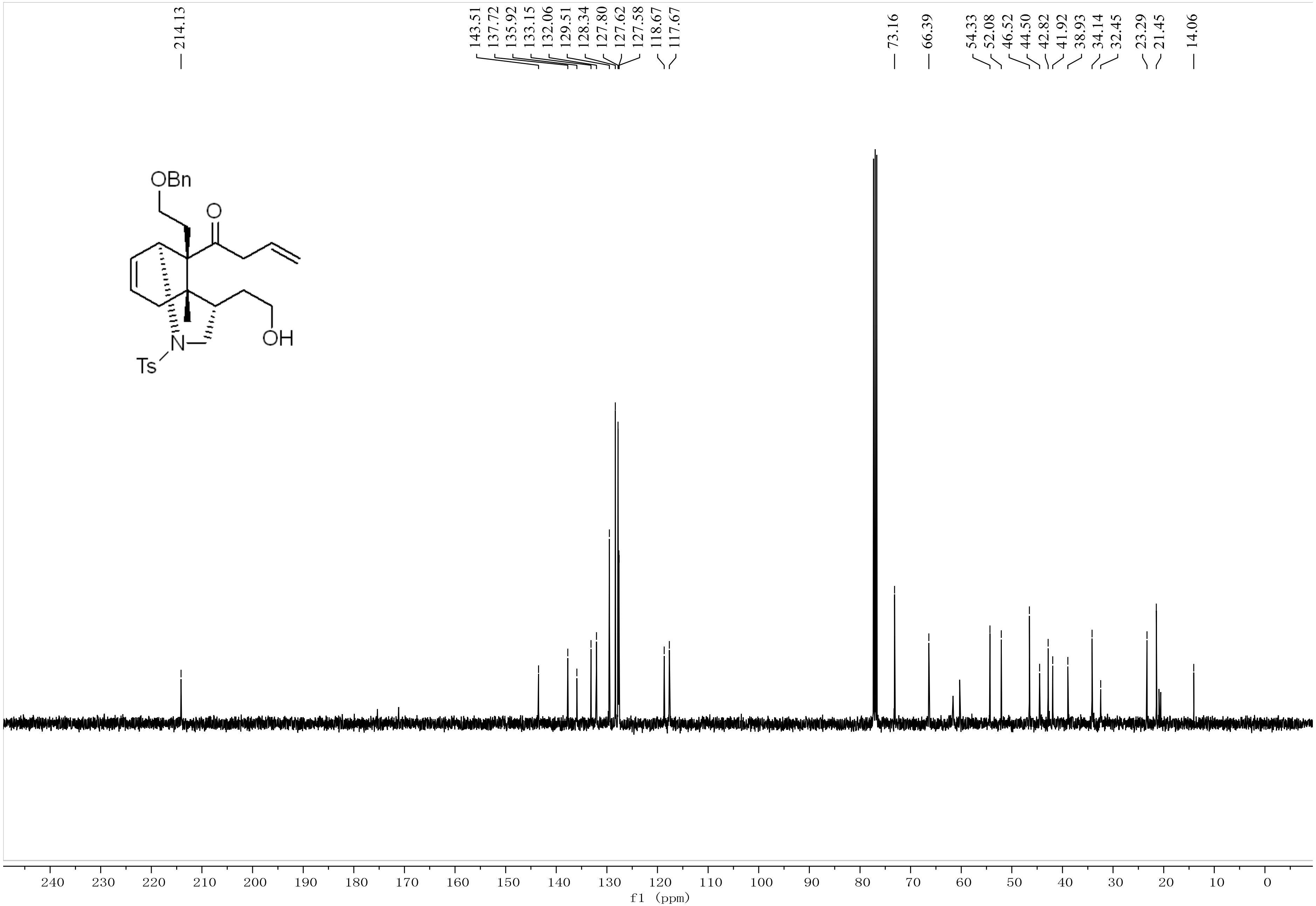


**Supplementary Figure 25.** $^{1}$H-NMR of compound 16

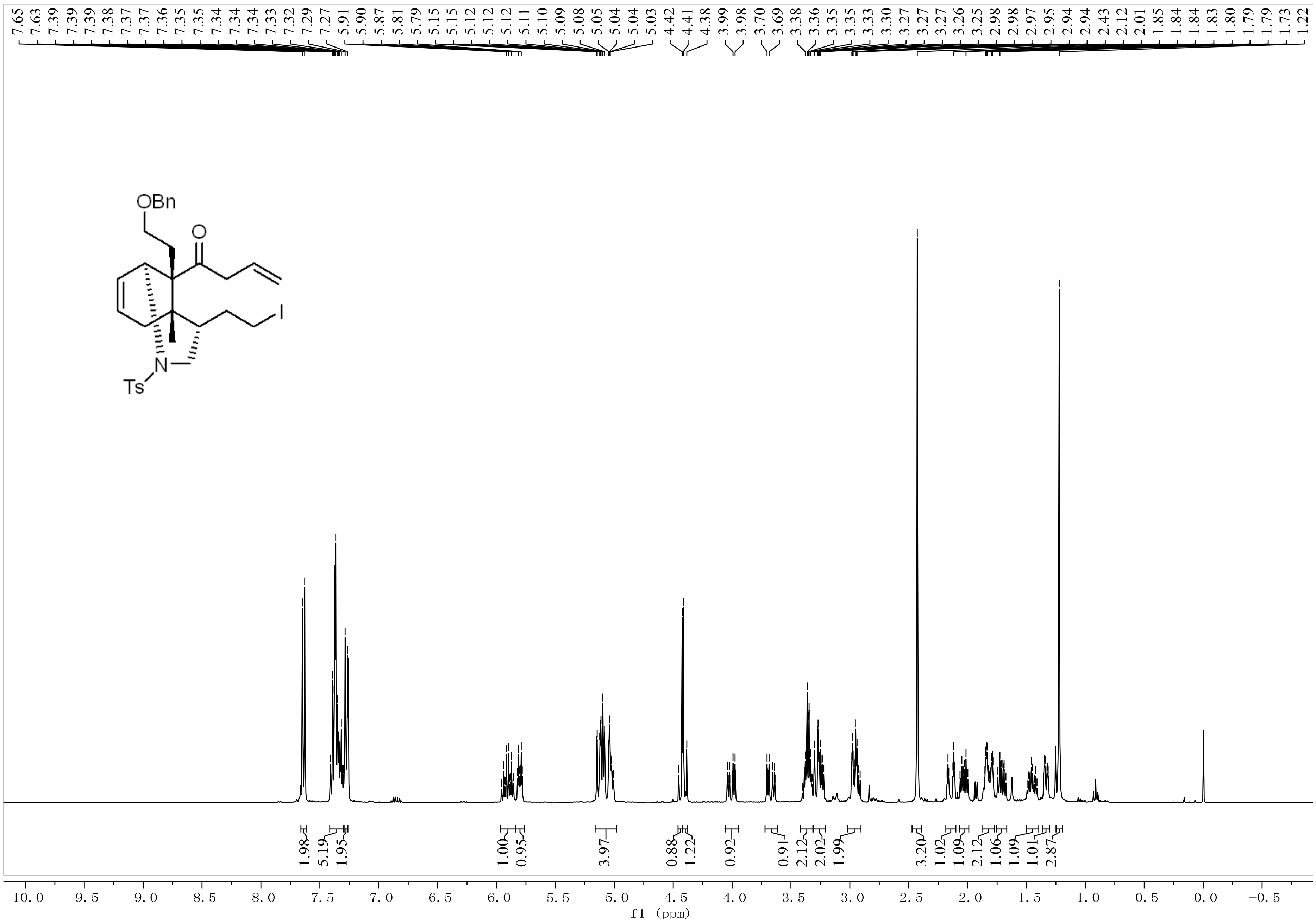

**Supplementary Figure 26.** **$^{1}$H-NMR of compound 17**

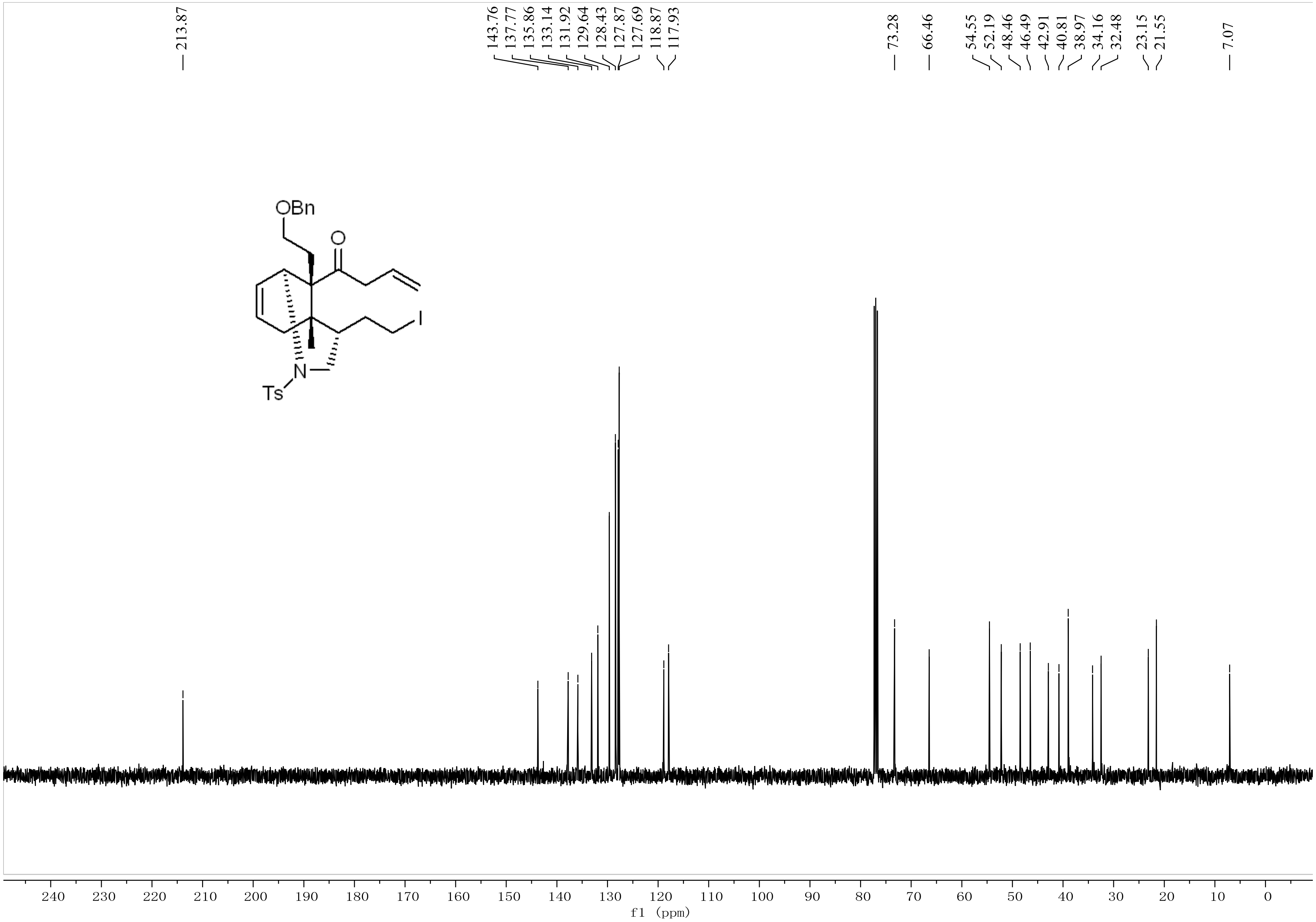


**Supplementary Figure 27.** $^{1}$**H-NMR of compound 17**

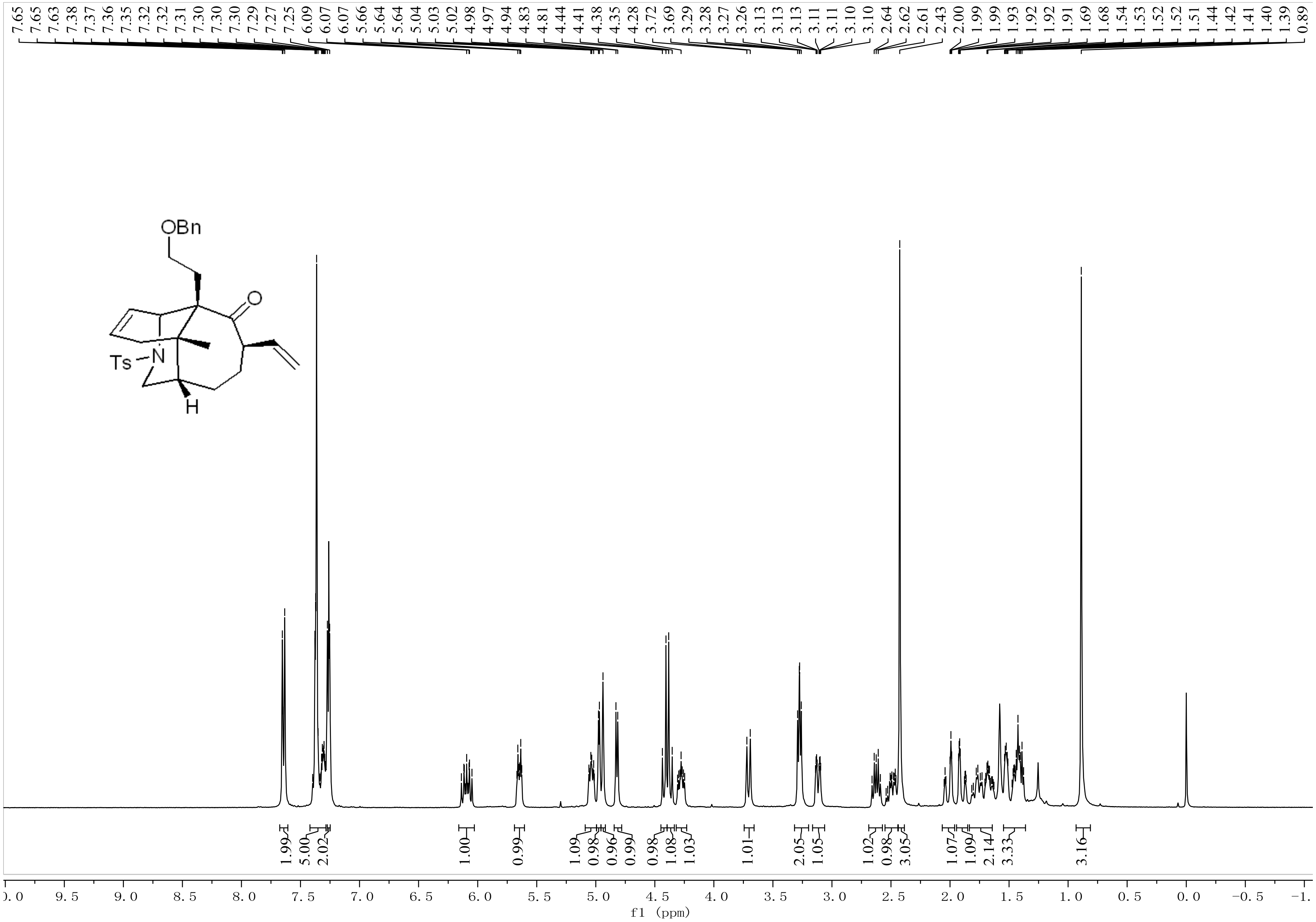

**Supplementary Figure 28.** [1]**H-NMR of compound 18a**

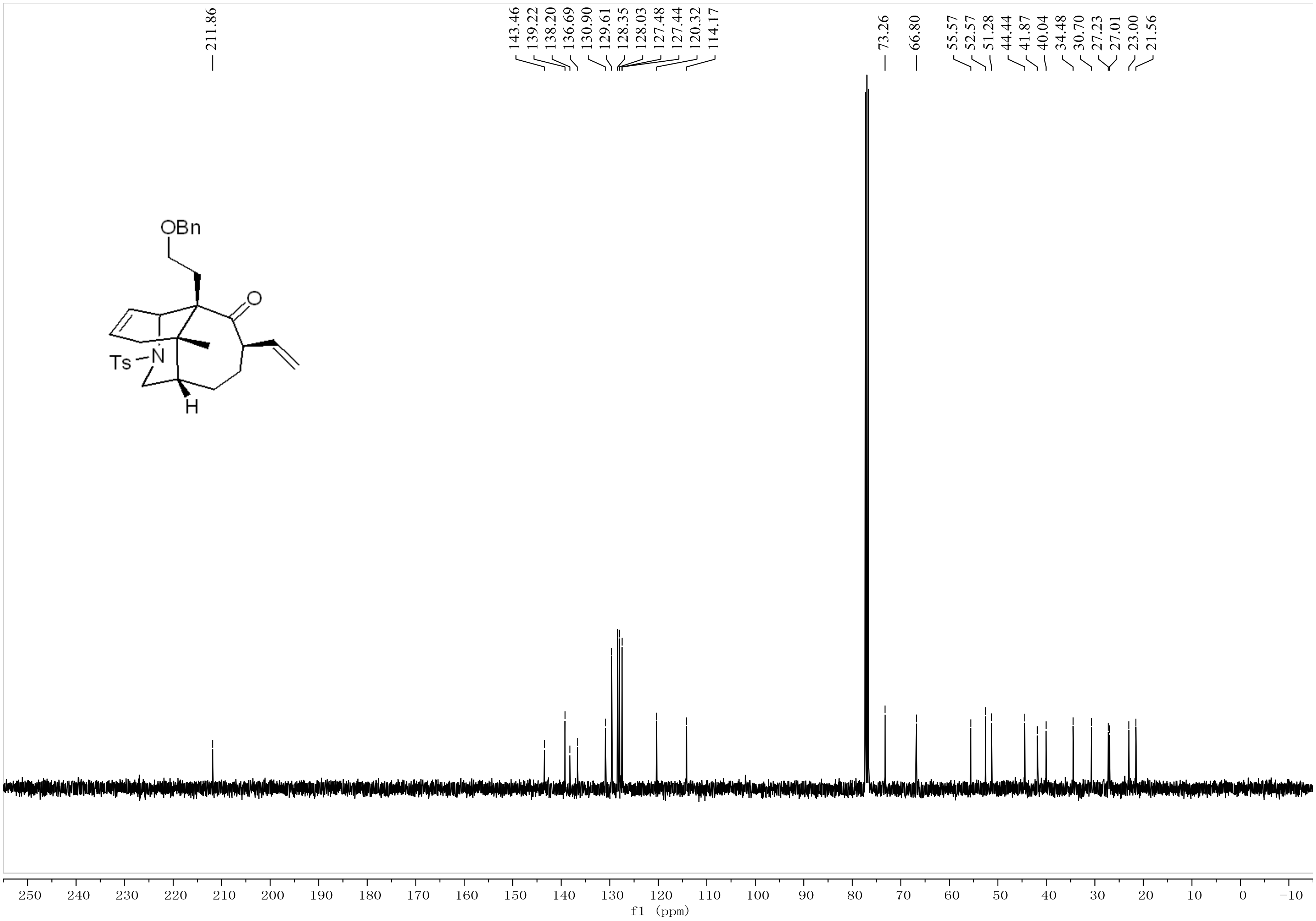


**Supplementary Figure 29.** $^{13}$**C-NMR of compound 18a**

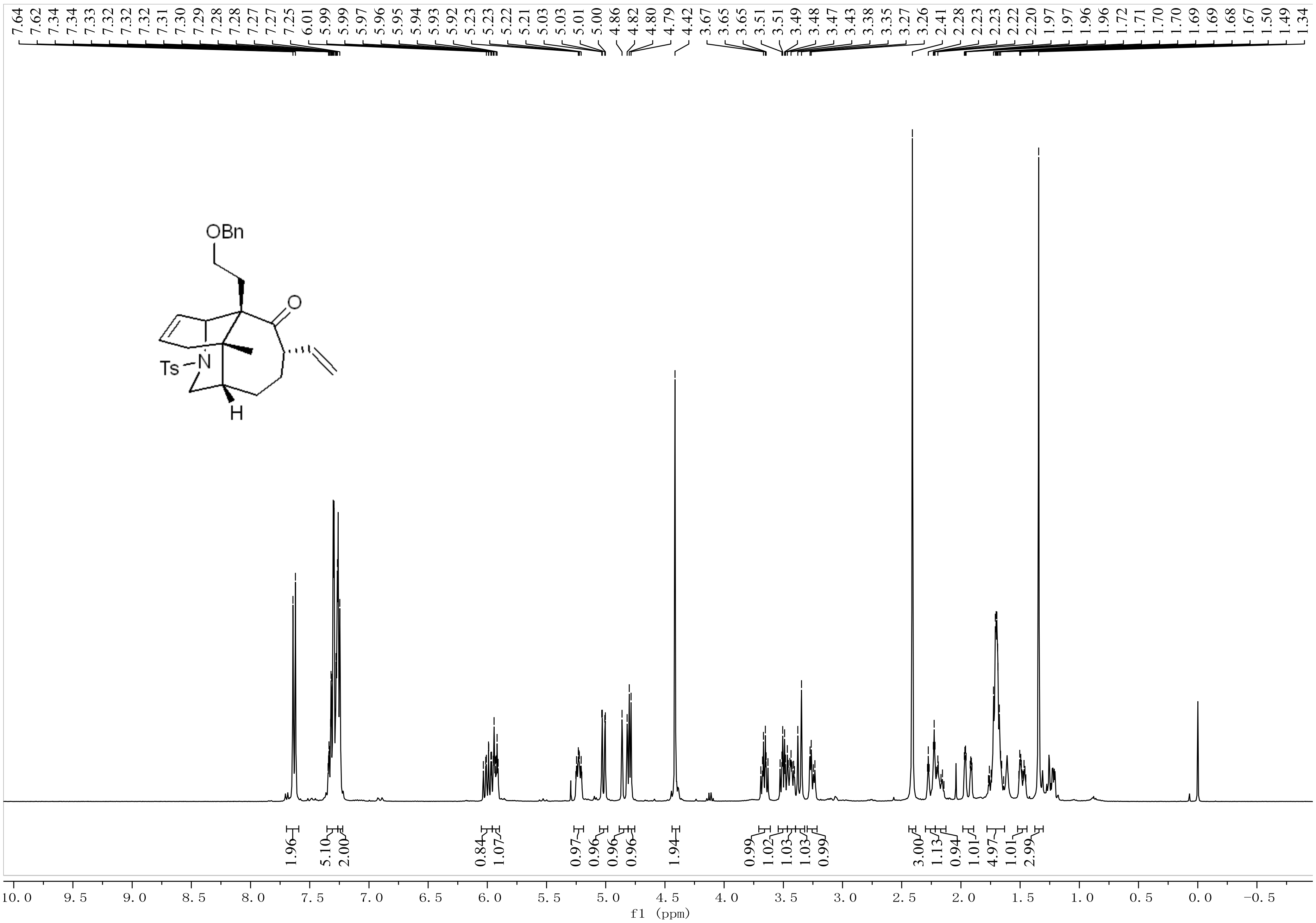

**Supplementary Figure 30.** [1]**H-NMR of compound 18b**

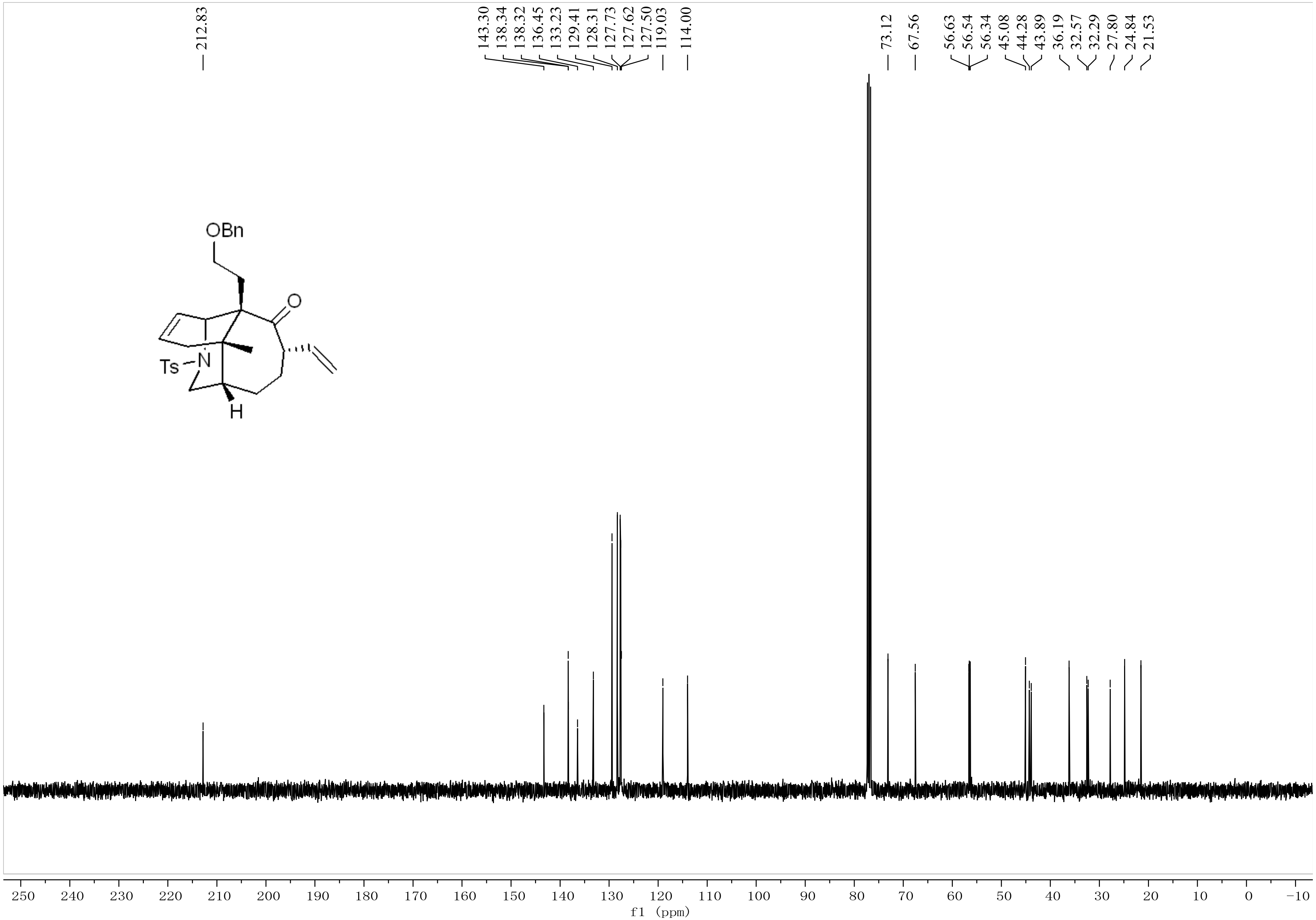


**Supplementary Figure 31.** **$^{13}$C-NMR of compound 18b**

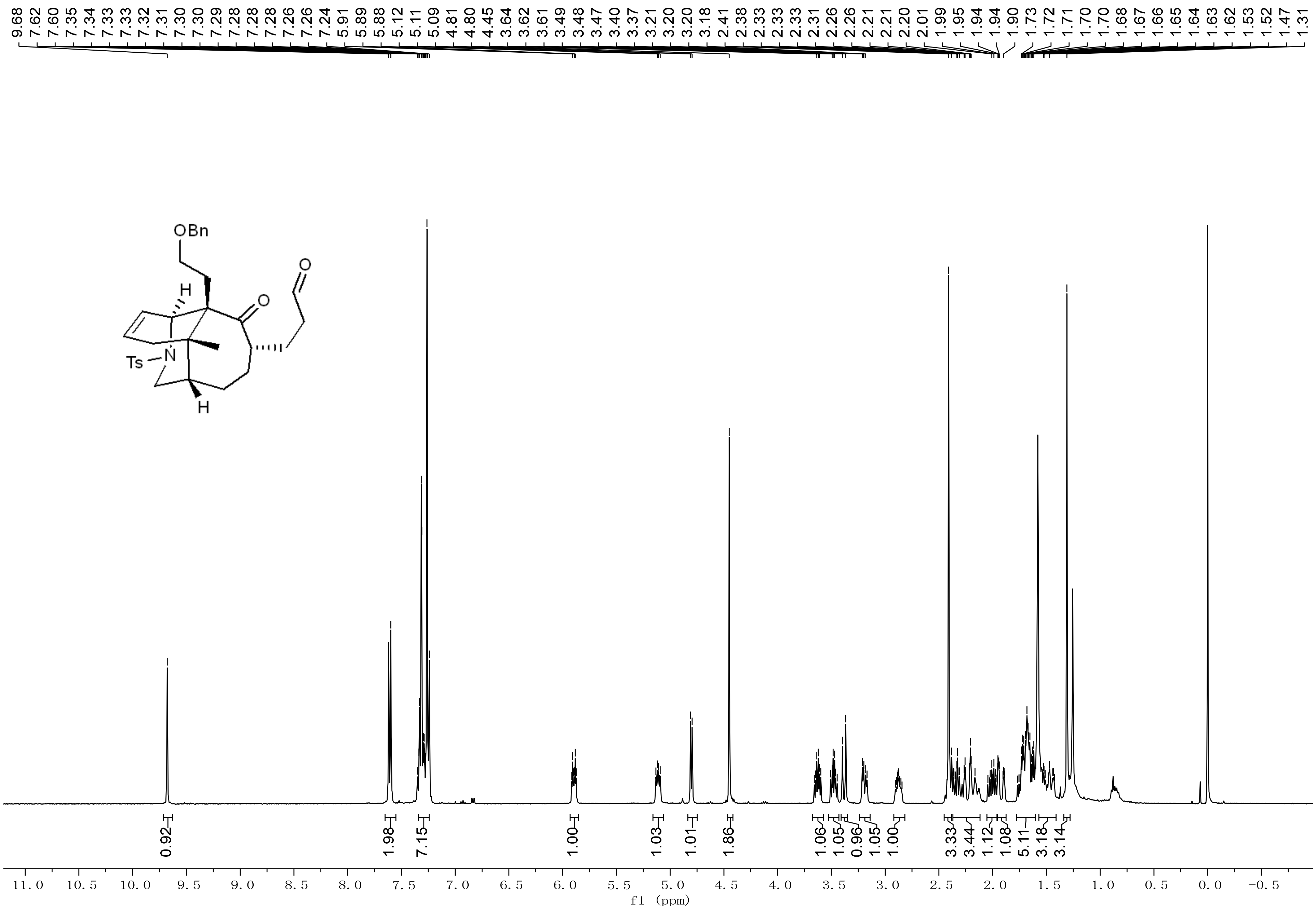

**Supplementary Figure 32.** [1]**H-NMR of compound 19**

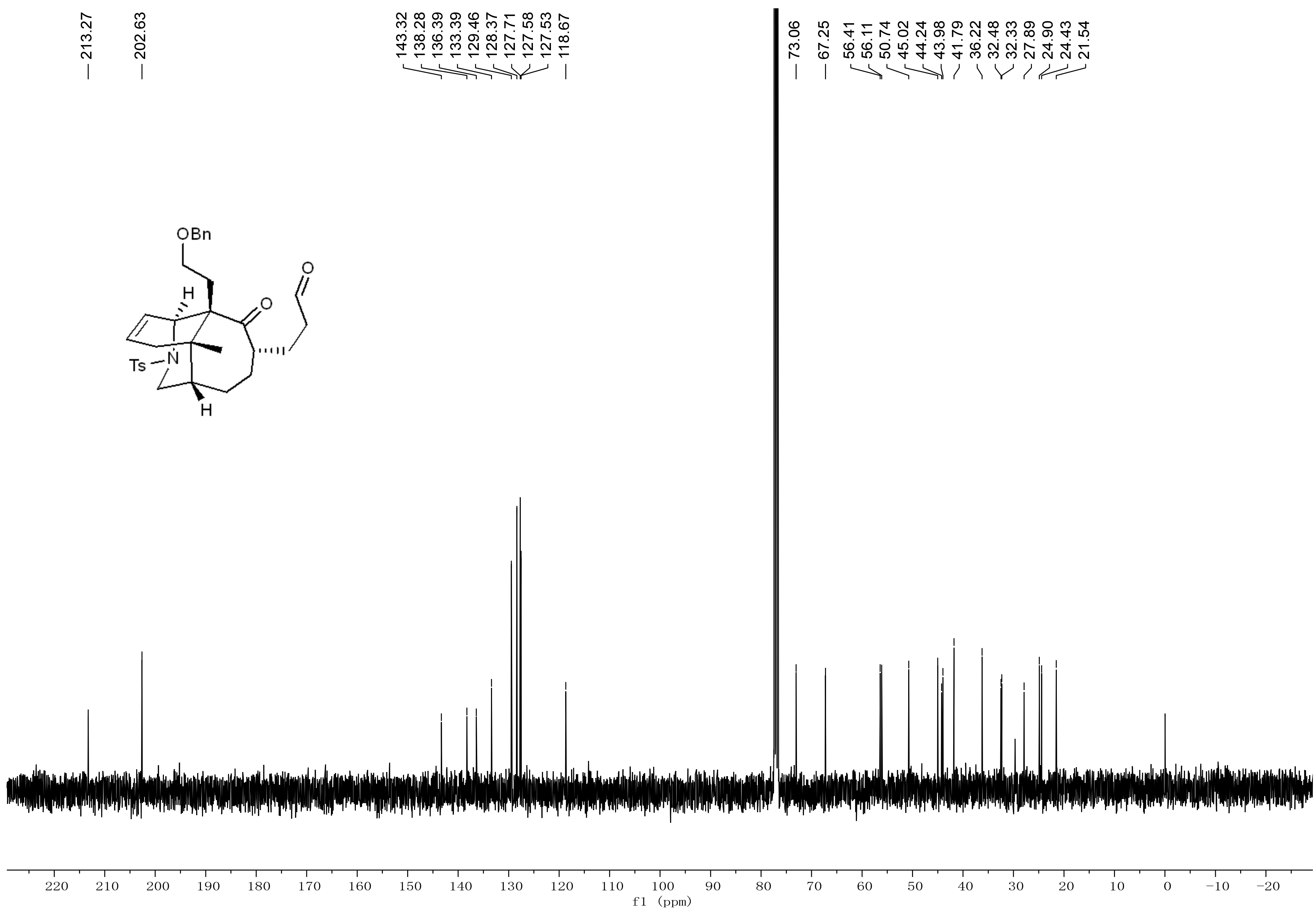


**Supplementary Figure 33.** **$^{13}$C-NMR of compound 19**

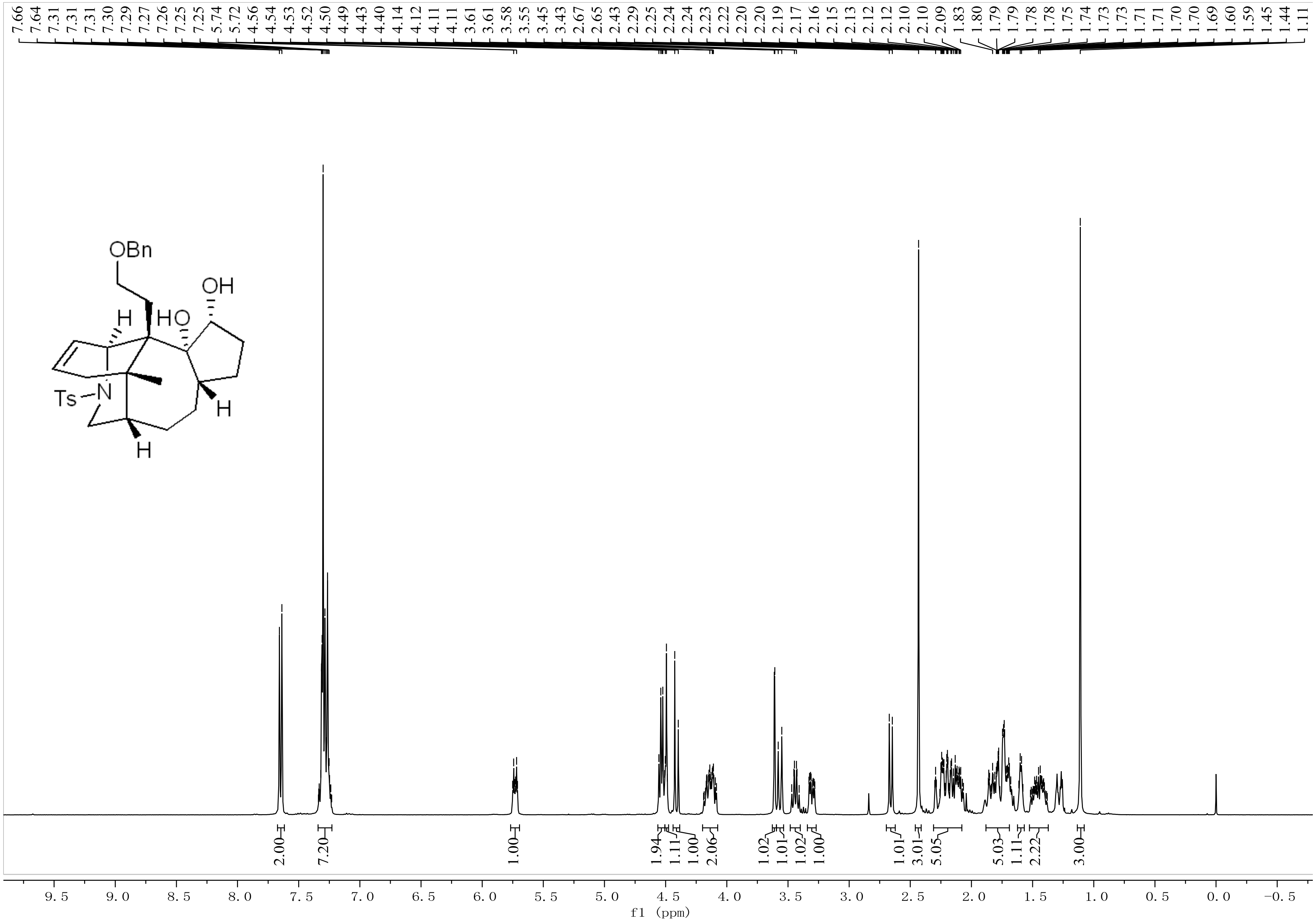

**Supplementary Figure 34.** [1]**H-NMR of compound 20**

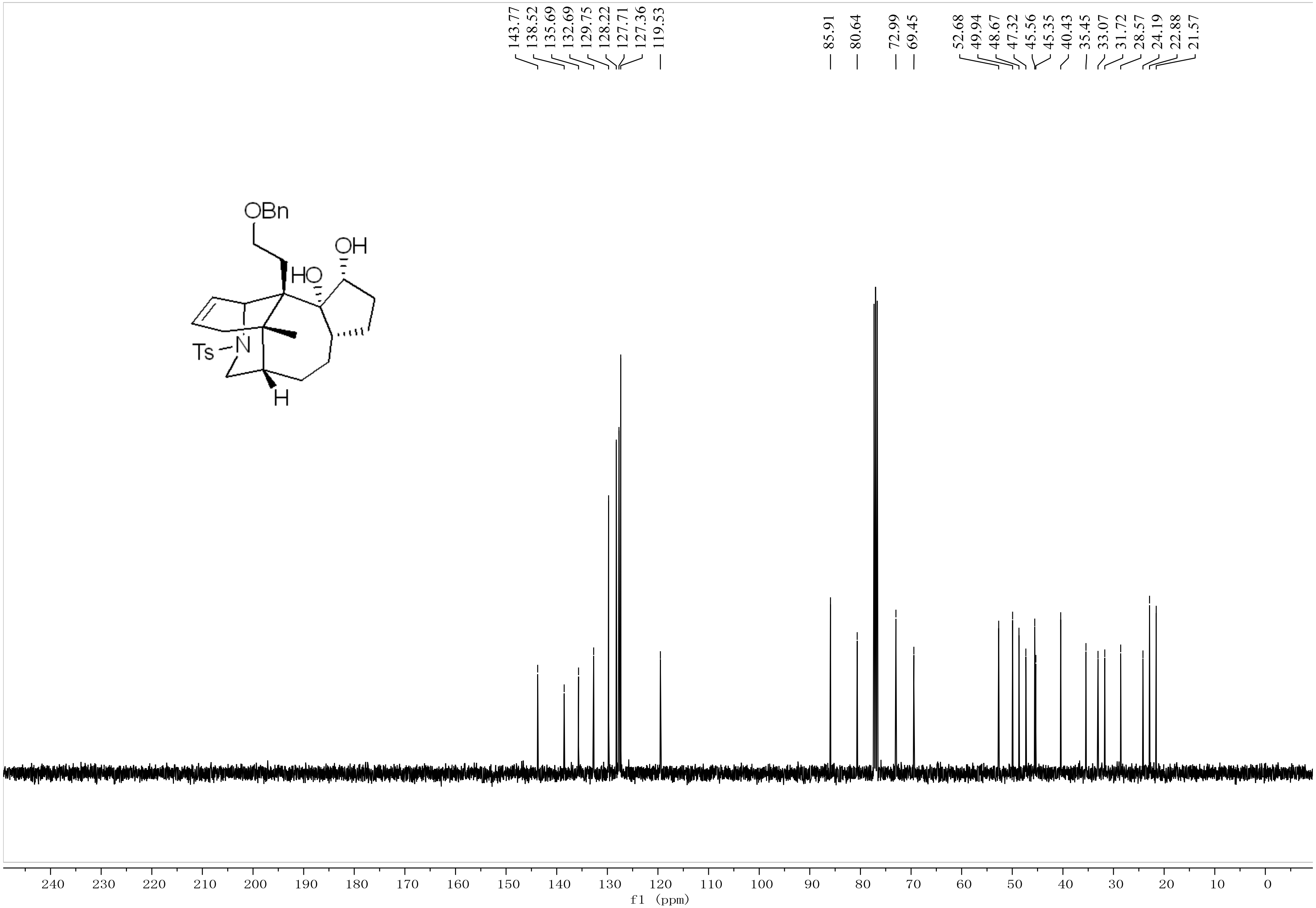


**Supplementary Figure 35.** $^{13}$**C-NMR of compound 20**

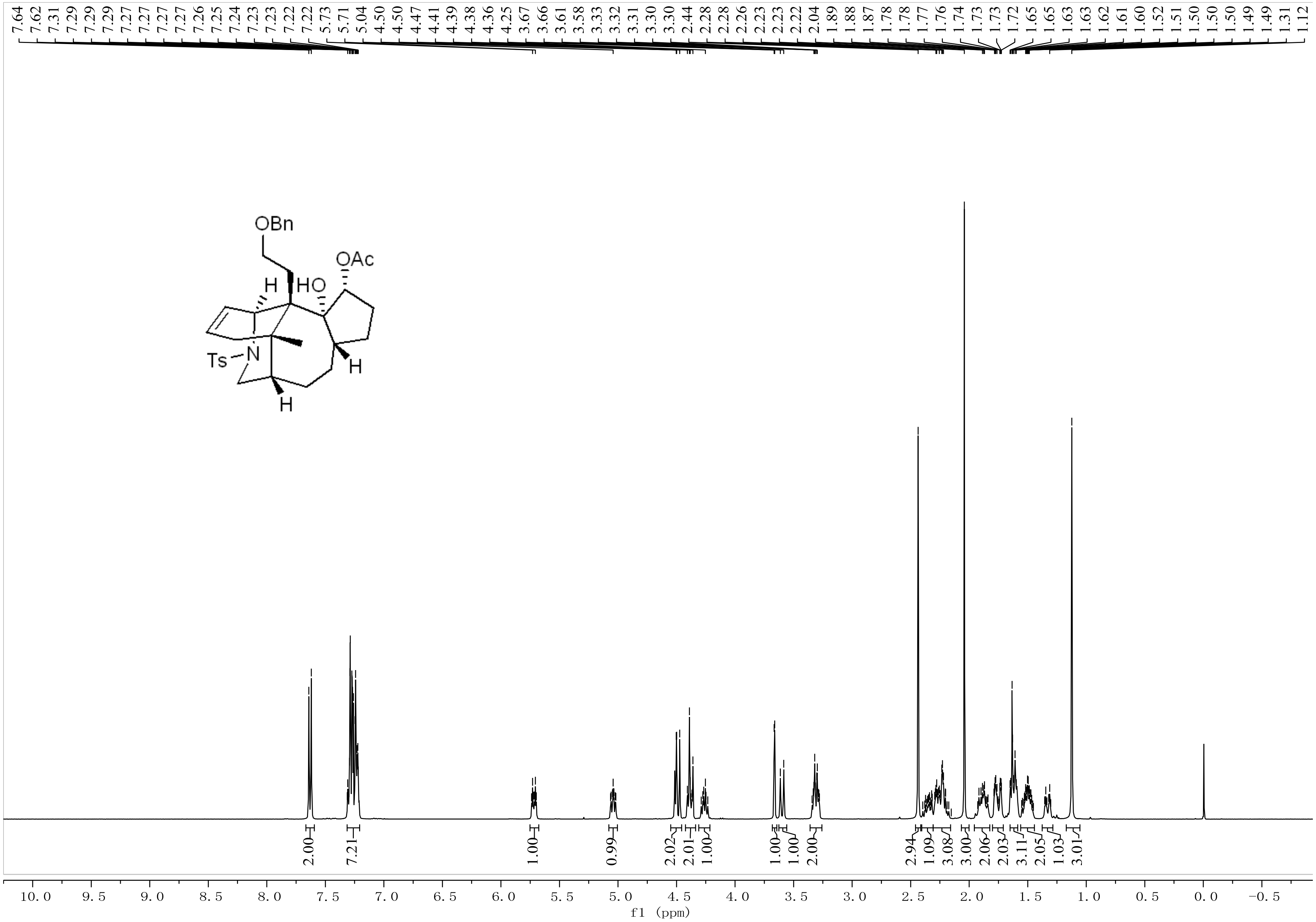

**Supplementary Figure 36.** [1]**H-NMR of compound S4**

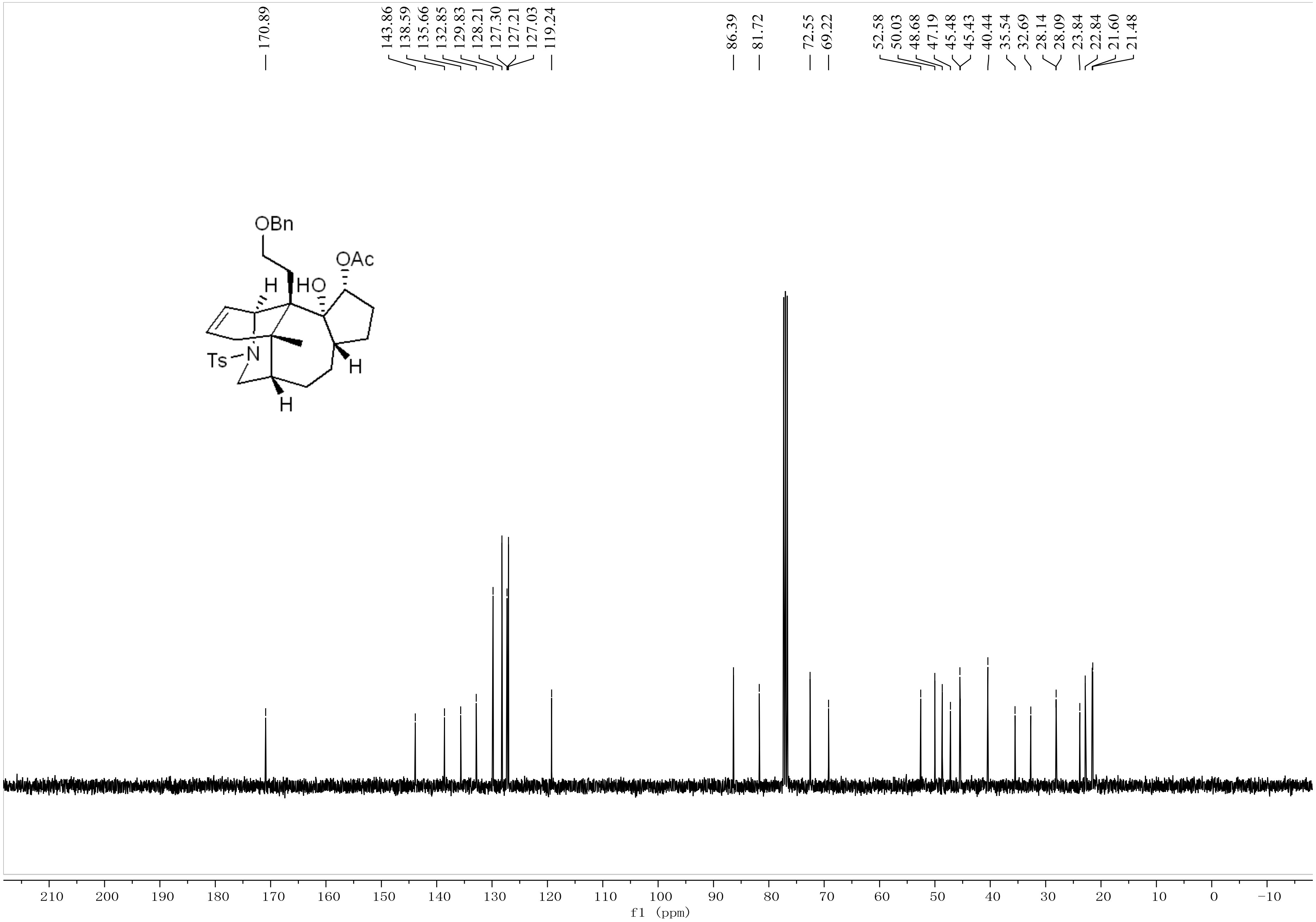


**Supplementary Figure 37.** $^{13}$C-NMR of compound S4

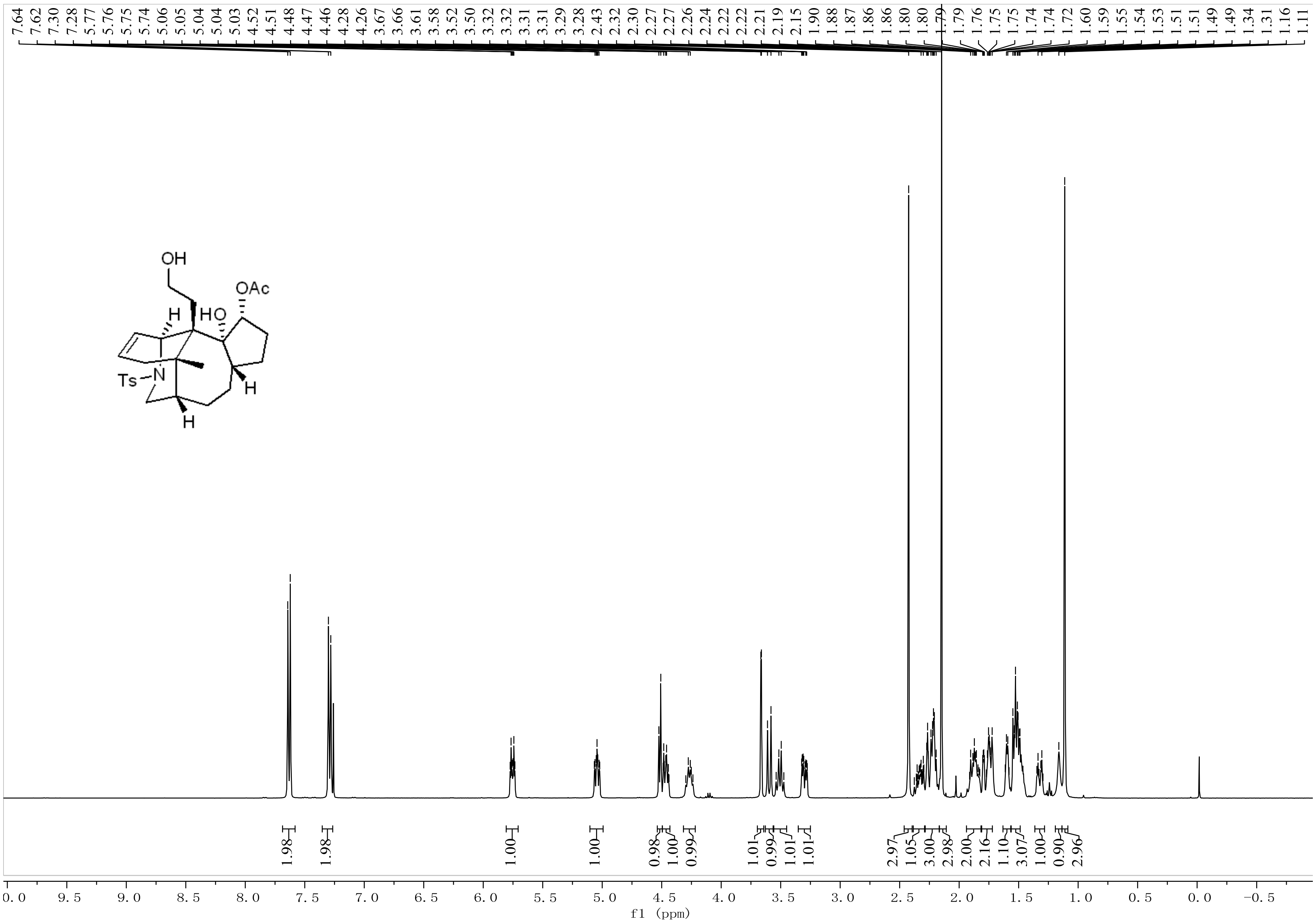

**Supplementary Figure 38.** **$^1$H-NMR of compound 21**

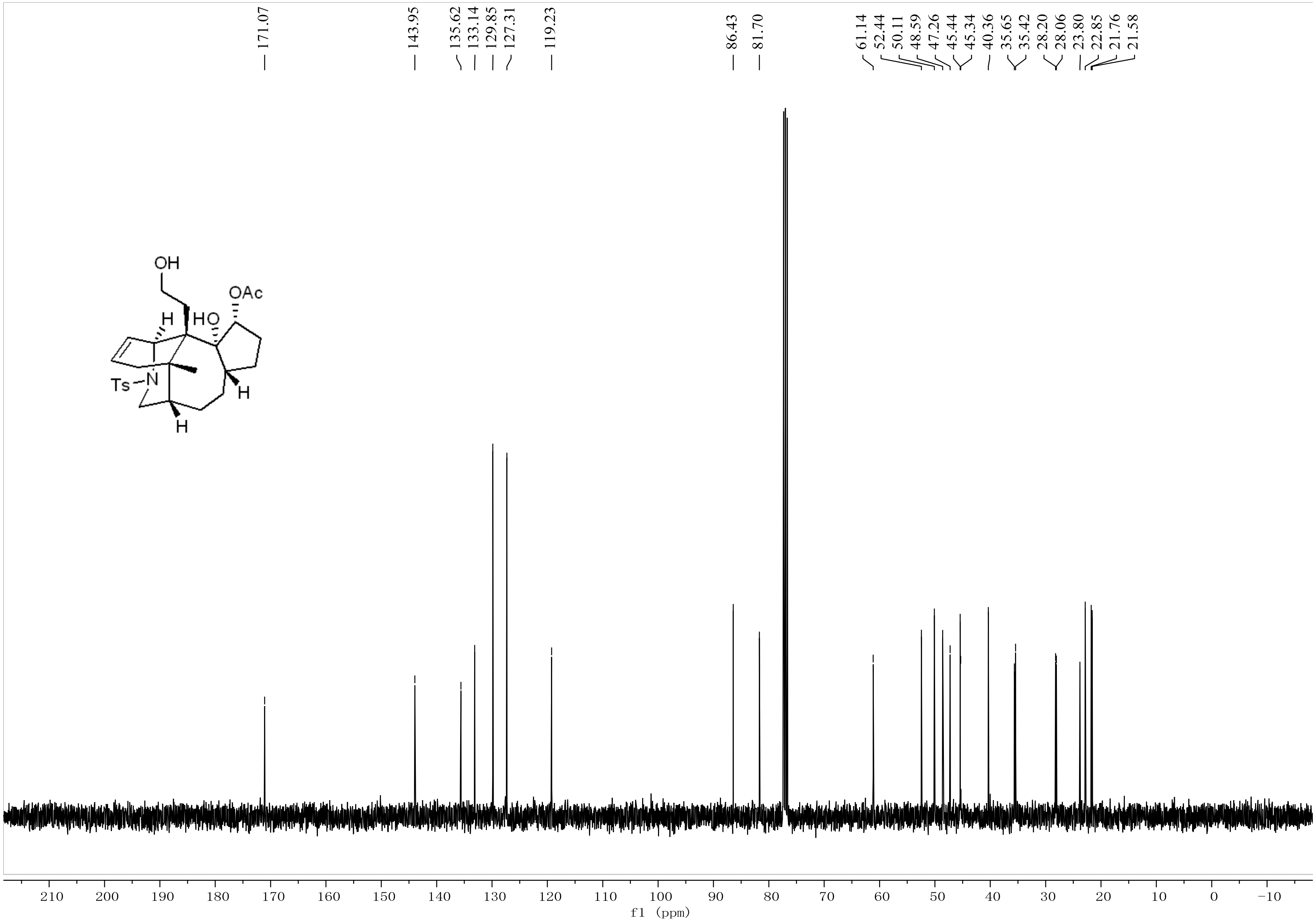


**Supplementary Figure 39.** **$^{13}$C-NMR of compound 21**

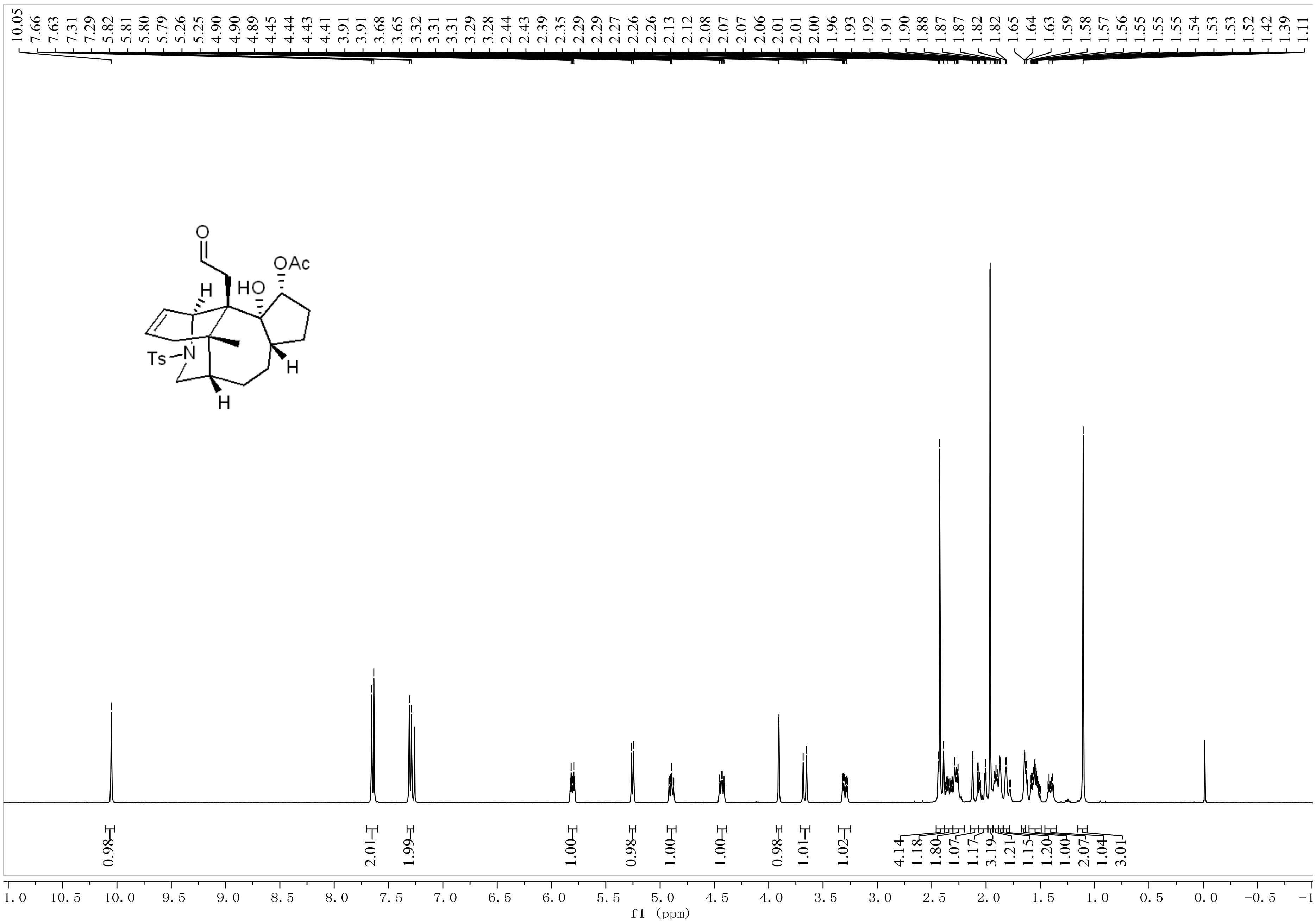

**Supplementary Figure 40.** $^{1}$H-NMR of compound 22

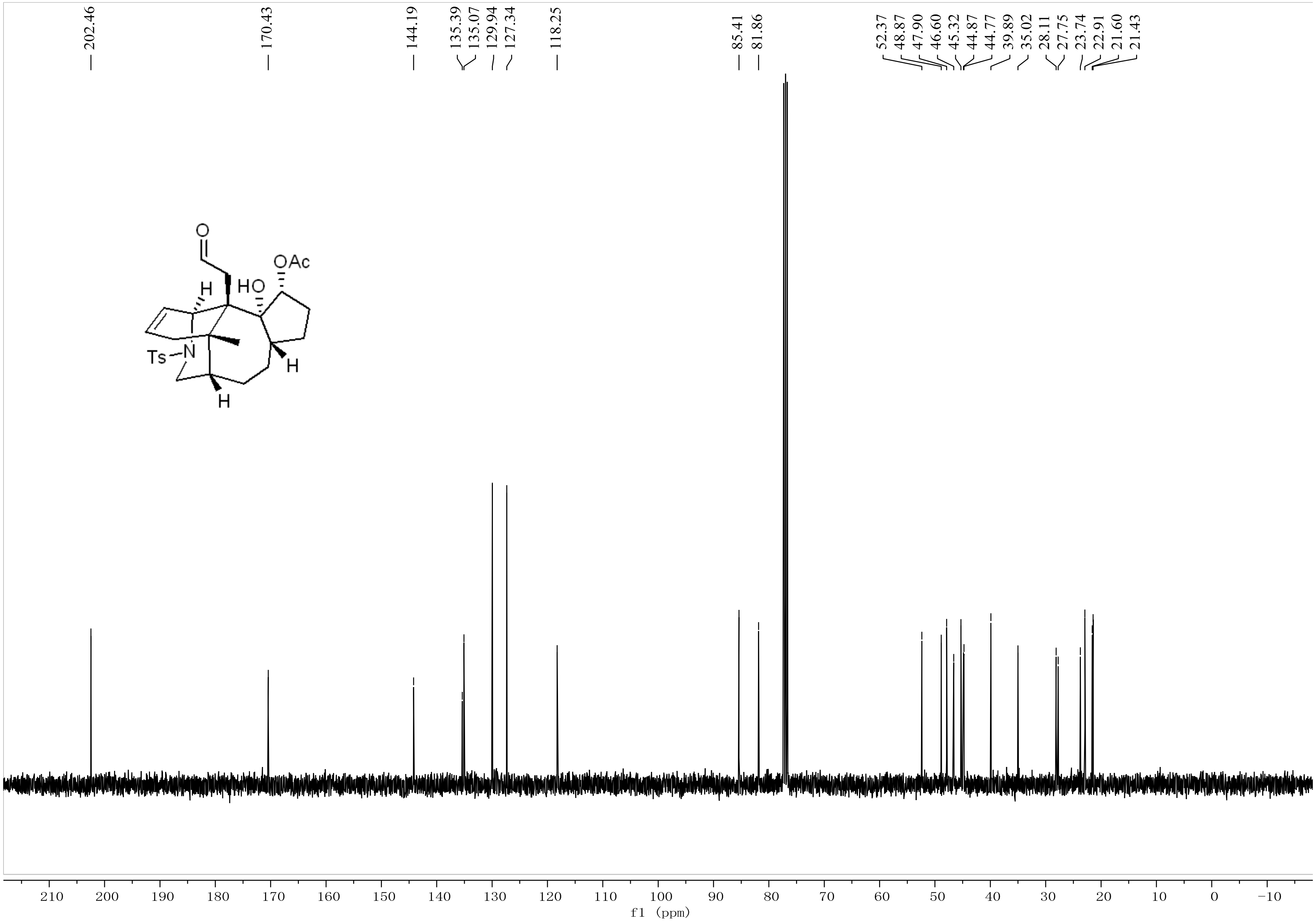


**Supplementary Figure 41.** **$^{13}$C-NMR of compound 22**

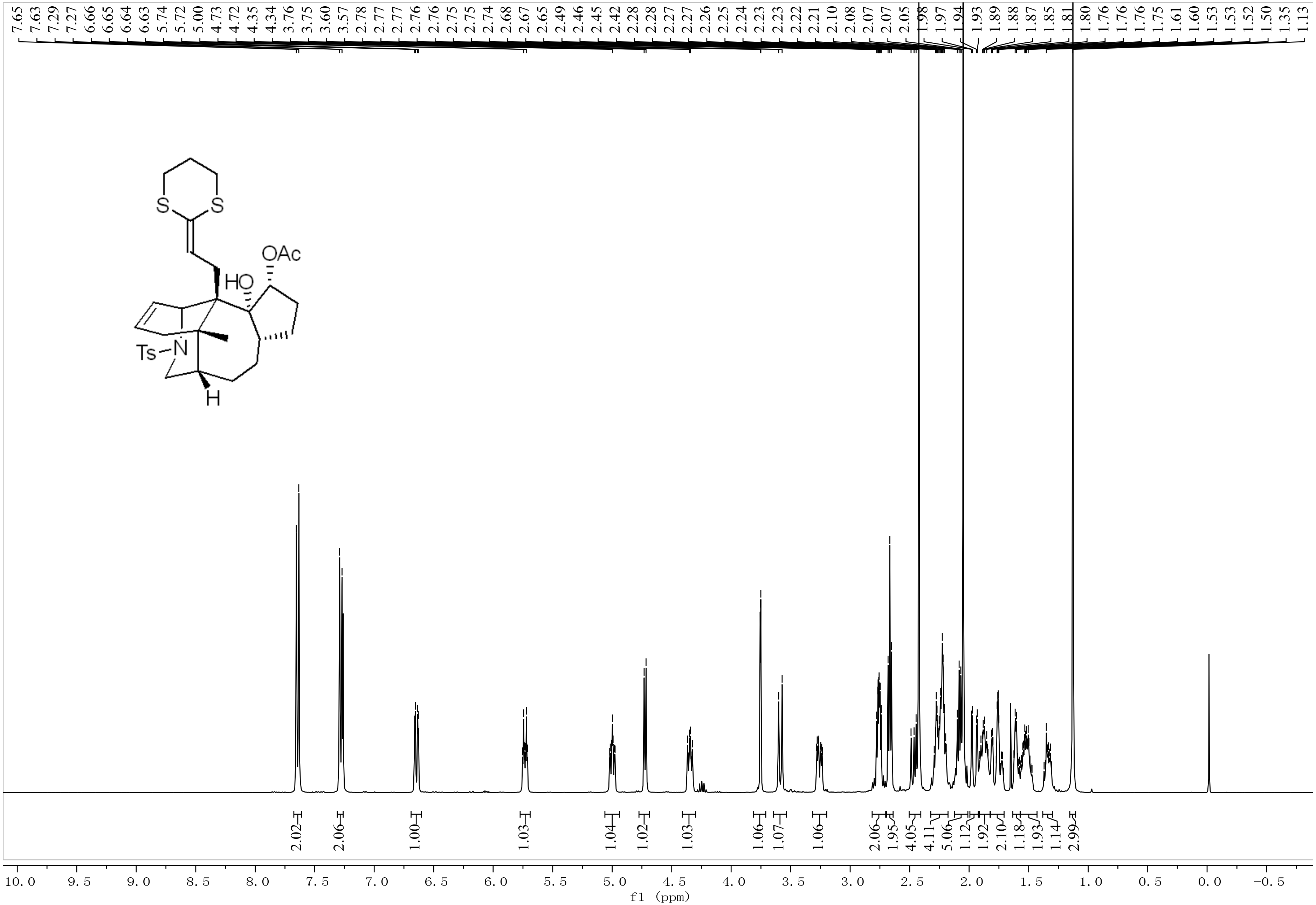

**Supplementary Figure 42.** [1]**H-NMR of compound 24**

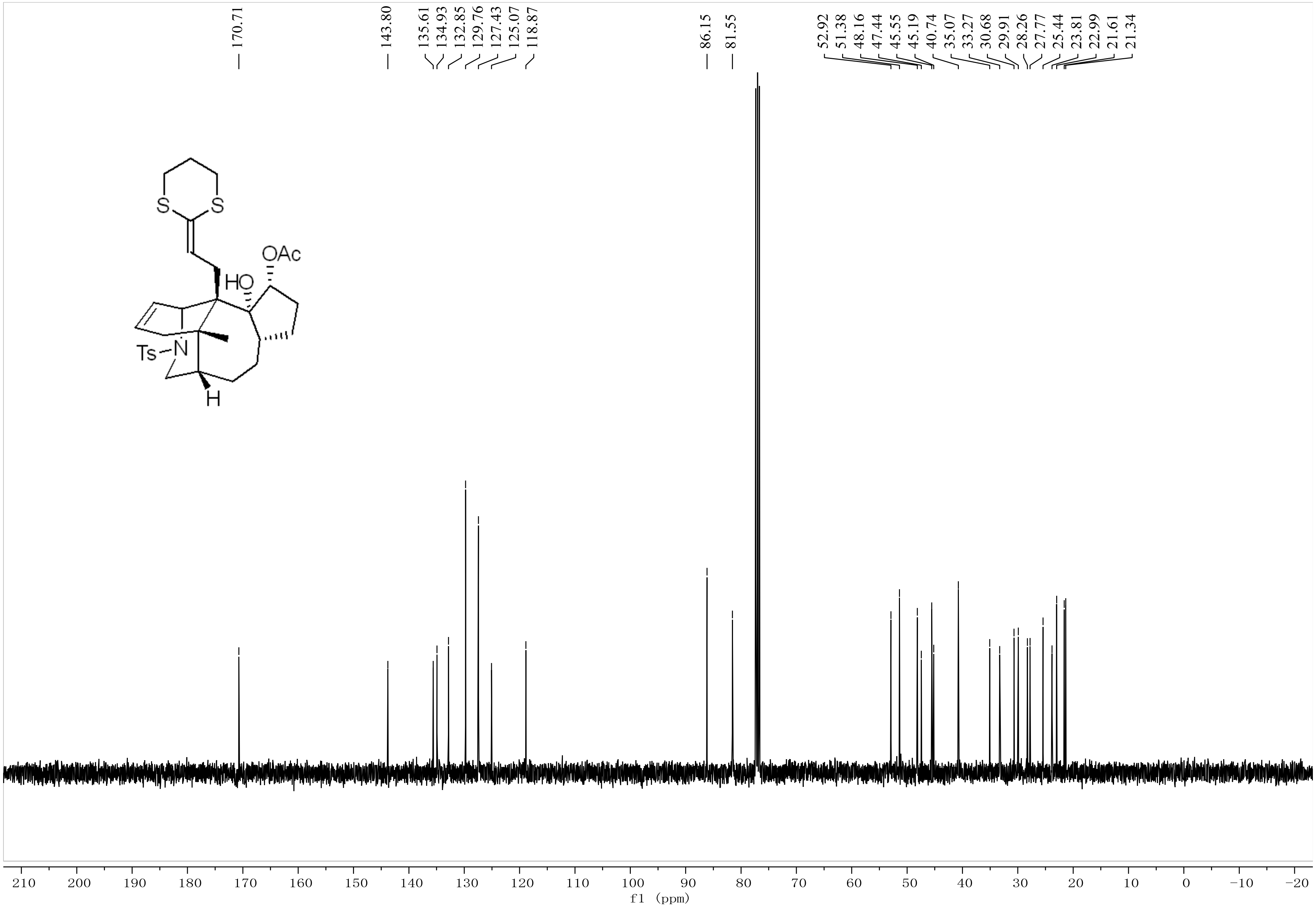


**Supplementary Figure 43.** $^{13}$**C-NMR of compound 22**

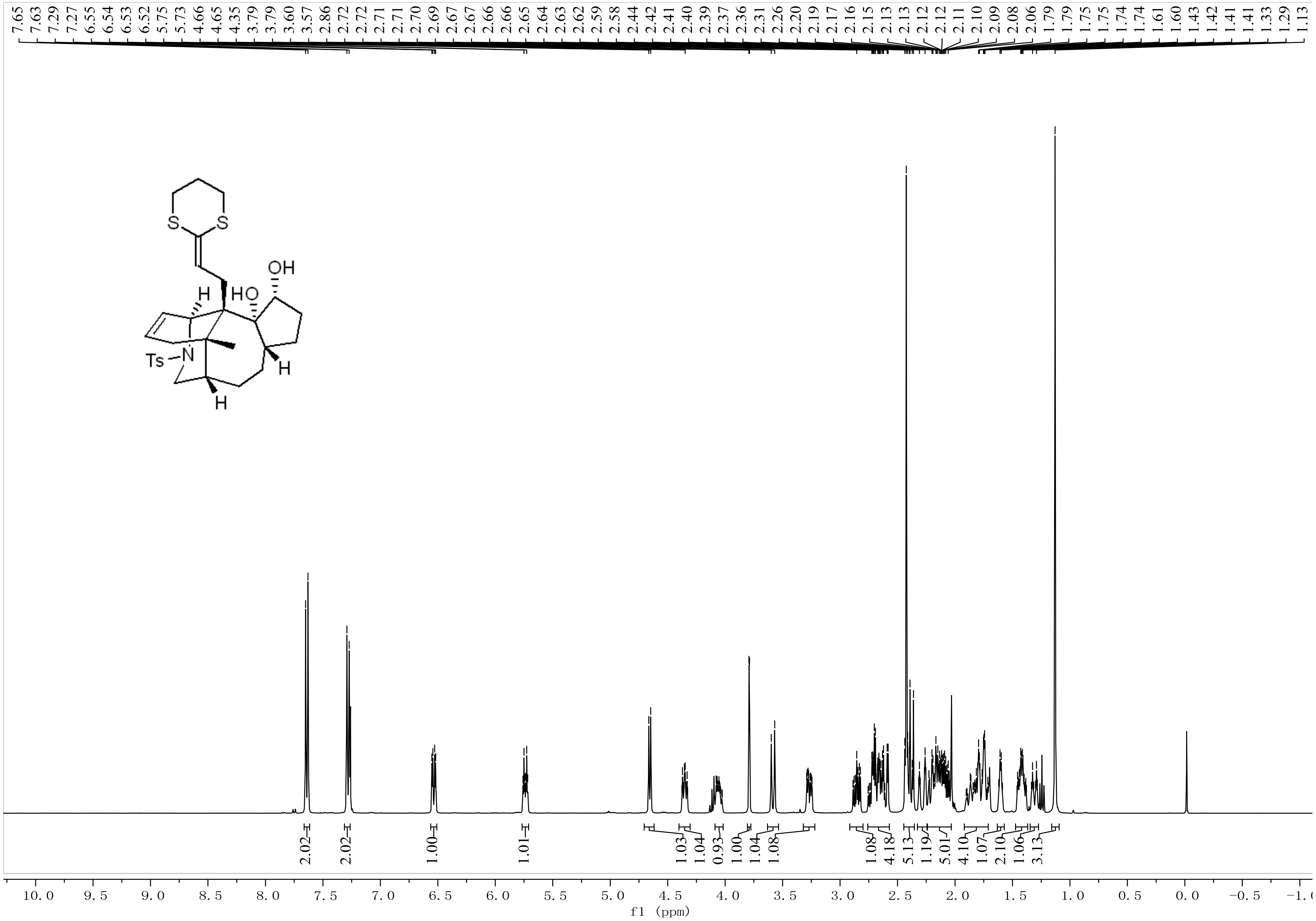

**Supplementary Figure 44.** **¹H-NMR of compound 25**

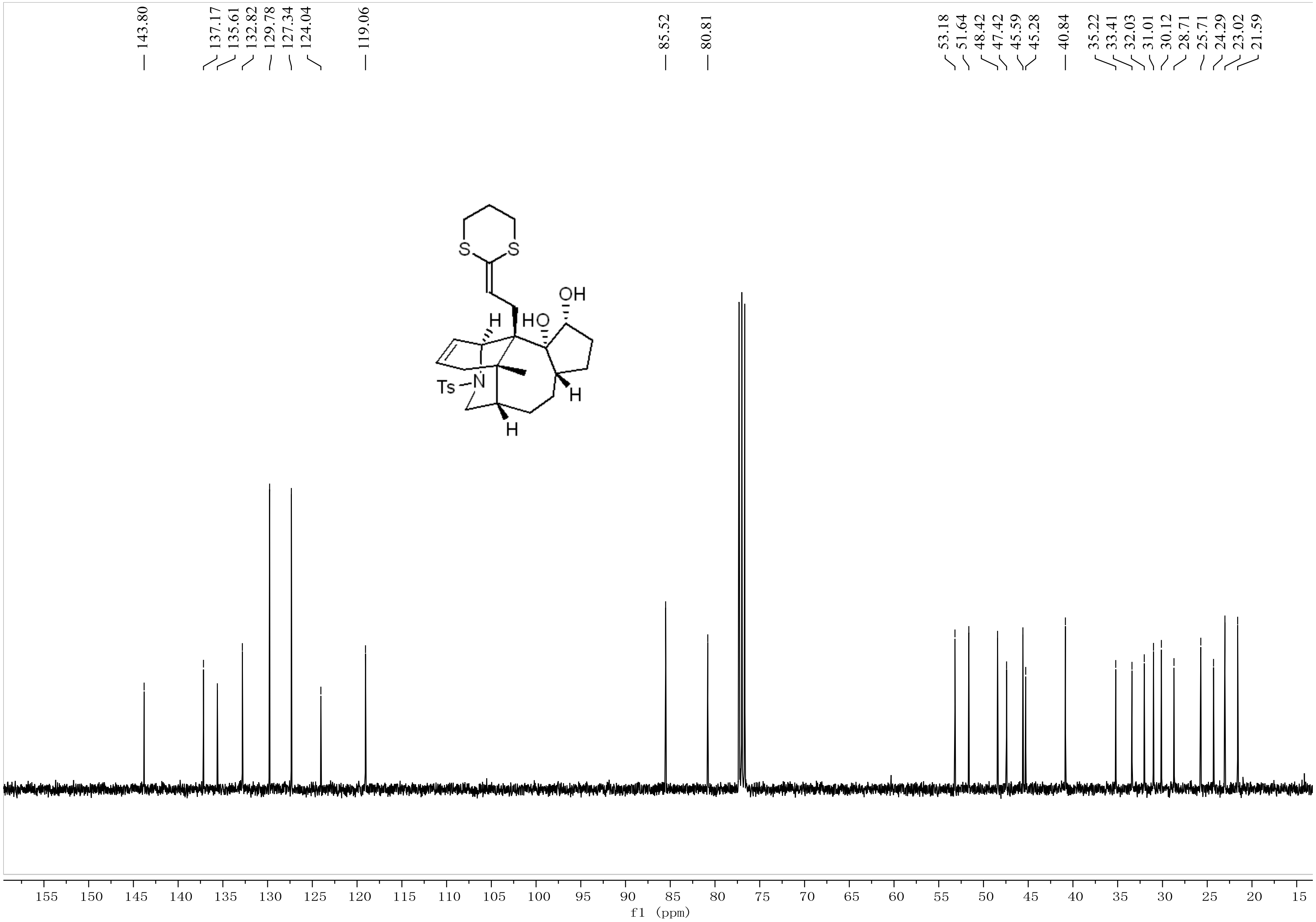


**Supplementary Figure 45.** **$^{13}$C-NMR of compound 25**

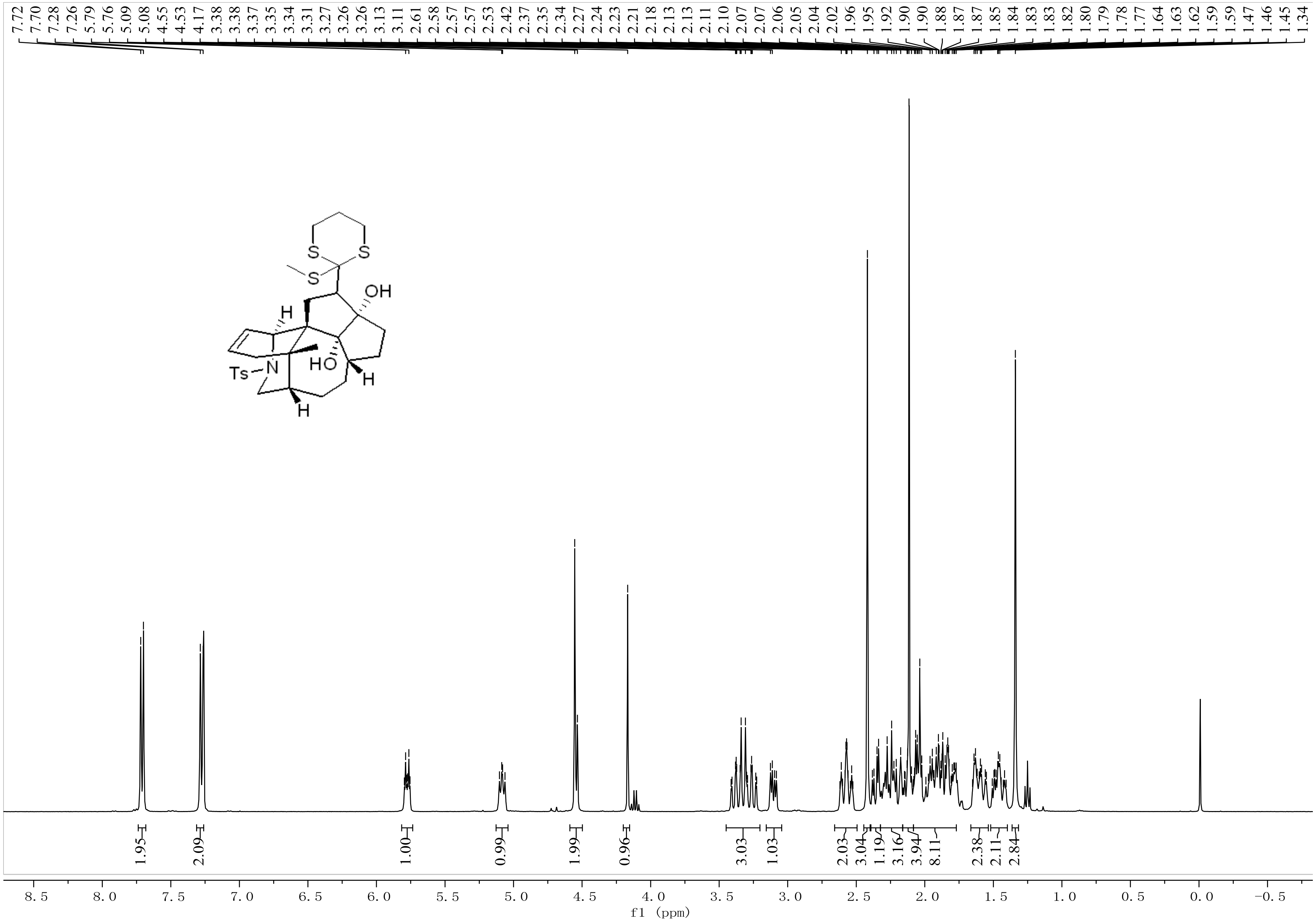

**Supplementary Figure 46.** [1]**H-NMR of compound 26**

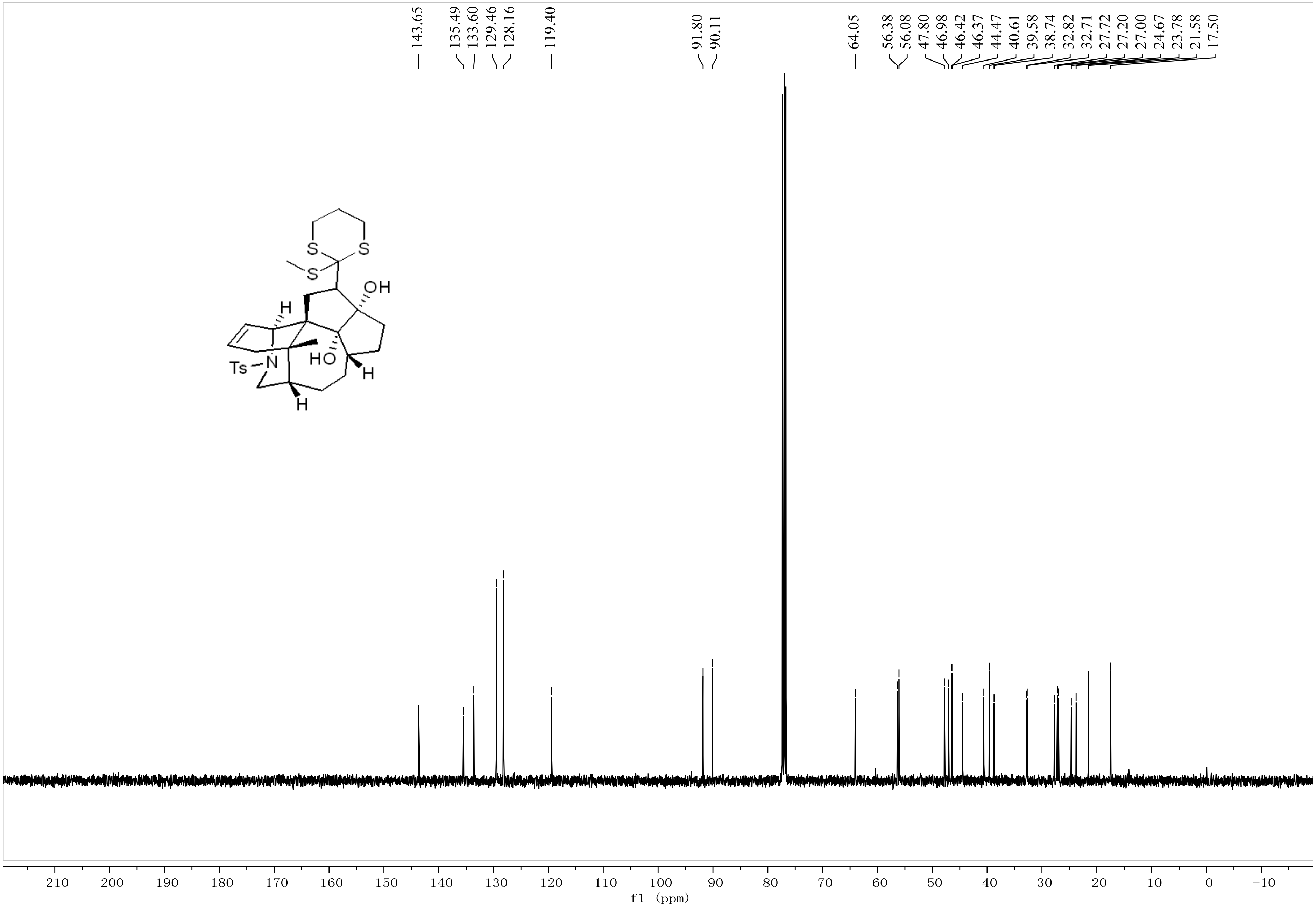


**Supplementary Figure 47.** [13]**C-NMR of compound 26**

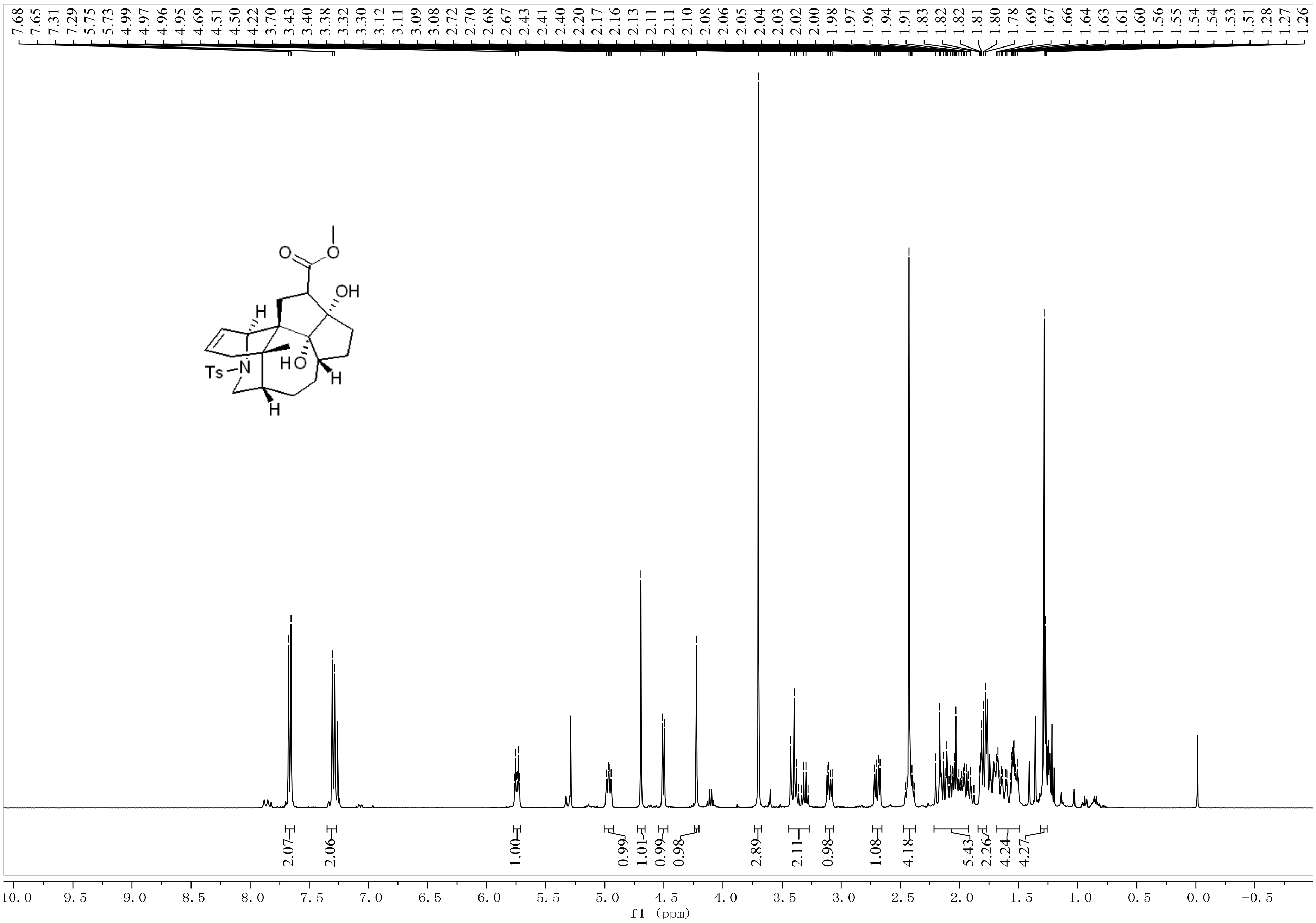

**Supplementary Figure 48.** [1]**H-NMR of compound 27**

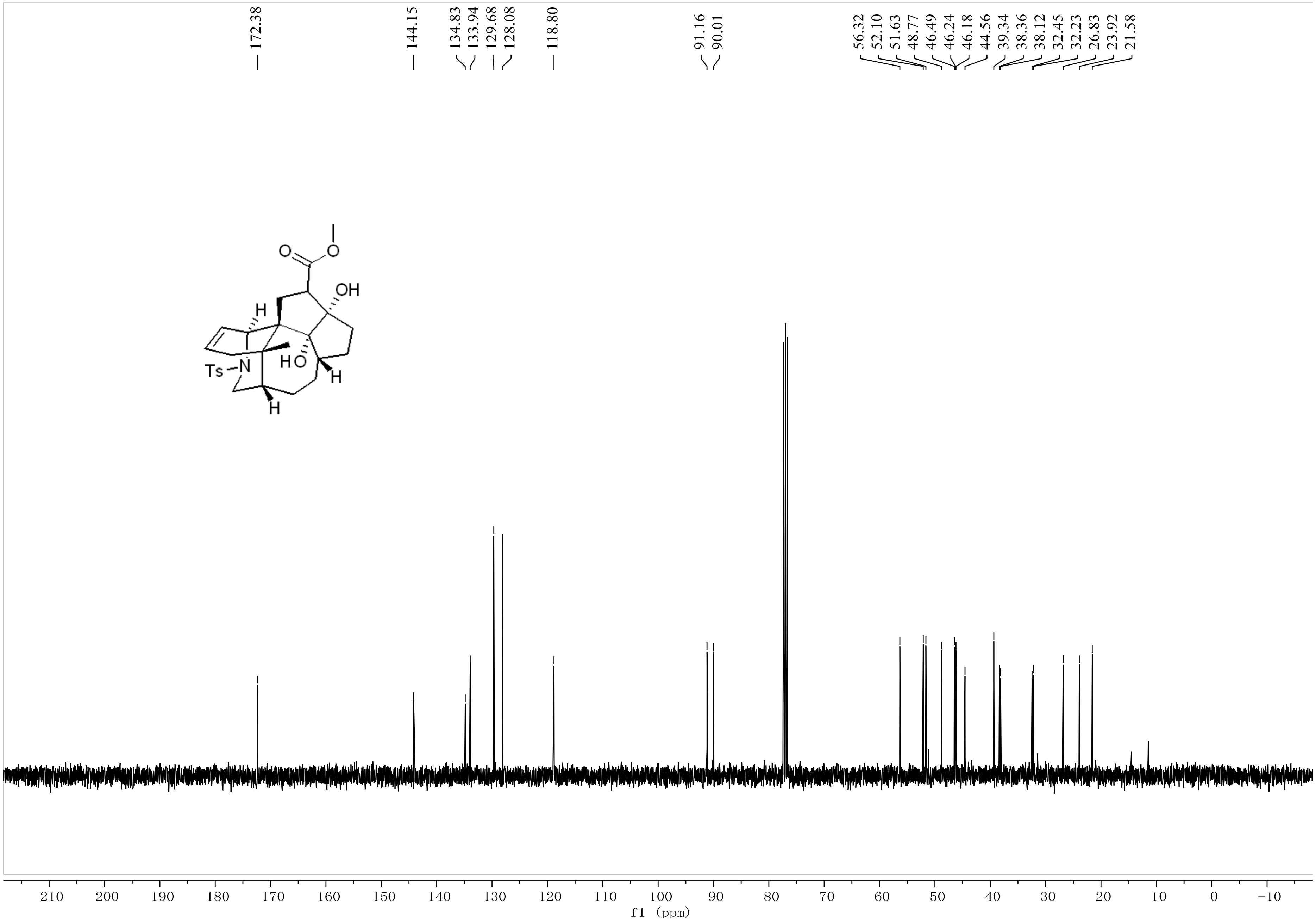


**Supplementary Figure 49.** **$^{13}$C-NMR of compound 27**

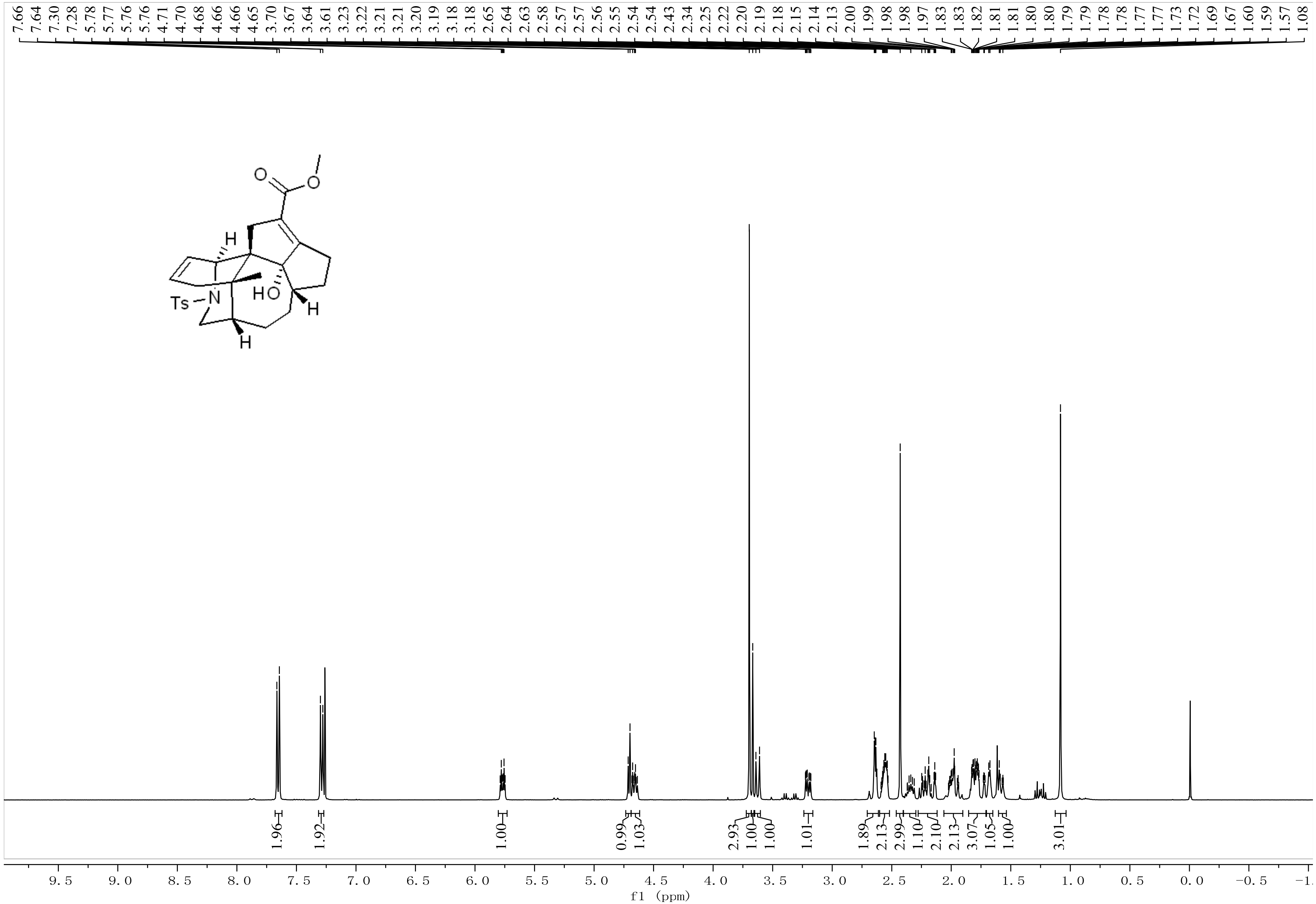

**Supplementary Figure 50.** [1]**H-NMR of compound 29**

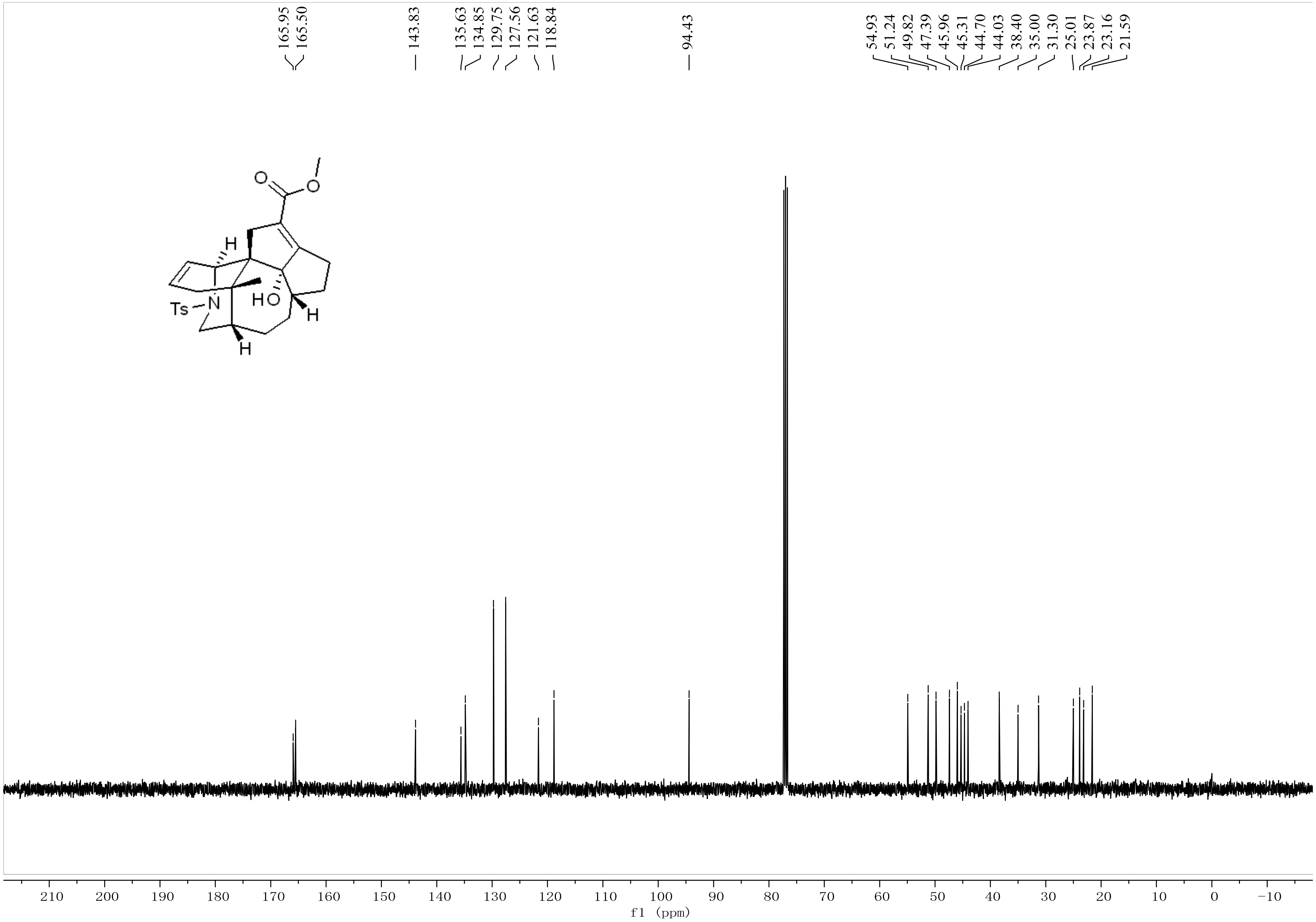


**Supplementary Figure 51.** **$^{13}$C-NMR of compound 29**

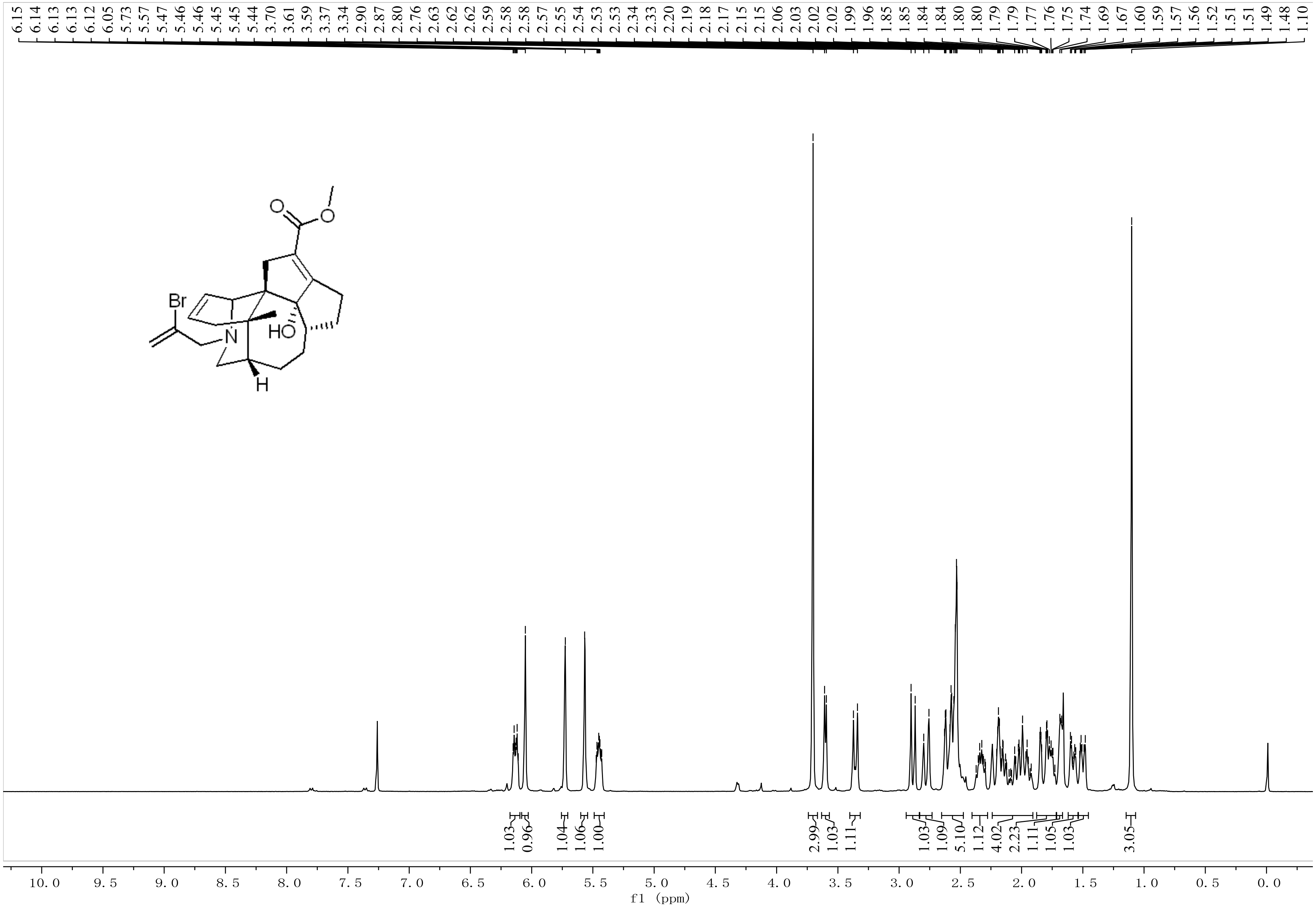

**Supplementary Figure 52.** **$^{1}$H-NMR of compound 30**

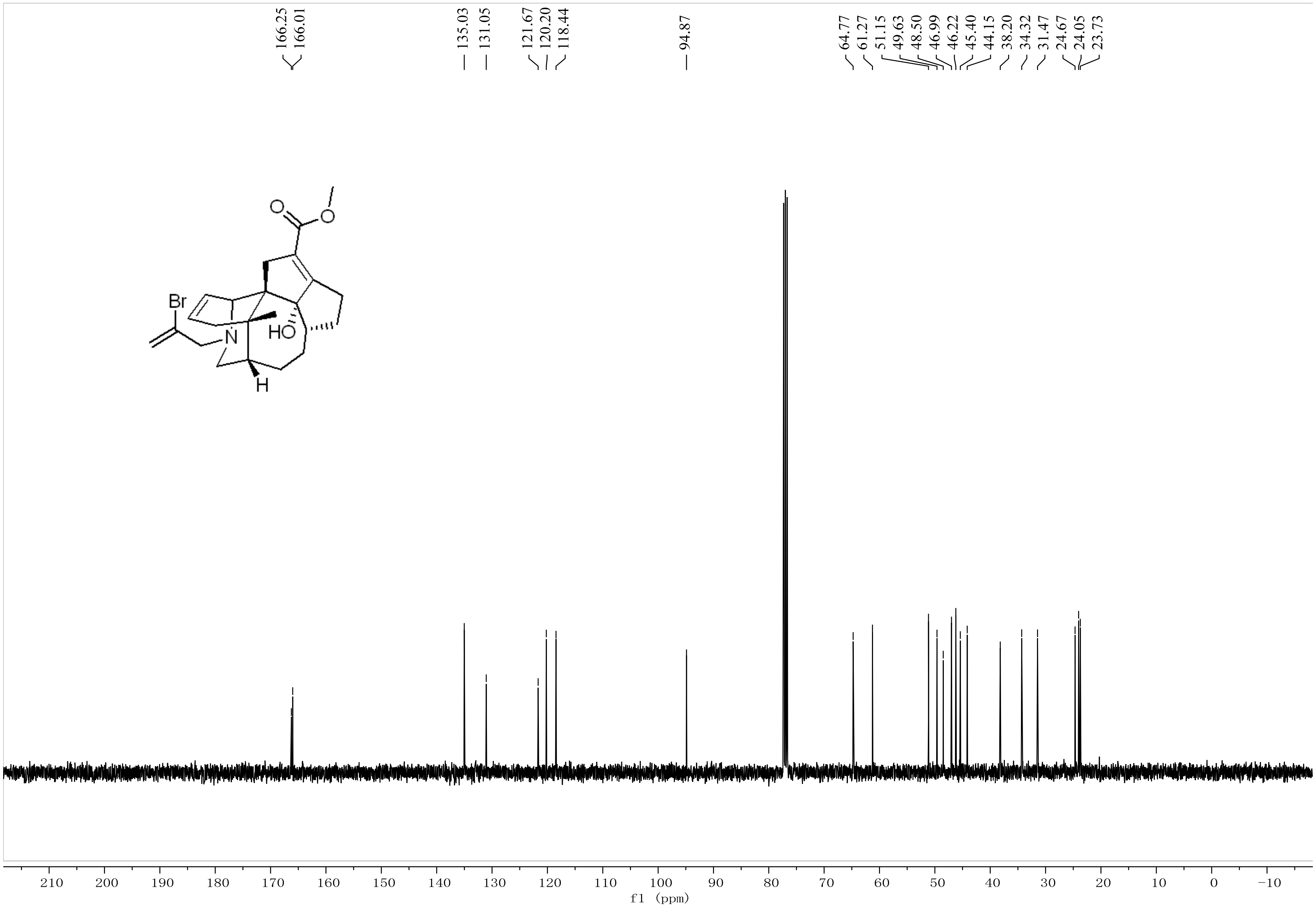


**Supplementary Figure 53.** **$^{13}$C-NMR of compound 30**

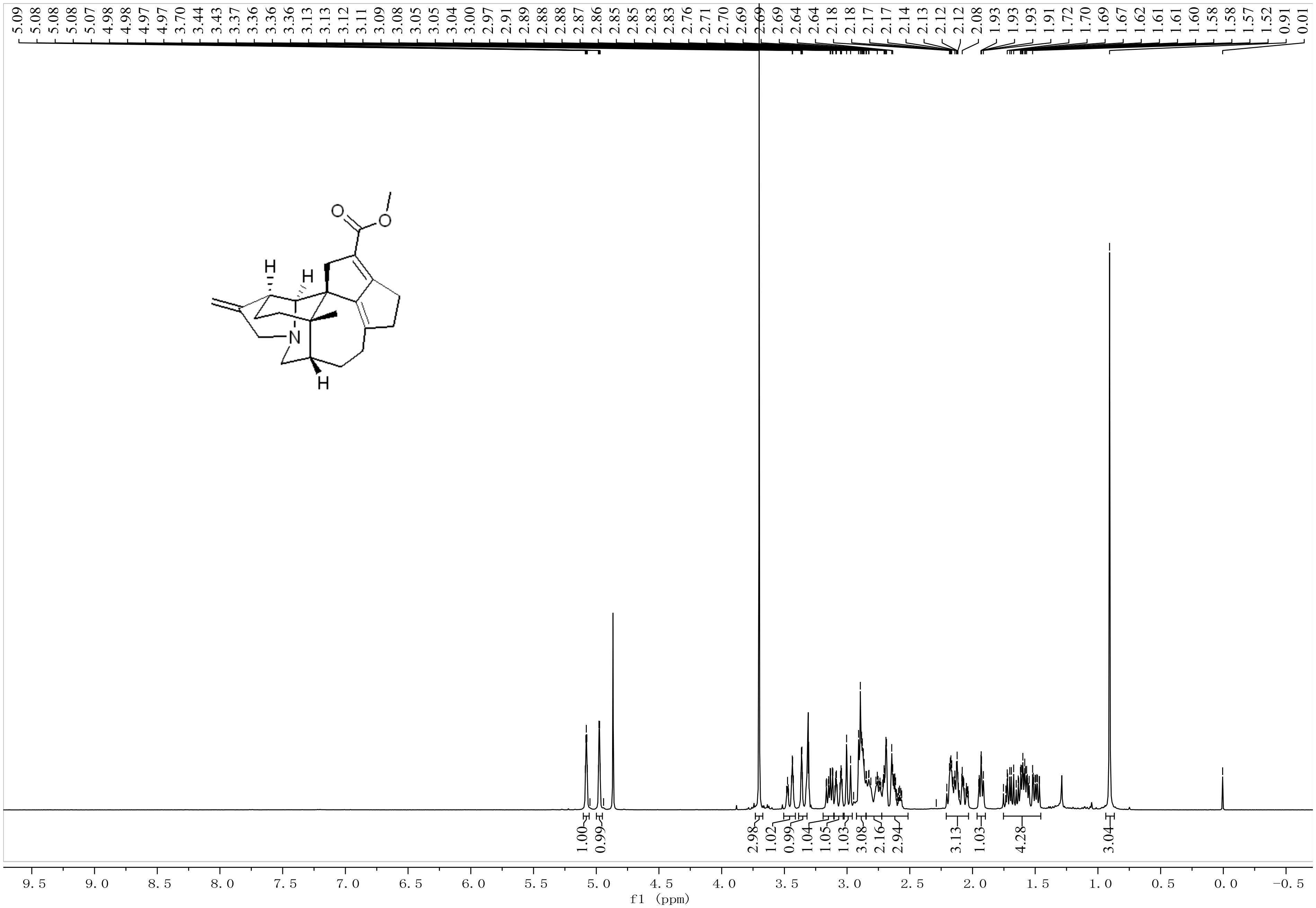

**Supplementary Figure 54.** $^{1}$**H-NMR of compound 31**

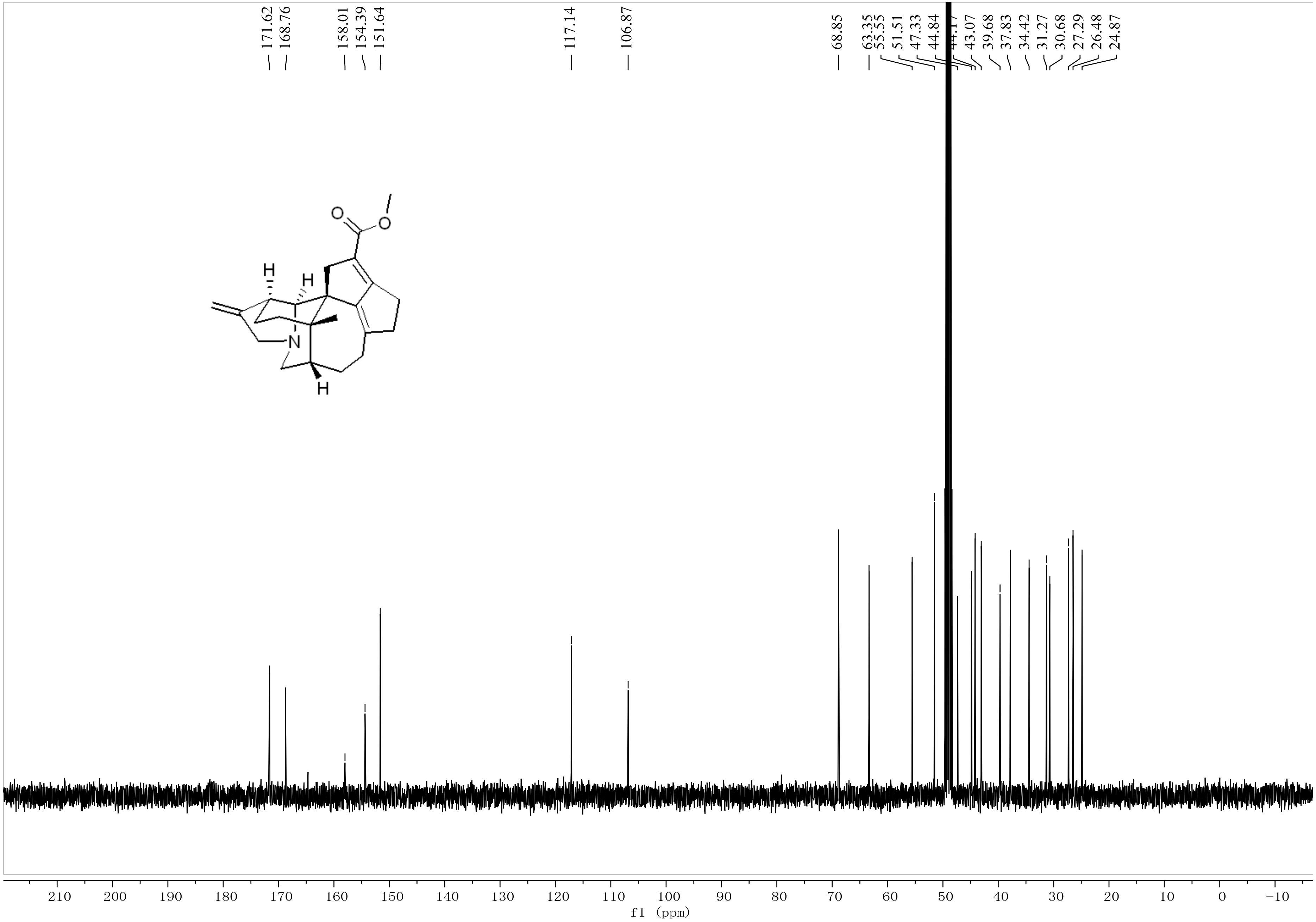


**Supplementary Figure 55.** [13]**C-NMR of compound 31**

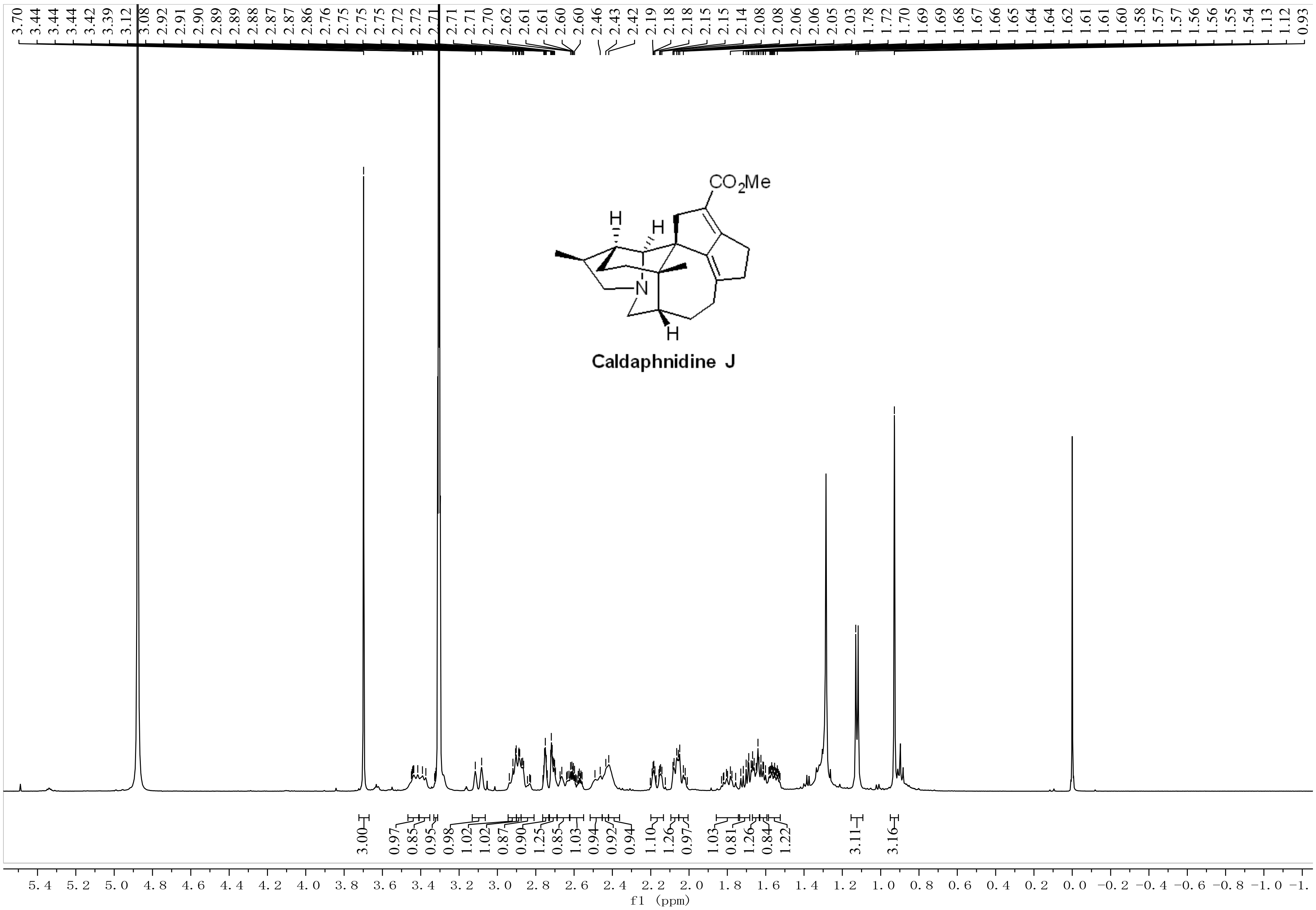


**Supplementary Figure 56.** **$^1$H-NMR of Caldaphnidine J**

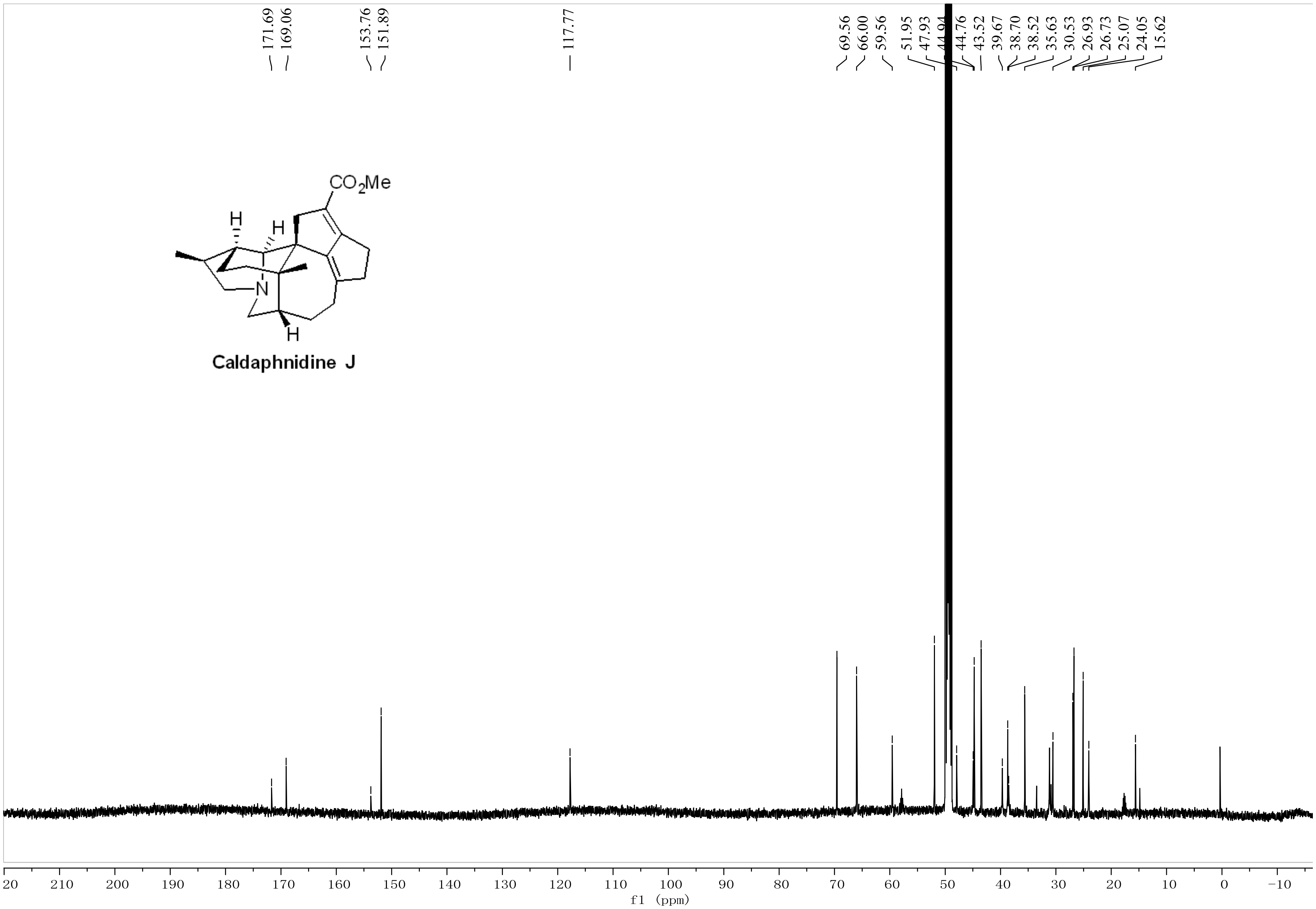


**Supplementary Figure 57.** $^{13}$C-NMR of Caldaphnidine J

# X-Ray Crystallographic Data

The crystal structure and X-ray crystallographic data of compound **22** (CCDC 1986889) was described as follows:

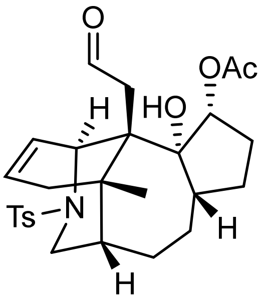


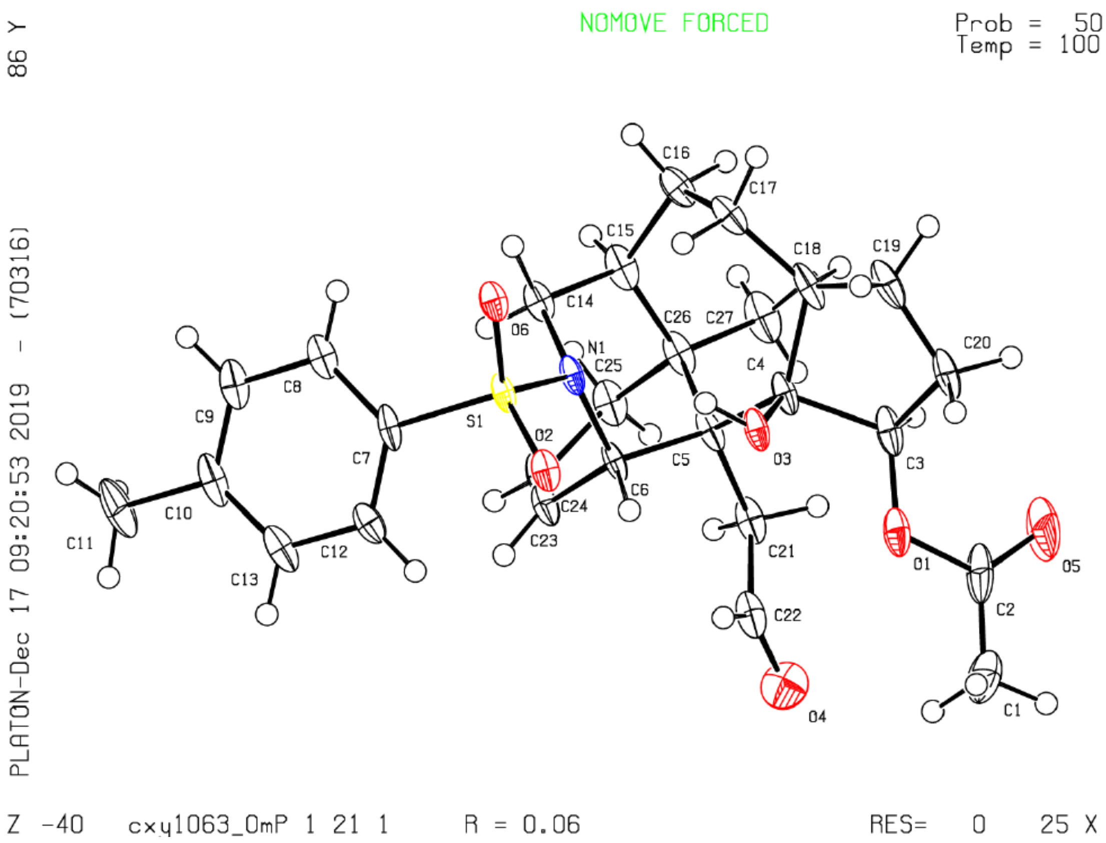


**Supplementary Figure 58.** X-ray crystal structure of **22**

| **Supplementary Table 3 Crystal data and structure refinement for compound 22.** | | | |
|---|---|---|---|
| Bond precision: | C-C = 0.0081 A | Wavelength = 0.71073 | |
| Cell | a = 8.2165(7) | b = 10.5362(9) | c = 14.9894(12) |
| | alpha = 90 | beta = 104.270(3) | gamma = 90 |
| Temperature | 100 K | | |
| | | Calculated | Reported |
| Volume | | 1257.60(18) | 1257.60(18) |
| Space group | | P 21 | P 1 21 1 |
| Hall group | | P 2yb | P 2yb |

| | | |
|---|---|---|
| Moiety formula | C27 H35 N O6 S | C27 H35 N O6 S |
| Sum formula | C27 H35 N O6 S | C27 H35 N O6 S |
| Mr | 501.62 | 500.61 |
| Dx, g $cm^{-3}$ | 1.325 | 1.322 |
| Z | 2 | 2 |
| Mu | 0.172 | 0.171 |
| F000 | 536.0 | 534.0 |
| F000‘ | 536.50 | |
| h, k, $l_{max}$ | 10, 13, 19 | 13, 14, 34 |
| Nref | 5807 [ 3062] | 5790 |
| $T_{min}$, $T_{max}$ | 0.931, 0.948 | 0.463, 0.746 |
| $T_{min}$‘ | 0.931 | |
| Correction method = # Reported T Limits: $T_{min}$ = 0.463 $T_{max}$ = 0.746 | | |
| AbsCorr = MULTI - SCAN | | |
| Data completeness= 1.89/1.00 Theta(max)= 27.532 | | |
| R(reflections)= 0.0645( 4642) wR2(reflections)= 0.1427( 5790) | | |
| S = 1.070 Npar= 320 | | |

The crystal structure and X-ray crystallographic data of compound **24** (CCDC 1986960) was described as follows:

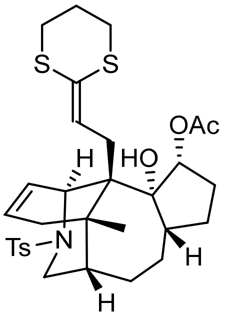


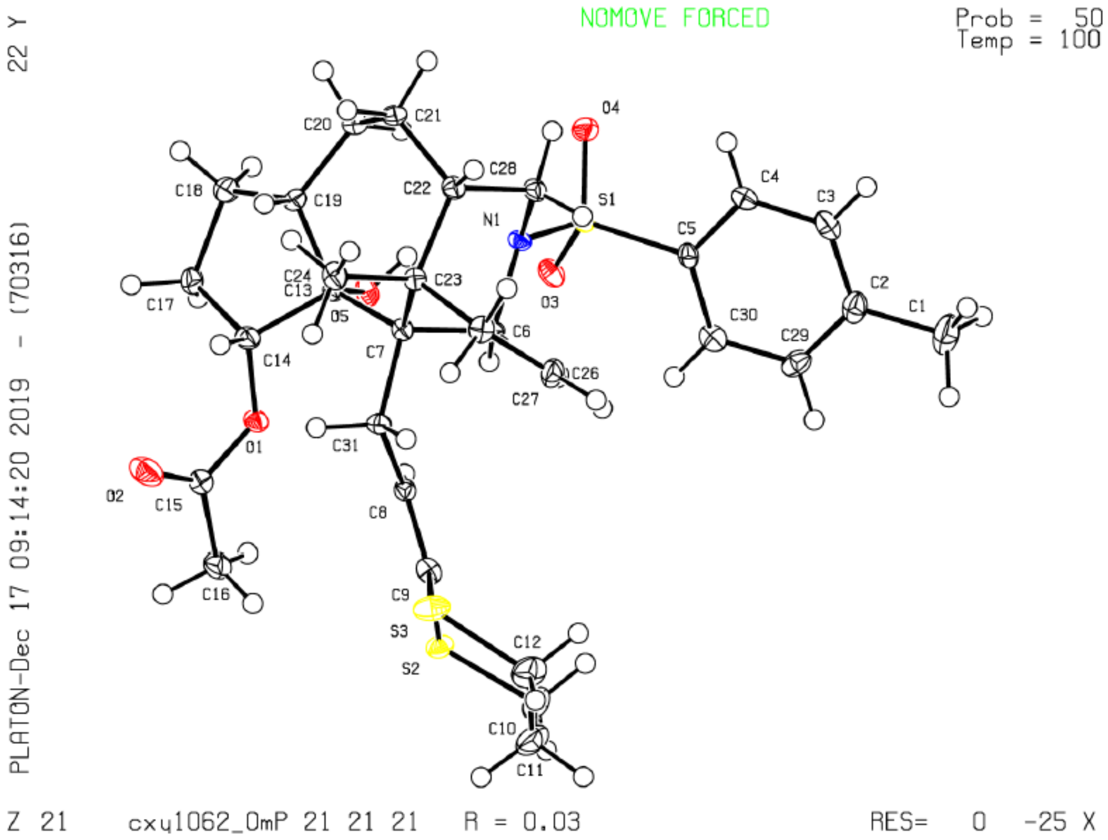


**Supplementary Figure 59.** X-ray crystal structure of **24**

| **Supplementary Table 4.** Crystal data and structure refinement for compound 24. | | | |
|---|---|---|---|
| Bond precision: C-C = 0.0036 A | | Wavelength = 0.71073 | |
| Cell | a = 10.1571(5) | b = 11.2277(6) | c = 26.2761(14) |
| | alpha = 90 | beta = 90 | gamma = 90 |
| Temperature | 100 K | | |
| | Calculated | Reported | |
| Volume | 2996.3 (3) | 2996.5 (3) | |
| Space group | P 21 21 21 | P 21 21 21 | |
| Hall group | P 2ac 2ab | P 2ac 2ab | |
| Moiety formula | C31 H41 N O5 S3 | C31 H41 N O5 S3 | |
| Sum formula | C31 H41 N O5 S3 | C31 H41 N O5 S3 | |

| | | |
|---|---|---|
| Mr | 603.83 | 603.83 |
| Dx, g cm$^{-3}$ | 1.338 | 1.338 |
| Z | 4 | 4 |
| Mu | 0.288 | 0.288 |
| F000 | 1288.0 | 1288.0 |
| F000' | 1290.00 | |
| h, k, $l_{max}$ | 13, 14, 34 | 13, 14, 34 |
| Nref | 6936 [ 3904] | 6909 |
| $T_{min}$, $T_{max}$ | 0.908,0.928 | 0.697,0.746 |
| $T_{min}$' | 0.894 | |
| Correction method= # Reported T Limits: $T_{min}$=0.697 $T_{max}$=0.746 | | |
| AbsCorr = MULTI-SCAN | | |
| Data completeness= 1.77/1.00 Theta(max)= 27.570 | | |
| R(reflections)= 0.0338( 6601) wR2(reflections)= 0.0803( 6909) | | |
| S = 1.094 Npar= 366 | | |

## Supplementary References


1. Guo, L.-D. *et al.* Total Synthesis of Dapholdhamine B and Dapholdhamine B Lactone. *J. Am. Chem. Soc.* **141**, 11713−11720 (2019).
2. Guo, L.-D. *et al.* Enantioselective Total Synthesis of (−)-Caldaphnidine O via a Radical Cyclization Cascade. *J. Am. Chem. Soc.* **141**, 13043−13048 (2019).
3. Zhang, C.-R., Yang, S.-P. & Yue, J.-M. Alkaloids from the twigs of *Daphniphyllum Calycinum*. *J. Nat. Prod.* **71**, 1663−1668 (2008).
